# Health Information on Pre-Exposure Prophylaxis From Search Engines and Twitter: Readability Analysis

**DOI:** 10.2196/48630

**Published:** 2023-09-04

**Authors:** Albert Park, Fatima Sayed, Patrick Robinson, Latesha Elopre, Yaorong Ge, Shaoyu Li, Christian Grov, Patrick Sean Sullivan

**Affiliations:** 1 Department of Software and Information Systems University of North Carolina Charlotte Charlotte, NC United States; 2 Department of Public Health Sciences University of North Carolina Charlotte Charlotte, NC United States; 3 Division of Infectious Disease University of Alabama at Birmingham Birmingham, AL United States; 4 Department of Mathematics and Statistics University of North Carolina Charlotte Charlotte, NC United States; 5 Department of Community Health and Social Sciences City University of New York New York City, NY United States; 6 Department of Epidemiology Emory University Atlanta, GA United States

**Keywords:** pre-exposure prophylaxis, PrEP, health literacy, health education materials, readability, prophylaxis, health information, electronic health education, HIV, infection, Twitter

## Abstract

**Background:**

Pre-exposure prophylaxis (PrEP) is proven to prevent HIV infection. However, PrEP uptake to date has been limited and inequitable. Analyzing the readability of existing PrEP-related information is important to understand the potential impact of available PrEP information on PrEP uptake and identify opportunities to improve PrEP-related education and communication.

**Objective:**

We examined the readability of web-based PrEP information identified using search engines and on Twitter. We investigated the readability of web-based PrEP documents, stratified by how the PrEP document was obtained on the web, information source, document format and communication method, PrEP modality, and intended audience.

**Methods:**

Web-based PrEP information in English was systematically identified using search engines and the Twitter API. We manually verified and categorized results and described the method used to obtain information, information source, document format and communication method, PrEP modality, and intended audience. Documents were converted to plain text for the analysis and readability of the collected documents was assessed using 4 readability indices. We conducted pairwise comparisons of readability based on how the PrEP document was obtained on the web, information source, document format, communication method, PrEP modality, and intended audience, then adjusted for multiple comparisons.

**Results:**

A total of 463 documents were identified. Overall, the readability of web-based PrEP information was at a higher level (10.2-grade reading level) than what is recommended for health information provided to the general public (ninth-grade reading level, as suggested by the Department of Health and Human Services). Brochures (n=33, 7% of all identified resources) were the only type of PrEP materials that achieved the target of ninth-grade reading level.

**Conclusions:**

Web-based PrEP information is often written at a complex level for potential and current PrEP users to understand. This may hinder PrEP uptake for some people who would benefit from it. The readability of PrEP-related information found on the web should be improved to align more closely with health communication guidelines for reading level to improve access to this important health information, facilitate informed decisions by those with a need for PrEP, and realize national prevention goals for PrEP uptake and reducing new HIV infections in the United States.

## Introduction

Pre-exposure prophylaxis (PrEP) is proven to prevent HIV infections [1-3], and using PrEP to prevent new HIV infections among vulnerable populations has been identified as a critical strategy in the Ending the HIV Epidemic initiative [4]. PrEP use increased more than 10-fold in the United States in just 5 years after its approval in 2012 [5]. However, inequitable PrEP uptake persists in the United States [6-8], especially in the southern United States and among Black and Hispanic residents in the United States. When those who need PrEP the most do not receive it, we lose opportunities to maximize reductions in new HIV infections. One potential explanation for lower uptake among vulnerable populations for HIV infections is the lower level of health literacy in these groups (ie, people in the southern United States, people living in poverty, younger people, and people of Black race or Hispanic ethnicity) [9,10]. Thus, analyzing the readability (ie, how difficult a text is to understand) of web-based information about PrEP would help understand the effectiveness of available web-based PrEP information and identify opportunities to improve the understandability of information for people who might benefit from PrEP.

Health literacy is an individual’s ability to obtain and understand health information to make informed health decisions [11]. The US Department of Health and Human Services (HHS) [12,13], the National Institutes of Health (NIH) [13,14], the American Medical Association (AMA) [15], and the US Food and Drug Administration (FDA) [16] recommend health information for the public be written at the ninth-grade reading level or lower to ensure that most people in the US population (ie, the general public) can make informed health decisions. However, researchers have consistently found evidence that text-based health information resources, including web-based resources, are too complex for the general public [17-25]. For example, patient education materials for electronic cigarettes [22], mental health [17], pediatrics [23], medical specialties [24], diabetes mellitus [18], clinical orthopedics [19], human papillomavirus immunizations [20], and cardiovascular diseases [21] were found to require higher literacy levels than that recommended by the NIH, AMA, FDA, and HHS. Moreover, health information available from commercially funded sources was significantly more difficult to read than the information available from government-funded sources for some health conditions, such as diabetes, hypertension, depression, high cholesterol, arthritis, asthma, heartburn, obesity, influenza, and erectile dysfunction [26], but not for electronic cigarette information [22]. This complexity often led to comprehension errors [27,28].

The readability of web-based PrEP information has also been reported [25], but the previous assessment was limited to 100 unique websites and did not include information circulated on social media. Additionally, the previous assessment did not stratify the assessment, which limits our understanding of how to improve the readability of educational materials. For information related to PrEP use, the increasing complexity of educational materials has been associated with lower uptake of PrEP [29,30]. There are other factors that can impact the understandability of PrEP information and accessibility to certain populations. For example, the format of the information (eg, brochure and website), the information source (eg, US government entities and for-profit organizations), and the intended audience could impact the readability of and trust in materials. Thus, different forms and sources of information should be examined for readability; these data should be interpreted in light of intended audiences who might require different levels of complexity. Analyzing updated content is also important because new PrEP delivery methods continue to gain approval and become available to consumers. For example, with the recent approval of injectable PrEP [31], readability studies of PrEP information need to be updated to include this new delivery method.

Assessments of readability should also include information disseminated through social media [32,33], which is an important source of passive information for many young people. However, many recent studies on the readability of health-related content on the web [17-25] have either focused solely on information obtained actively through search engines [17,18,20-22,24,25] or within popular websites for consumers [19,23]. It is important to examine health information that is obtained both actively via search engines and passively via social media channels. The latter is critical because >80% of Americans use at least one social media platform [34] and major health agencies, such as World Health Organization (WHO) and Centers for Disease Control and Prevention (CDC), regularly communicate via Twitter feeds. Social media content can include news releases as well as discussions on various health topics (eg, sexually transmitted infections, emergency situations [35,36], and preventive measures [33]).

We aimed to improve our understanding of the readability of publicly available web-based and social media–based PrEP information by analyzing it based on its information source, document format, communication method, PrEP modality, and intended audience. Our findings can be used to develop communication strategies that are tailored to the needs of different objectives and improve health literacy and the understandability of PrEP information for the general public.

## Methods

### Data Collection

We collected examples of both passively (eg, social media posts) and actively (information sought through user-initiated web searches) obtained information.

#### Collecting Passively Obtained Information

For passively obtained information, we collected PrEP-related information exchanged through Twitter. Twitter was chosen because many health organizations currently use Twitter to communicate health messages, including information about PrEP and HIV prevention, to the public [37,38]. About 22% of US adults use Twitter (45% of 18- to 24-year-olds); many (42%) check the platform more than once a day [39]. Tweets are short, being limited to 280 characters, but often refer readers to longer text information through embedded URLs. Thus, we investigated patient-level information sources (ie, brochures and websites) that were disseminated by URLs in tweets. We assessed and compared the readability of PrEP information based on the method used to obtain information (ie, passively or actively), information source (ie, organization type), document format (eg, brochures and websites), PrEP modality (ie, oral and injectable), and the intended audience (ie, patient, provider, and general).

To assess passively obtained information on PrEP, we used PrEP-related tweets with URLs as a starting point. Using Twitter API [40], we first collected PrEP-related tweets from July 2012, when the FDA first approved the PrEP indication for a pharmaceutical medication [41], to January 31, 2022, based on PrEP-related keywords that were used in a previous study [42] and a new set of keywords related to injectable PrEP [31] (Textbox 1). To emulate how users might search for information about PrEP on the web, we started from the Twitter URLs and further assessed the contents of external URLs that were embedded in the original URL (ie, manual web crawling). For each URL visited, PrEP information was manually and systematically collected (eg, text about PrEP) from the URLs. We repeated this process until all the relevant information linked to the original URLs and their referred external URLs were collected.

**Pre-exposure prophylaxis (PrEP)–related keywords for tweet collection.**

**PrEP keywords**: [(truvada) OR (hiv AND PrEP) OR (preexposure AND prophylaxis AND hiv) OR (pill AND prevent AND hiv) OR (pill AND protect AND hiv) OR (pill AND protect AND AIDS)] [42]

**Injectable PrEP keywords**: [(Apretude) OR (cabotegravir) OR (inject AND prevent AND hiv) OR (inject AND protect AND hiv)]

#### Collecting Actively Obtained Information

To assess actively obtained information, a systematic search of PrEP was conducted using 5 popular search engines (ie, Google, Yahoo, Bing, DuckDuckGo, and Ask) from April to June 2022. We simulated the behavior of general consumers using the following various search terms and phrases: “PrEP,” “PrEP information,” “What is PrEP,” “How to use PrEP,” “Pre-exposure Prophylaxis frequently asked questions,” “how does PrEP work in HIV prevention,” “PrEP brochure,” “PrEP facts,” “HIV and PrEP,” “Truvada PrEP,” “PrEP side effects,” “how much does PrEP cost,” or “how to take prep” until a search result saturation was reached (ie, new information was not obtained). Then for comparison purposes, we specifically searched for “medication guide,” “patient information,” “info for people,” “information for the patient,” “information for the user,” and “patient version” of known PrEP medications, (eg, Truvada, Descovy, Apretude, Vocabria, and cabotegravir)*.*

For each of the 5 search engines, search results were collected from the first page for each search engine because most users rarely read past the first page of search results [43] and because our focus was to analyze the most frequently accessed web-based PrEP information. We did not include sponsored listings, as we wanted to focus on the most relevant and unbiased information. We manually verified search results to ensure that we only included web pages with PrEP information. We then systematically and manually web crawled these pages, using links from the search results as a starting point to emulate user behavior. Several false-positive links were excluded from the search results that were related to different acronyms or abbreviations (eg, Professional Research Experience Program). We also excluded documents without any PrEP-related information (eg, content about drugs used only for HIV treatment). We also excluded papers published in peer-reviewed journals that are not intended for the general public and would skew the readability results. Several documents were part of a much bigger document (eg, product monograph and prescribing information). Despite their relevance, if the overall PrEP information document exceeded 32,767 characters, we only included sections on PrEP that were written for the prescription drug prescribers in our analyses. We felt that this would most closely emulate how lay people would read this text. Duplicate sources obtained from more than one search path, educational videos, and descriptive figures and images were excluded and removed, and the web pages were converted to plain text for analysis.

### Ethical Considerations

We only analyzed publicly available documents in this study and did not analyze identifiable private information or involve any direct or indirect interactions with individuals. Per UNC Charlotte’s policy (citation 45 CFR 46 Definitions), the study is exempt from institutional review board requirements because it does not meet the regulatory definitions of human subject research.

### Data Categorization

#### Information Source

We categorized information sources according to the originator of the document. The originator type was first determined with manual Google searches about organizations. This information was supplemented with information from the “about us” section of websites. We also used information in the web-based sources about affiliations, funding sources, and the Crunchbase [44] to finalize their organization types. Several websites still had no explicit indication of their affiliations or funding sources; we have denoted them as “N/A” and categorized them separately in our comparison analyses.

The information sources for this study included national and state governmental agencies (ie, US-located government entities; eg, CDC and California Department of Public Health), non-US governmental agencies (eg, National Health Service of the United Kingdom), other public health organizations (eg, WHO and Joint United Nations Programme on HIV/AIDS), nongovernment organizations (eg, Mayo Clinic), and for-profit organizations (eg, Gilead Sciences).

#### Document Format and Communication Method

Communication methods were categorized as brochures, information sheets, or websites by manually assessing each document. To identify brochures, we first searched for the keyword “brochure” in the URLs, and then, we manually verified other documents with similar presentations of information. Brochures were distinguished from information sheets by their inclusion of images, figures, or pictures. To identify the information sheets, we searched for the keywords “factsheet” and “sheet” from the remaining URLs. Then, we manually labeled other information sheets by searching for similar presentations of information, which contained only text (ie, no images, figures, and pictorial descriptions). Other resources were categorized as websites because they were hosted on a web page and did not meet the criteria for either brochure or information sheet.

#### PrEP Modality

All documents were manually categorized based on PrEP modality (ie, delivery methods) to allow comparison by modality. We classified documents with one or more keywords—“oral,” “tablet,” “pill,” “Truvada,” “Descovy,” “daily PrEP,” “PrEP 2-1-1,” and “PrEP on demand”—in the title as “oral PrEP.” We classified documents with the keywords “Cabotegravir,” “Apretude,” “long-acting,” “injection PrEP,” and “injectable” as “injectable PrEP.” If a document had both types of keywords in the title, we categorized it as “oral and injectable PrEP.” For documents without these keywords in the title, the modality was categorized based on the content of the document. Documents without PrEP modality descriptions were categorized as general PrEP.

#### Intended Audience

The intended audience was manually determined for each document, that is, patient, provider, or general public. We used document titles and the first paragraph to determine the target audience. To identify documents written for patients, we looked for the following titles: “Patient Information,” “Medication Guide,” “Patient Medication Information,” “Information for the user,” “Information for the patient,” and “Overview for Patients.” Then, we read the first paragraph to ensure its intended audience was patient and checked if it had statements similar to the following. “Read this carefully before you start taking [Drug name] and each time you get a refill. […].” Similarly, documents written for providers were identified if they had these or similar titles: “Clinical Guide,” “Provider Frequently Asked Questions,” “Provider FAQs,” “Clinician Guidance,” and “Guide for Medical Providers.” We also read the first paragraph to check phrases similar to “Review this guide when prescribing [Drug name].” The intended audience for documents not meeting the criteria for patient or provider documents was determined as intended for the general public.

#### Measuring Readability

We used the Flesch-Kincaid grade level [45], Simple Measure of Gobbledygook Index [46], Coleman and Liau Index [47], and Automated Readability Index [48] to calculate the text readability, which is defined here as the grade level required to comprehend the text in the US education system. These indices are widely used in previous readability studies [23,24,26,49-53], and we applied them to the entire data set. To computationally perform readability analysis, the readability scores were first calculated based on the 4 readability indices using the open-source Python *textstat* package [54]. Given that different readability indices can generate a range of results, we used the mean of the 4 readability indices in our analyses to increase the reliability of our results but also report results generated by all 4 indices (Multimedia Appendix 1). We then conducted 5 sets of pairwise independent sample *t* tests to compare readability scores based on the method of obtaining the information, information source, document format and communication method, PrEP modality, and intended audience. When necessary, *P* values were adjusted using the prespecified Hommel procedure [55] to account for multiple comparisons within each set.

## Results

### Information Obtained Method

We collected a total of 463 documents, 194 from Twitter (ie, passively obtained information) and 269 from search engines (ie, actively obtained information). A total of 17 documents were duplicates. For analyses, we removed 1 set of the 17 duplicate documents. The documents were excluded in both sets, a total of 34 documents, when comparing the readability of documents based on methods for obtaining information. Using the average of the 4 reading level indices, the overall average reading level for web-based PrEP materials was a 10.20 grade level with an SE of 0.11. This is significantly above the recommended grade level of lower than ninth grade for the comprehension of health materials. The readability scores of materials retrieved from Twitter (9.8 grade level, SE 0.16) and search engines (10.5 grade level, SE 0.15) were significantly different (*P*=.002 by independent sample *t* tests).

**Figure 1:**
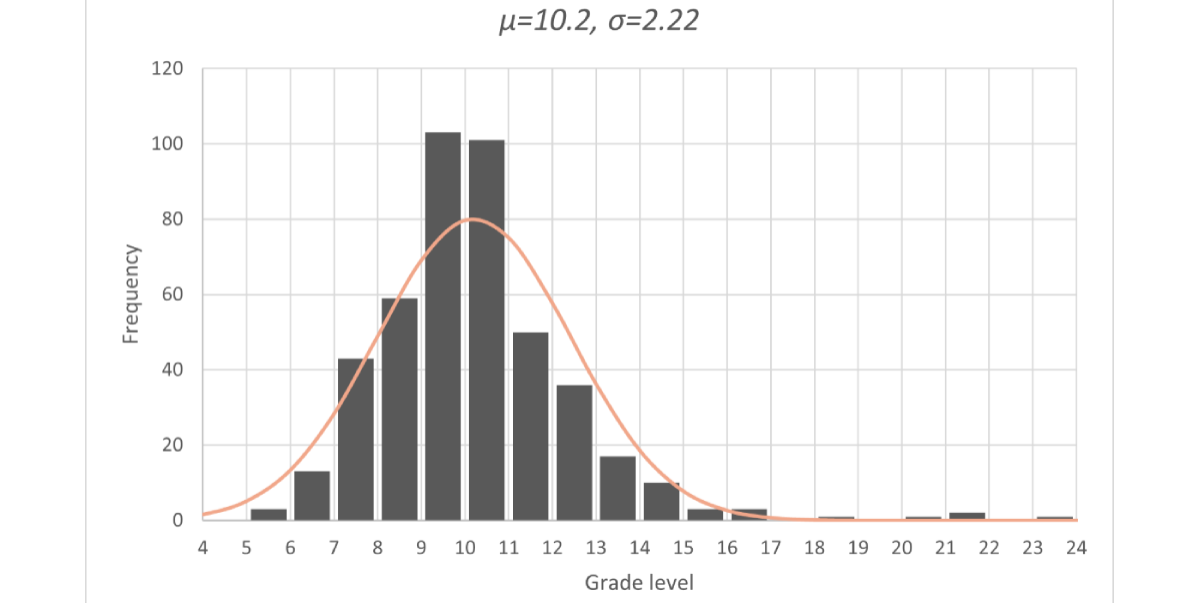
The empirical distribution of the average readability level required for understanding web-based pre-exposure prophylaxis information.

We found that the distribution of readability scores for all documents was approximately normally distributed (ie, unimodal) with a slight skew to the right and a mode between the ninth and eleventh reading levels as shown in Figure 1. About 74% (n=328) of web-based sources required reading levels higher than ninth-grade.

### Information Source

We found 172 documents from 45 for-profit entities (eg, Gilead Sciences and Nurx), 157 documents from 49 nongovernment organizations (eg, Mayo Clinic and Black AIDS Institute), 88 documents from 28 US governmental entities (eg, CDC and NIH), and 16 documents from 7 non-US governmental entities (eg, National Health Service of the United Kingdom and Health Service Executive-Ireland) or other public health organizations (eg, WHO and Joint United Nations Programme on HIV/AIDS). A total of 13 documents from organizations had no explicit indication of their affiliations or funding sources; they were excluded from our information source comparison analyses.

Some organizations and entities developed multiple PrEP-related documents, but they differed in content and topic. For example, some documents provided details about a particular PrEP drug (eg, Truvada and Descovy), while others provided information about accessing PrEP or PrEP cost. Still, others were different document types (eg, brochures). In our analyses, we treated each document as a separate data point.

Complete readability scores for each document are presented in Multimedia Appendix 1. The overall average grade reading level required to understand web-based PrEP information (10.2 grade level, SE 0.1) is considerably higher than the target grade level recommended by health agencies for health information. The mean grade reading levels and SEs (ie, of the 4 indices) from these organizations are displayed below and compared in Table 1. There was no significant difference in readability among organization types.

- Non-US government entities or other public health organizations: mean 10.6 (SE 0.7)
- For-profit entities: mean 10.4 (SE 0.2)
- The US-located government entities: mean 10.3 (SE 0.2)
- Nongovernment organizations: mean 9.9 (SE 0.2)

**Table 1:** Pairwise 2 sample *t* test of the average readability by source type.

Source type	*P* value	Adjusted *P* value
Non-US government entities or other public health organizations^a^	.22	.66
For-profit entities^a^	.04	.12
The US-located government entities^a^	.15	.46
Non-US government entities or other public health organizations^b^	.65	.74
For-profit entities^b^	.75	.75
Non-US government entities or other public health organizations^c^	.74	.74

^a^Versus nongovernment organizations.

^b^Versus the US-located government entities.

^c^Versus for-profit entities.

### Document Format and Communication Method

The most common type of document was websites (n=384), including content from health organizations and health care professionals. Websites typically provide a comprehensive range of information on multiple related topics, such as HIV/AIDS and PrEP access, at a reading level of 10.2 grade (SE 0.46).

In our data set, information sheets (n=29) were in PDF file format and had titles containing phrases such as “Patient Medication,” “Medication Guide,” “Information for the user,” “Information sheet,” “Fact sheet,” “Facts,” and “Information booklet.” Compared to other document formats, they were also lengthy and detailed, including medication information and instructions for patient use. Most medication guides were written by the drug manufacturers (eg, Gilead Sciences and ViiV Healthcare) and FDA. Five information sheets were provided by nonprofit and non-US government health organizations. Information sheets were written at a reading level of 11.2 grade (SE 0.46).

**Figure 2:**
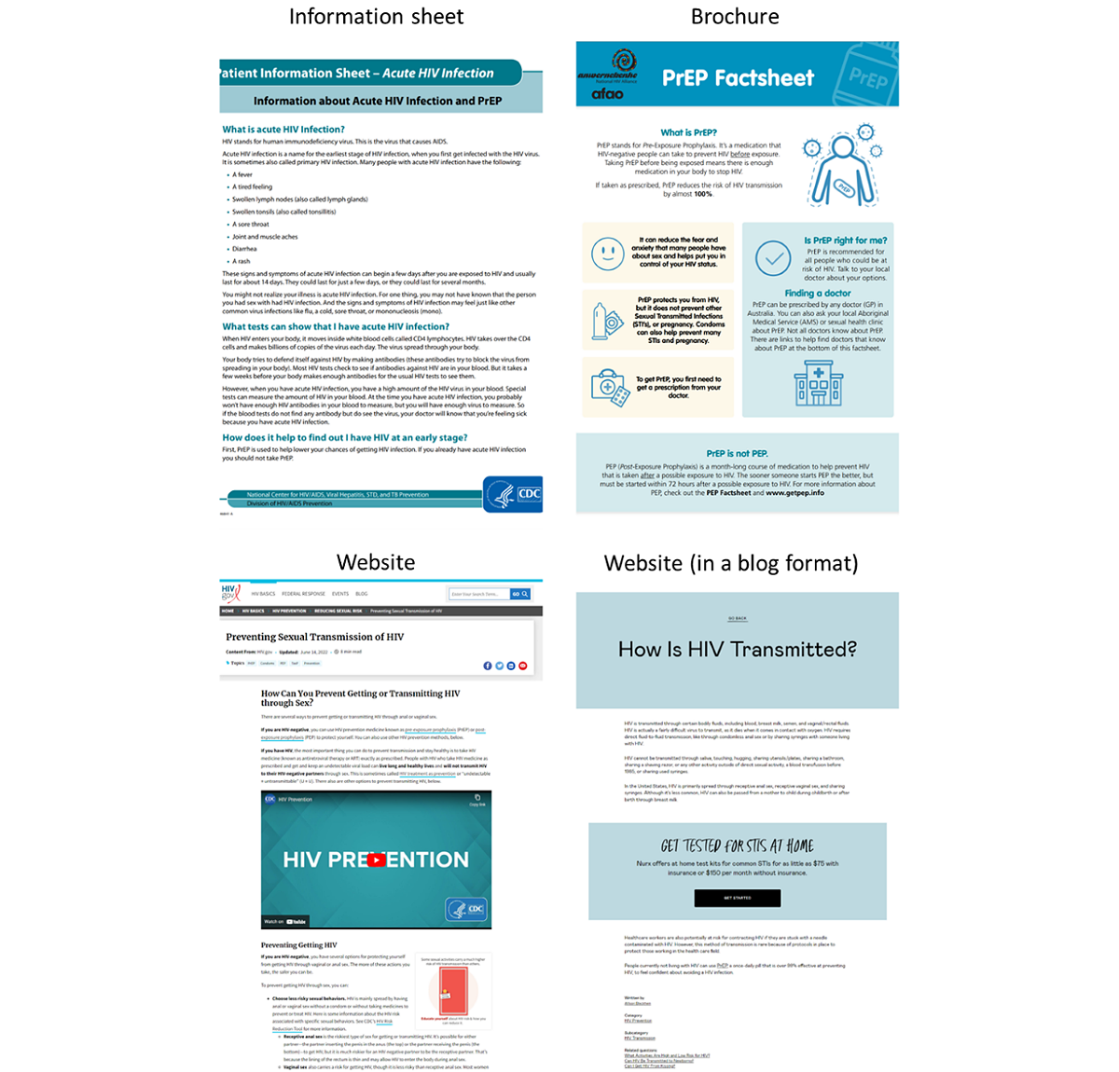
Examples of document types used to disseminate pre-exposure prophylaxis (PrEP) information on the web: information sheet (top left), brochure (top right), website (bottom left), and website in a blog format (bottom right). More information about each document, including the source URL, can be found in Multimedia Appendix 1, rows 12, 272, 114, and 147. For a higher-resolution version of this figure, see Multimedia Appendix 2.

Examples of each type of document are provided in Figure 2. Brochures (n=33) included user guides and leaflets containing commonly asked questions such as “What is PrEP” and “How to get PrEP.” Brochures were prepared by federal and state governmental organizations (n=15), non-US governmental organizations (n=2), nongovernmental organizations (n=10), and for-profit organizations (n=6). The 3 document types had significantly different readability levels: the readability of brochures was easier than both information sheets (adjusted *P*<.001) and websites (adjusted *P*=.003), with an average readability level at the recommended ninth grade level (SE 0.31).

### PrEP Modality

We identified 220 documents relating to oral PrEP only, 14 documents relating to injectable PrEP only, and 14 documents mentioning both oral and injectable PrEP. The rest of the documents (n=198, 44%) did not mention a specific PrEP delivery method (ie, “general PrEP”); these latter documents were excluded from analyses. On average, the content on injectable PrEP (12.7 grade level, SE 0.9) was more difficult to comprehend than the content on oral PrEP (10.2 grade level, SE 0.2). Information on both forms of PrEP was intermediate in readability (10.8 grade level, SE 0.6). The comparison analysis results are shown in Table 2.

**Table 2:** Pairwise 2 sample *t* test of the average readability by PrEP^a^ modality.

PrEP modality	*P* value	Adjusted *P* value
Injectable PrEP^b^	<.001	<.001
Oral and injectable PrEP^b^	.30	.30
Injectable PrEP^c^	.09	.09

^a^PrEP: pre-exposure prophylaxis.

^b^Versus oral PrEP.

^c^Versus oral and injectable PrEP.

### Intended Audience

Most documents (n=426, 95.5%) were categorized as “general public” because they did not specify their intended audience. We found 14 documents specifically written for PrEP users and 6 specifically written for providers.

The readability grade level indices of content intended for PrEP providers (15.6 grade level, SE 1.59) were significantly higher when compared to the content intended for patients (10.2 grade level, SE 0.3; adjusted *P*<.001) and the general public (10.1 grade level, SE 0.1; adjusted *P*<.001). The reading level of content written for PrEP users was similar to that of materials for the general public (adjusted *P*=.84).

## Discussion

### Principal Findings

Our study highlights the challenging task of effectively educating diverse audiences about PrEP. Our results are consistent with previous work [25], which found that web-based PrEP information is often difficult to understand. We add to this previous work by including data disseminated in social media and stratifying web-based PrEP documents by how the PrEP document was obtained on the web, information source, document format and communication method, PrEP modality, and intended audience. We found PrEP materials from Twitter were significantly easier to comprehend than those identified by web searches, despite the fact Twitter was merely posting URLs of web-based information. It is possible that organizations selectively identify more comprehendible materials to promote through Twitter. The readability of web-based PrEP information from different organizational groups was all higher than recommended, and there was no significant difference between them (Table 1). This contrasts with a previous study that focused on different health conditions [26]. Informational documents intended for providers had significantly higher reading levels than those intended for patients or the general public. However, the materials intended for the patients and the public were still higher than the recommended ninth-grade reading level.

Brochures were the only type of PrEP materials that achieved the HHS and NIH target of ninth-grade reading level [13]. Brochures have been used in successful public health campaigns for facilitating behavior change, knowledge increase, and self-efficacy for other health conditions [56]. Brochures may be effective means of PrEP communication for most people who are not medical providers. However, brochures were not a common mode of conveying information: there were only 33 brochures in 446 unique documents that we identified.

As PrEP regimens become more complex (eg, injectable PrEP and 2-1-1 PrEP), educational materials might become more difficult to comprehend. For example, we identified that a higher reading level was required to understand materials about injectable PrEP. Yet, there is great interest in injectable PrEP: in a National HIV Behavioral survey of 314 people, injectable PrEP was preferred by 3 times more respondents compared to oral PrEP [31]. This calls for attention to improving the readability of materials describing injectable PrEP because difficulty in understanding injectable PrEP information could discourage people who would otherwise be interested in the new PrEP route of administration.

Our findings suggest that there is a need for health communication strategies and policies that support the development and dissemination of clear and concise PrEP information. Our finding suggests that health communication strategies can be enhanced by using brochures and plain language, which may include avoiding jargon. Policies can also be improved to support this effort. For instance, governments could increase funding for research into PrEP literacy and the development of PrEP communication guidelines. Additionally, governments could mandate that health care providers receive training on how to communicate about PrEP in a clear and easy-to-understand way for a variety of audiences. Together, these communication strategies and policies can help individuals to have access to clear and easy-to-understand information about PrEP, which can help to increase PrEP uptake and reduce HIV transmission.

### Limitations

Our findings should be understood in the light of their limitations. First, individuals accessing PrEP information on the web may be a select subgroup of those interested in PrEP and might have higher or lower health literacy than those who seek information from other sources. However, given that the internet has become an increasingly popular resource for health information [32,57] and groups potentially eligible for PrEP [58] tend to have high internet use [59], it is likely that a substantial proportion of potential and current PrEP users would seek PrEP information on the web. Second, current general-purpose readability indices alone may not be a perfect measurement of comprehension and reading level [60,61], especially for assessing documents with nontext content, such as charts, graphs, and videos. For instance, we noted that all brochures in our data set contained images. Pictorial and graphic representations of information have been shown to be more effective than text-only messages [62]. Similarly, we found that some websites also contain PrEP-related informational videos; videos have been shown to be effective in modifying health behaviors, including promoting HIV testing [63]. In this study, we focused on textual information as text remains the primary medium for health communication on the web [64] and relied on the average scores of 4 readability indices to minimize the lack of sensitivity or breadth of measurements from using a single metric. Third, our analysis, although systematic, was not exhaustive. The web-based PrEP information included in our study was collected from a variety of sources, yet it is possible that some important information was missed. We limited our search to English language materials, and the first page of the search result imitated users’ web-based behavior [43]. Lastly, we did not use generic names of PrEP medications, thus potentially not including PrEP information that only might use the generic names.

### Future Directions

Despite public guidelines for writing health education materials, readability levels of web-based health materials, even those written by public health organizations, have either become more difficult [65] or reflected no improvement [19] with few exceptions [66]. AMA guidelines suggest addressing health literacy concerns by avoiding unnecessary details and lengthy background information [15]. This recommendation is consistent with our finding that documents in the brochure format, which were assessed as most readable, were concise, focused more on patient needs, and provided less background information. Readability is only one measure of the impact of health information, for example, measures of health literacy are evolving to include measures of the extent to which materials increase decision-making ability [67]. Thus, future research on health literacy should extend to whether educational materials are helpful to clients in making informed decisions.

Social media platforms have been widely used for public health communication and education, but research focused on measuring the effectiveness of social media as a channel for public health information is limited (with a few notable exceptions [68]). A better understanding of social media platforms’ effectiveness should improve public health communication and the development of an impactful campaign for facilitating behavior change, knowledge increase, and self-efficacy related to public health needs—including PrEP uptake. Inequitable PrEP uptake persists among racial, ethnic, and gender minority communities [6,7]. Follow-up studies should (1) optimize the effectiveness of web-based educational materials in making informed decisions among health equity populations and (2) tailor messaging to address the information needs of specific populations to address inequitable PrEP uptake.

Future studies of the readability of web-based PrEP information should consider the following points. PrEP use and HIV prevention are global concerns, and it is important to understand how people in other languages are accessing and understanding information about PrEP. Future studies should examine materials in other languages. To provide a more comprehensive picture of the range of available web-based PrEP information, future work can expand the data collection process by expanding the number of result pages, search keywords, search engines, and search locations and by applying various filters to narrow and rank the search results. In addition, future work should consider exploring other measures of comprehension, especially for materials that include images, videos, and other nontext elements. We believe that these measures will strengthen the findings and provide a more comprehensive picture of how people are understanding information about PrEP.

### Conclusions

The overall reading level of PrEP information found on the web was above that recommended for most potential users; if unaddressed, other challenges to supporting PrEP use by those most likely to benefit might be made more difficult because of a gap in comprehension of web-based information. The readability of web-based PrEP information needs to be improved to comply with health communication recommendations, reduce the barrier of a health literacy gap, facilitate informed decisions by those with a need for PrEP, and counter the HIV epidemic.

## Appendix

Multimedia Appendix 1 The readability scores for each document collected from search engine and Twitter.

Multimedia Appendix 2 Examples of document types used to disseminate pre-exposure prophylaxis (PrEP) information on the web.

## Abbreviations

AMA: American Medical Association
CDC: Centers for Disease Control and Prevention
FAQ: frequently asked question
FDA: US Food and Drug Administration
HHS: US Department of Health and Human Services
HSE: Health and Safety Executive
NIH: National Institutes of Health
PrEP: pre-exposure prophylaxis
WHO: World Health Organization

## Data Availability

The data sets generated during and analyzed during this study are available from the corresponding author on reasonable request.

## References

[1] Choopanya K, Martin M, Suntharasamai P, Sangkum U, Mock PA, Leethochawalit M, Chiamwongpaet S, Kitisin P, Natrujirote P, Kittimunkong S, Chuachoowong R, Gvetadze RJ, McNicholl JM, Paxton LA, Curlin ME, Hendrix CW, Vanichseni S, Bangkok Tenofovir Study Group (2013). Antiretroviral prophylaxis for HIV infection in injecting drug users in Bangkok, Thailand (the Bangkok Tenofovir Study): a randomised, double-blind, placebo-controlled phase 3 trial. Lancet.

[2] Haberer JE, Baeten JM, Campbell J, Wangisi J, Katabira E, Ronald A, Tumwesigye E, Psaros C, Safren SA, Ware NC, Thomas KK, Donnell D, Krows M, Kidoguchi L, Celum C, Bangsberg DR (2013). Adherence to antiretroviral prophylaxis for HIV prevention: a substudy cohort within a clinical trial of serodiscordant couples in East Africa. PLoS Med.

[3] Smith DK, Herbst JH, Rose CE (2015). Estimating HIV protective effects of method adherence with combinations of preexposure prophylaxis and condom use among African American men who have sex with men. Sex Transm Dis.

[4] Fauci AS, Redfield RR, Sigounas G, Weahkee MD, Giroir BP (2019). Ending the HIV epidemic: a plan for the United States. JAMA.

[5] Sullivan PS, Giler RM, Mouhanna F, Pembleton ES, Guest JL, Jones J, Castel AD, Yeung H, Kramer M, McCallister S, Siegler AJ (2018). Trends in the use of oral emtricitabine/tenofovir disoproxil fumarate for pre-exposure prophylaxis against HIV infection, United States, 2012-2017. Ann Epidemiol.

[6] Siegler AJ, Mouhanna F, Giler RM, Weiss K, Pembleton E, Guest J, Jones J, Castel A, Yeung H, Kramer M, McCallister S, Sullivan PS (2018). The prevalence of pre-exposure prophylaxis use and the pre-exposure prophylaxis-to-need ratio in the fourth quarter of 2017, United States. Ann Epidemiol.

[7] Frieden TR, Foti KE, Mermin J (2015). Applying public health principles to the HIV epidemic—how are we doing?. N Engl J Med.

[8] Sullivan PS, Siegler AJ (2022). What will it take to meet UNAIDS targets for preexposure prophylaxis users?. Curr Opin Infect Dis.

[9] Sullivan PS, Mena L, Elopre L, Siegler AJ (2019). Implementation strategies to increase PrEP uptake in the south. Curr HIV/AIDS Rep.

[10] Lopez C, Kim B, Sacks K (2022). Health literacy in the United States: enhancing assessments and reducing disparities. Milken Institute.

[11] (2020). Healthy people 2020. US Department of Health and Human Services. Office of Disease Prevention and Health Promotion.

[12] Walsh TM, Volsko TA (2008). Readability assessment of internet-based consumer health information. Respir Care.

[13] Arkin EB (2009). Making health communication programs work. U.S. Department of Health & Human Services, National Institutes of Health, National Cancer Institute.

[14] (2015). How to write easy-to-read health materials. National Institutes of Health.

[15] Weiss B (2003). Health literacy. American Medical Association Foundation and American Medical Association.

[16] Fischhoff B, Brewer NT, Downs JS (2011). Communicating risks and benefits: an evidence-based user's guide. US Department of Health and Human Services, Food and Drug Administration (FDA).

[17] Skierkowski DD, Florin P, Harlow LL, Machan J, Ye Y (2019). A readability analysis of online mental health resources. Am Psychol.

[18] Lipari M, Berlie H, Saleh Y, Hang P, Moser L (2019). Understandability, actionability, and readability of online patient education materials about diabetes mellitus. Am J Health Syst Pharm.

[19] Sabharwal S, Badarudeen S, Unes Kunju S (2008). Readability of online patient education materials from the AAOS web site. Clin Orthop Relat Res.

[20] MacLean SA, Basch CH, Ethan D, Garcia P (2019). Readability of online information about HPV immunization. Hum Vaccin Immunother.

[21] Ayyaswami V, Padmanabhan D, Patel M, Prabhu AV, Hansberry DR, Agarwal N, Magnani JW (2019). A readability analysis of online cardiovascular disease-related health education materials. Health Lit Res Pract.

[22] Park A, Zhu SH, Conway M (2017). The readability of electronic cigarette health information and advice: a quantitative analysis of web-based information. JMIR Public Health Surveill.

[23] D'Alessandro DM, Kingsley P, Johnson-West J (2001). The readability of pediatric patient education materials on the World Wide Web. Arch Pediatr Adolesc Med.

[24] Agarwal N, Hansberry DR, Sabourin V, Tomei KL, Prestigiacomo CJ (2013). A comparative analysis of the quality of patient education materials from medical specialties. JAMA Intern Med.

[25] Kecojevic A, Basch CH, Garcia P (2020). Readability analysis of online health information on preexposure prophylaxis (PrEP). Public Health.

[26] Cochrane ZR, Gregory P, Wilson A (2012). Readability of consumer health information on the internet: a comparison of U.S. government-funded and commercially funded websites. J Health Commun.

[27] Keselman A, Smith CA (2012). A classification of errors in lay comprehension of medical documents. J Biomed Inform.

[28] Smith CA, Hetzel S, Dalrymple P, Keselman A (2011). Beyond readability: investigating coherence of clinical text for consumers. J Med Internet Res.

[29] Gombe MM, Cakouros BE, Ncube G, Zwangobani N, Mareke P, Mkwamba A, Prescott MR, Bhatasara T, Murwira M, Mangwiro AZ, Prust ML (2020). Key barriers and enablers associated with uptake and continuation of oral pre-exposure prophylaxis (PrEP) in the public sector in Zimbabwe: qualitative perspectives of general population clients at high risk for HIV. PLoS One.

[30] Muhumuza R, Ssemata AS, Kakande A, Ahmed N, Atujuna M, Nomvuyo M, Bekker LG, Dietrich JJ, Tshabalala G, Hornschuh S, Maluadzi M, Chibanda-Stranix L, Nematadzira T, Weiss HA, Nash S, Fox J, Seeley J (2021). Exploring perceived barriers and facilitators of PrEP uptake among young people in Uganda, Zimbabwe, and South Africa. Arch Sex Behav.

[31] Levy ME, Patrick R, Gamble J, Rawls A, Opoku J, Magnus M, Kharfen M, Greenberg AE, Kuo I (2017). Willingness of community-recruited men who have sex with men in Washington, DC to use long-acting injectable HIV pre-exposure prophylaxis. PLoS One.

[32] Fox S (2011). Health topics: 80% of internet users look for health information online. Pew Research Center’s Internet & American Life Project.

[33] Wilson SL, Wiysonge C (2020). Social media and vaccine hesitancy. BMJ Glob Health.

[34] Auxier B, Anderson M (2021). Social media use in 2021. Pew Research Center.

[35] Lober WB, Flowers JL (2011). Consumer empowerment in health care amid the internet and social media. Semin Oncol Nurs.

[36] Thackeray R, Neiger BL, Smith AK, Van Wagenen SB (2012). Adoption and use of social media among public health departments. BMC Public Health.

[37] Gatewood J, Monks SL, Singletary CR, Vidrascu E, Moore JB (2020). Social media in public health: strategies to distill, package, and disseminate public health research. J Public Health Manag Pract.

[38] Harris JK, Hawkins JB, Nguyen L, Nsoesie EO, Tuli G, Mansour R, Brownstein JS (2017). Using Twitter to identify and respond to food poisoning: the food safety STL project. J Public Health Manag Pract.

[39] Perrin A, Anderson M (2019). Share of U.S. adults using social media, including Facebook, is mostly unchanged since 2018. Pew Research Center.

[40] Roesslein J (2009). Tweepy.

[41] Gilead Sciences, Inc. (2012). U.S. Food and Drug Administration approves Gilead’s Truvada® for reducing the risk of acquiring HIV: first agent indicated for uninfected adults at high risk of acquiring HIV through Sex. Business Wire.

[42] Kakalou C, Lazarus JV, Koutkias V (2019). Mining social media for perceptions and trends on HIV pre-exposure prophylaxis. Stud Health Technol Inform.

[43] van Deursen AJAM, van Dijk JAGM (2009). Using the internet: skill related problems in users' online behavior. Interact Comput.

[44] Crunchbase.

[45] Kincaid JP, Fishburne RP, Rogers RL, Chissom BS (1975). Derivation of new readability formulas (automated readability index, fog count, and Flesch reading ease formula) for navy enlisted personnel. Institute for Simulation and Training.

[46] McLaughlin GH (1969). SMOG grading—a new readability formula. J Read.

[47] Coleman M, Liau TL (1975). A computer readability formula designed for machine scoring. J Appl Psychol.

[48] Smith EA, Senter RJ (1967). Automated readability index. AMRL TR.

[49] Meade CD, Byrd JC (1989). Patient literacy and the readability of smoking education literature. Am J Public Health.

[50] Malouff J, Gabrilowitz D, Schutte N (1992). Readability of health warnings on alcohol and tobacco products. Am J Public Health.

[51] Tian C, Champlin S, Mackert M, Lazard A, Agrawal D (2014). Readability, suitability, and health content assessment of web-based patient education materials on colorectal cancer screening. Gastrointest Endosc.

[52] Yin HS, Gupta RS, Tomopoulos S, Wolf MS, Mendelsohn AL, Antler L, Sanchez DC, Lau CH, Dreyer BP (2013). Readability, suitability, and characteristics of asthma action plans: examination of factors that may impair understanding. Pediatrics.

[53] Terblanche M, Burgess L (2010). Examining the readability of patient-informed consent forms. Open Access J Clin Trials.

[54] Bansal S, Aggarwal C (2015). textstat. MIT.

[55] Wright SP (1992). Adjusted P-values for simultaneous inference. Biometrics.

[56] Anker AE, Feeley TH, McCracken B, Lagoe CA (2016). Measuring the effectiveness of mass-mediated health campaigns through meta-analysis. J Health Commun.

[57] Fox S, Duggan M (2013). Health online 2013: one in three American adults have gone online to figure out a medical condition. Pew Research Center.

[58] U.S. Centers for Disease Control and Prevention (2021). Core indicators for monitoring the ending the HIV epidemic initiative (early release). HIV Surveillance Data Tables.

[59] Perrin A, ATSKE S (2021). About three-in-ten U.S. adults say they are ‘almost constantly’ online. Pew Research Center.

[60] Meade CD, Smith CF (1991). Readability formulas: cautions and criteria. Patient Educ Couns.

[61] Davison A, Kantor RN (1982). On the failure of readability formulas to define readable texts: a case study from adaptations. Read Res Q.

[62] Hammond D (2011). Health warning messages on tobacco products: a review. Tob Control.

[63] Tuong W, Larsen ER, Armstrong AW (2014). Videos to influence: a systematic review of effectiveness of video-based education in modifying health behaviors. J Behav Med.

[64] Himelboim I, McCreery S (2012). New technology, old practices: examining news websites from a professional perspective. Convergence.

[65] Zhang D, Earp BE, Kilgallen EE, Blazar P (2022). Readability of online hand surgery patient educational materials: evaluating the trend since 2008. J Hand Surg Am.

[66] Wong K, Levi JR (2017). Readability trends of online information by the American Academy of Otolaryngology-Head and Neck Surgery Foundation. Otolaryngol Head Neck Surg.

[67] Healthy people 2010 final review. U.S. Department of Health and Human Services. Centers for Disease Control and Prevention.

[68] Al-Dmour H, Masa'deh R, Salman A, Abuhashesh M, Al-Dmour R (2020). Influence of social media platforms on public health protection against the COVID-19 pandemic via the mediating effects of public health awareness and behavioral changes: integrated model. J Med Internet Res.

