# Supplementary material for: Health Information on Pre-Exposure Prophylaxis From Search Engines and Twitter: Readability Analysis
Source: JMIR Public Health Surveill. 2023 Sep 4;9:e48630. doi: 10.2196/48630 (PMC10507523; doi:10.2196/48630)
Supplement: Multimedia Appendix 1 [file publichealth_v9i1e48630_app1.docx]

**Multimedia Appendix 1.** The readability scores for each document collected from search engine and Twitter.

| ID | Organization | Organization Type | flesch_kincaid_grade | smog_index | coleman_liau_index | automated_readability_index | Average | Original URL | Archived URL | Note | Information Source | PrEP Modality | Intended Audience | Document Format |
| --- | --- | --- | --- | --- | --- | --- | --- | --- | --- | --- | --- | --- | --- | --- |
| 1 | FDA - U.S. Food and Drug Administration | The US Government | 7.7 | 11.2 | 10.78 | 9.8 | 9.87 | https://www.accessdata.fda.gov/drugsatfda_docs/label/2006/021752s005lbl.pdf | N/A | Truvada (Page 26) | Search Engine | Oral | Patient | Information Sheet |
| 2 | FDA - U.S. Food and Drug Administration | The US Government | 8.4 | 11.3 | 10.84 | 10.5 | 10.26 | https://www.accessdata.fda.gov/drugsatfda_docs/label/2012/021752s030mg.pdf | N/A | Truvada (Main page / Page 39) | Search Engine | Oral | Patient | Information Sheet |
| 3 | FDA - U.S. Food and Drug Administration | The US Government | 6.7 | 9.9 | 11.24 | 10.3 | 9.535 | https://www.accessdata.fda.gov/drugsatfda_docs/label/2017/208215s005lbl.pdf | https://web.archive.org/web/20220610172226/https://www.accessdata.fda.gov/drugsatfda_docs/label/2017/208215s005lbl.pdf | Descovy (Page 28) | Search Engine | Oral | Patient | Information Sheet |
| 4 | FDA - U.S. Food and Drug Administration | The US Government | 9.1 | 11.1 | 10.96 | 11.4 | 10.64 | https://www.accessdata.fda.gov/drugsatfda_docs/label/2021/215499s000lbl.pdf | N/A | Apretude (After page no. 35) | Search Engine | Injection | Patient | Information Sheet |
| 5 | Gilead Sciences | For Profit | 8.5 | 10.7 | 11.83 | 11.6 | 10.6575 | https://pdf.hres.ca/dpd_pm/00053247.PDF | N/A | Descovy (Page 57) | Search Engine | Oral | Patient | Information Sheet |
| 6 | Gilead Sciences | For Profit | 9.4 | 11.2 | 11.26 | 11.7 | 10.89 | https://www.ema.europa.eu/en/documents/product-information/descovy-epar-product-information_en.pdf | https://web.archive.org/web/20220610174140/https://www.ema.europa.eu/en/documents/product-information/descovy-epar-product-information_en.pdf | Descovy (Page 71) | Search Engine | Oral | Patient | Information Sheet |
| 7 | ViiV-Healthcare | For Profit | 9.4 | 12.1 | 12.24 | 12.6 | 11.585 | https://www.ema.europa.eu/en/documents/product-information/vocabria-epar-product-information_en.pdf | https://web.archive.org/web/20220610174140/https://www.ema.europa.eu/en/documents/product-information/vocabria-epar-product-information_en.pdf | Vocabria/Cabotegravir (Page 64) | Search Engine | Injection | Patient | Information Sheet |
| 8 | Gilead Sciences | For Profit | 8.3 | 11.2 | 10.9 | 10.3 | 10.175 | https://www.truvadahcp.com/. https://www.gilead.com/~/media/Files/pdfs/medicines/hiv/truvada/truvada_pi.pdf |  | Truvada PrEP (Page 35) | Search Engine | Oral | Patient | Information Sheet |
| 9 | National Institutes of Health - Office of AIDS Research | The US Government | 9.5 | 12.2 | 11.31 | 12 | 11.2525 | https://clinicalinfo.hiv.gov/en/drugs/emtricitabine-tenofovir-disoproxil-fumarate/patient | https://web.archive.org/web/20220515184551/https://clinicalinfo.hiv.gov/en/drugs/emtricitabine-tenofovir-disoproxil-fumarate/patient | Truvada PrEP | Search Engine | Oral | Patient | Website |
| 10 | NYC Health | The US Government | 5.6 | 9.2 | 7.71 | 7.5 | 7.5025 | https://www1.nyc.gov/assets/doh/downloads/pdf/ah/prep-user-guide.pdf | https://web.archive.org/web/20220516012216/https://www1.nyc.gov/assets/doh/downloads/pdf/ah/prep-user-guide.pdf | PrEP | Search Engine | Oral | Patient | Brochure |
| 11 | San Francisco AIDS Foundation | Non Government Organization | 7 | 9.8 | 8.07 | 8.9 | 8.4425 | https://www.sfaf.org/resource-library/tips-info-for-prep-users/ | https://web.archive.org/web/20220516020019/https://www.sfaf.org/resource-library/tips-info-for-prep-users/ | PrEP | Search Engine | Oral | Patient | Website |
| 12 | Centers for Disease Control and Prevention | The US Government | 7.7 | 10.9 | 8.76 | 10.4 | 9.44 | https://www.cdc.gov/hiv/pdf/prep_gl_patient_factsheet_acute_hiv_infection_english.pdf | N/A | HIV | Twitter | General PrEP | Patient | Information Sheet |
| 13 | U.S. Department of Veterans Affairs | The US Government | 11.4 | 13.7 | 10.62 | 13.6 | 12.33 | https://www.hiv.va.gov/pdf/prep-patient-overview.pdf | https://web.archive.org/web/20220420173819/https://www.hiv.va.gov/pdf/prep-patient-overview.pdf | PrEP | Both | Oral | Patient | Information Sheet |
| 14 | Centers for Disease Control and Prevention | The US Government | 8.9 | 12.5 | 10.73 | 10.8 | 10.7325 | https://www.cdc.gov/hiv/pdf/prep_gl_patient_factsheet_truvada_english.pdf | N/A | Truvada | Both | Oral | Patient | Information Sheet |
| 15 | aidsmap | Non Government Organization | 9 | 11.9 | 11.02 | 11.2 | 10.78 | https://www.aidsmap.com/about-hiv/pre-exposure-prophylaxis-prep | https://web.archive.org/web/20220425190938/https://www.aidsmap.com/about-hiv/pre-exposure-prophylaxis-prep | PrEP | Both | Oral & Injection PrEP | General | Website |
| 16 | aidsmap | Non Government Organization | 9.1 | 11.4 | 12.53 | 12.6 | 11.4075 | https://www.aidsmap.com/about-hiv/truvada-or-descovy-which-should-i-take-prep | https://web.archive.org/web/20220425190945/https://www.aidsmap.com/about-hiv/truvada-or-descovy-which-should-i-take-prep | PrEP | Both | Oral | General | Website |
| 17 | Centers for Disease Control and Prevention | The US Government | 5.1 | 8.6 | 8.17 | 7.1 | 7.2425 | https://www.cdc.gov/hiv/pdf/basics/prep/cdc-hiv-stsh-prep-brochure-english.pdf | N/A | PrEP | Both | Oral | General | Brochure |
| 18 | Centers for Disease Control and Prevention | The US Government | 7.7 | 9.9 | 8.41 | 8.9 | 8.7275 | https://www.cdc.gov/stophivtogether/library/topics/prevention/brochures/cdc-lsht-prevention-brochure-prep-is-for-women-patient.pdf?s_cid=so_prepwomenpdftwitter202108010001 | https://web.archive.org/web/20220425203635/https://www.cdc.gov/stophivtogether/library/topics/prevention/brochures/cdc-lsht-prevention-brochure-prep-is-for-women-patient.pdf?s_cid=so_prepwomenpdftwitter202108010001 | PrEP | Both | Oral | General | Brochure |
| 19 | Gilead Sciences | For Profit | 7 | 10.7 | 9.8 | 9.4 | 9.225 | https://www.truvada.com/?utm_medium=cpc&utm_campaign=71700000069298924&utm_content=Truvada_KW&utm_term=Truvada&utm_source=google&&gclid=CjwKCAjwr7X4BRA4EiwAUXjbt-XaXvqu2zrCZWOFXMDckBk5E6UudBt5UQ6tmbXuRXl58OUGDUV4TBoCdSIQAvD_BwE&gclsrc=aw.ds | N/A | HIV | Both | Oral | General | Website |
| 20 | Gilead Sciences | For Profit | 7 | 10.5 | 9.74 | 9.3 | 9.135 | https://www.truvada.com/what-is-truvada/understanding-truvada | N/A | Truvada | Both | Oral | General | Website |
| 21 | National Institutes of Health - Office of AIDS Research | The US Government | 8.1 | 10.3 | 8.94 | 9.6 | 9.235 | https://hivinfo.nih.gov/understanding-hiv/fact-sheets/pre-exposure-prophylaxis-prep | https://web.archive.org/web/20220420220647/https://hivinfo.nih.gov/understanding-hiv/fact-sheets/pre-exposure-prophylaxis-prep | HIV-AIDS, PrEP & PEP | Both | General PrEP | General | Website |
| 22 | NYC Health | The US Government | 7.4 | 9.4 | 8.23 | 8.4 | 8.3575 | https://www1.nyc.gov/site/doh/health/health-topics/pre-exposure-prophylaxis-prep.page | https://web.archive.org/web/20220420013410/https://www1.nyc.gov/site/doh/health/health-topics/pre-exposure-prophylaxis-prep.page | HIV & PrEP | Both | Oral | General | Website |
| 23 | San Francisco AIDS Foundation | Non Government Organization | 12 | 13.7 | 14.33 | 14.9 | 13.7325 | https://www.sfaf.org/collections/beta/prep-facts-how-much-does-prep-cost/ | https://web.archive.org/web/20220425224620/https://www.sfaf.org/collections/beta/prep-facts-how-much-does-prep-cost/ | PrEP | Both | General PrEP | General | Website |
| 24 | San Francisco AIDS Foundation | Non Government Organization | 8.2 | 11 | 10.15 | 9.5 | 9.7125 | https://www.sfaf.org/collections/beta/prep-facts-introduction-faq/ | https://web.archive.org/web/20220419221605/https://www.sfaf.org/collections/beta/prep-facts-introduction-faq/ | PrEP | Both | Oral | General | Website |
| 25 | San Francisco AIDS Foundation | Non Government Organization | 7.9 | 9.9 | 8.99 | 10.9 | 9.4225 | https://www.sfaf.org/collections/beta/prep-facts-starting-stopping-prep-care/ | https://web.archive.org/web/20220425224657/https://www.sfaf.org/collections/beta/prep-facts-starting-stopping-prep-care/ | PrEP | Both | Oral & Injection PrEP | General | Website |
| 26 | San Francisco AIDS Foundation | Non Government Organization | 8.4 | 10.5 | 10.79 | 10.4 | 10.0225 | https://www.sfaf.org/collections/beta/prep-facts-what-is-prep/ | https://web.archive.org/web/20220425224709/https://www.sfaf.org/collections/beta/prep-facts-what-is-prep/ | PrEP | Both | Oral & Injection PrEP | General | Website |
| 27 | San Francisco AIDS Foundation | Non Government Organization | 7.6 | 10.8 | 9.05 | 9.4 | 9.2125 | https://www.sfaf.org/collections/beta/prep-facts-who-can-take-prep/ | https://web.archive.org/web/20220425224743/https://www.sfaf.org/collections/beta/prep-facts-who-can-take-prep/ | PrEP | Both | Oral & Injection PrEP | General | Website |
| 28 | U.S. Department of Veterans Affairs | The US Government | 6.3 | 8.7 | 8.05 | 7.2 | 7.5625 | https://www.hiv.va.gov/pdf/HIV-PrEP-Factsheet-508.pdf | N/A | PrEP | Both | Oral | General | Brochure |
| 29 | U.S. Department of Veterans Affairs | The US Government | 6.4 | 10.3 | 8 | 7.3 | 8 | https://www.hiv.va.gov/pdf/prep-faqs.pdf | https://web.archive.org/web/20220419232835/https://www.hiv.va.gov/pdf/prep-faqs.pdf | PrEP | Both | Oral | General | Information Sheet |
| 30 | AETC Pacific - AIDS Education & Training Center | The US Government | 11.1 | 13.2 | 13.11 | 13 | 12.6025 | https://www.cdph.ca.gov/Programs/CID/DOA/CDPH%20Document%20Library/QuickClinicalGuide_PrEP_ADA.pdf | https://web.archive.org/web/20220515155830/https://www.cdph.ca.gov/Programs/CID/DOA/CDPH%20Document%20Library/QuickClinicalGuide_PrEP_ADA.pdf | PrEP | Search Engine | Oral | Provider | Information Sheet |
| 31 | aidsmap | Non Government Organization | 5.9 | 8.6 | 8.51 | 7.5 | 7.6275 | https://www.aidsmap.com/about-hiv/what-are-side-effects-truvada-used-prep | https://web.archive.org/web/20220515155852/https://www.aidsmap.com/about-hiv/what-are-side-effects-truvada-used-prep | PrEP | Search Engine | Oral | General | Website |
| 32 | aidsmap | Non Government Organization | 10.3 | 13 | 11.31 | 13.1 | 11.9275 | https://www.aidsmap.com/news/jul-2020/demand-prep-highly-effective-some-may-find-it-confusing | https://web.archive.org/web/20220515155959/https://www.aidsmap.com/news/jul-2020/demand-prep-highly-effective-some-may-find-it-confusing | PrEP | Search Engine | Oral | General | Website |
| 33 | ViiV-Healthcare | For Profit | 11.2 | 12.7 | 12.07 | 16.1 | 13.0175 | https://apretude.com/?&utm_source=bing&utm_medium=cpc&utm_campaign=MB~APRE_CN~UNBRANDED-MEDICATION-PHRASE_CA~UB_MT~PHR_SB~RX_FF~S;PH;UB;INF;DTC;TRE&utm_term=hiv%20pre%20exposure%20prophylaxis&utm_content=Understanding%20PrEP&gclid=65a721a6f4c61417c04043d45fd1cea3&gclsrc=3p.ds | https://web.archive.org/web/20220515160010/https://apretude.com/ | PrEP - Apretude | Search Engine | Injection | General | Website |
| 34 | ViiV-Healthcare | For Profit | 12.8 | 13.1 | 12.2 | 18 | 14.025 | https://apretude.com/about-apretude/what-is-apretude/ | https://web.archive.org/web/20220516021438/https://apretude.com/about-apretude/what-is-apretude/ | PrEP - Apretude (Main page) | Search Engine | Injection | General | Website |
| 35 | ViiV-Healthcare | For Profit | 10.2 | 11.7 | 11.67 | 14.5 | 12.0175 | https://apretude.com/starting-apretude/asking-about-apretude/ | https://web.archive.org/web/20220515160119/https://apretude.com/starting-apretude/asking-about-apretude/ | PrEP - Apretude | Search Engine | Injection | General | Website |
| 36 | ViiV-Healthcare | For Profit | 7.4 | 10.4 | 10.49 | 9.1 | 9.3475 | https://apretude.com/starting-apretude/faqs/ | https://web.archive.org/web/20220515160150/https://apretude.com/starting-apretude/faqs/ | PrEP - Apretude | Search Engine | Injection | General | Website |
| 37 | ViiV-Healthcare | For Profit | 10.8 | 12.5 | 12.36 | 14.3 | 12.49 | https://apretude.com/starting-apretude/steps-to-starting/ | https://web.archive.org/web/20220515160358/https://apretude.com/starting-apretude/steps-to-starting/ | PrEP - Apretude | Search Engine | Injection | General | Website |
| 38 | ViiV-Healthcare | For Profit | 8 | 11.8 | 10.78 | 10.1 | 10.17 | https://apretude.com/apretude-cost/ | https://web.archive.org/web/20220515160331/https://apretude.com/apretude-cost/ | PrEP - Apretude | Search Engine | Injection | General | Website |
| 39 | ViiV-Healthcare | For Profit | 12.7 | 13.5 | 11.61 | 16 | 13.4525 | https://apretude.com/about-prep/what-is-prep/ | https://web.archive.org/web/20220515160402/https://apretude.com/about-prep/what-is-prep/ | PrEP - Apretude | Search Engine | Oral & Injection PrEP | General | Website |
| 40 | ViiV-Healthcare | For Profit | 12.9 | 15.2 | 14.56 | 14.7 | 14.34 | https://apretudehcp.com/?utm_source=bing&utm_medium=cpc&utm_term=hiv%20prep%20drug&utm_campaign=BS%20-%20Unbranded%20HIV-1%20PrEP%20PH&gclid=9d7bb2b21e831d33107ed0df76cdb103&gclsrc=3p.ds | https://web.archive.org/web/20220515160434/https://apretudehcp.com/ | PrEP - Apretude | Search Engine | Injection | General | Website |
| 41 | ViiV-Healthcare | For Profit | 23.7 | 21.7 | 17.36 | 29.9 | 23.165 | https://apretudehcp.com/content/dam/cf-viiv/apretude-hcp/en_US/pdfs/APRETUDE%20Getting%20Started%20Guide%202022%20PDF.pdf | https://web.archive.org/web/20220516021443/https://apretudehcp.com/content/dam/cf-viiv/apretude-hcp/en_US/pdfs/APRETUDE%20Getting%20Started%20Guide%202022%20PDF.pdf | PrEP - Apretude | Search Engine | Injection | Provider | Website |
| 42 | AVAC - Global Advocacy for HIV Prevention | Non Government Organization | 9.2 | 12.1 | 9.92 | 10.5 | 10.43 | https://www.avac.org/sites/default/files/u3/FAQ_PrEP_South_Africa.pdf | https://web.archive.org/web/20220515165330/https://apretudehcp.com/content/dam/cf-viiv/apretude-hcp/en_US/pdfs/APRETUDE%20Getting%20Started%20Guide%202022%20PDF.pdf | PrEP | Search Engine | Oral | General | Information Sheet |
| 43 | BetterHealth Channel | Non-US Government/other Public Health Organization | 8 | 11.2 | 9.85 | 9.2 | 9.5625 | https://www.betterhealth.vic.gov.au/health/conditionsandtreatments/pre-exposure-prophylaxis-for-HIV-prevention | https://web.archive.org/web/20220515165411/https://www.betterhealth.vic.gov.au/health/conditionsandtreatments/pre-exposure-prophylaxis-for-HIV-prevention | PrEP | Search Engine | Oral | General | Website |
| 44 | BetterHealth Channel | Non-US Government/other Public Health Organization | 9.1 | 11.8 | 11.02 | 11.4 | 10.83 | https://www.betterhealth.vic.gov.au/health/conditionsandtreatments/hiv-and-aids | https://web.archive.org/web/20220515165416/https://www.betterhealth.vic.gov.au/health/conditionsandtreatments/hiv-and-aids | PrEP | Search Engine | General PrEP | General | Website |
| 45 | BLACK AIDS INSTITUTE | Non Government Organization | 6.2 | 10.2 | 9.74 | 8.4 | 8.635 | https://blackaids.org/wp-content/uploads/2020/10/18-prep-women-brochure3.pdf | https://web.archive.org/web/20220515165626/https://blackaids.org/wp-content/uploads/2020/10/18-prep-women-brochure3.pdf | PrEP | Search Engine | Oral | General | Brochure |
| 46 | BLACK AIDS INSTITUTE | Non Government Organization | 8.9 | 11.8 | 11.02 | 11.2 | 10.73 | https://blackaids.org/wp-content/uploads/2020/10/18-aids-101-black-women-and-hiv-brochure1.pdf | https://web.archive.org/web/20220516021532/https://blackaids.org/wp-content/uploads/2020/10/18-aids-101-black-women-and-hiv-brochure1.pdf | PrEP | Search Engine | General PrEP | General | Brochure |
| 47 | BLACK AIDS INSTITUTE | Non Government Organization | 8.1 | 11.5 | 8.99 | 9.6 | 9.5475 | http://prep.bwhi.org/prep/what-is-prep/ | http://web.archive.org/web/20220515165659/http://prep.bwhi.org/prep/what-is-prep/ | PrEP | Search Engine | Oral | General | Website |
| 48 | BLACK AIDS INSTITUTE | Non Government Organization | 7.2 | 10.7 | 7.19 | 7.2 | 8.0725 | http://prep.bwhi.org/clientuploads/prep/Prep_Infographic.pdf | https://web.archive.org/web/20220515165650/http://prep.bwhi.org/clientuploads/prep/Prep_Infographic.pdf | PrEP | Search Engine | Oral | General | Brochure |
| 49 | BLACK AIDS INSTITUTE | Non Government Organization | 6.3 | 9.1 | 7.47 | 6.6 | 7.3675 | http://prep.bwhi.org/prep/prep-faq/ | https://web.archive.org/web/20220515165657/http://prep.bwhi.org/prep/prep-faq/ | PrEP | Search Engine | Oral | General | Website |
| 50 | BRIDGING ACCESS to CARE Inc. | Non Government Organization | 9.2 | 11.5 | 9.51 | 10.2 | 10.1025 | https://1j9ygnb58bq2s4b694au9ru2-wpengine.netdna-ssl.com/wp-content/uploads/2018/06/Brochure-PrEP.pdf | https://web.archive.org/web/20220515170331/https://1j9ygnb58bq2s4b694au9ru2-wpengine.netdna-ssl.com/wp-content/uploads/2018/06/Brochure-PrEP.pdf | PrEP | Search Engine | Oral | General | Brochure |
| 51 | California Department of Public Health, Office of AIDS - State of California Health and Human Services Agency | The US Government | 11.1 | 13.7 | 13.63 | 13.4 | 12.9575 | https://kernpublichealth.com/wp-content/uploads/PrEP-AP-Client-FAQ-3.21.18-1.pdf. https://www.cdph.ca.gov/Programs/CID/DOA/CDPH%20Document%20Library/PrEP-AP%20Enrollment%20Worker%20FAQ%20(3.21.18).pdf | https://web.archive.org/web/20220515170443/https://www.cdph.ca.gov/Programs/CID/DOA/CDPH%20Document%20Library/PrEP-AP%20Enrollment%20Worker%20FAQ%20%283.21.18%29.pdf | PrEP | Search Engine | Oral | General | Information Sheet |
| 52 | Centers for Disease Control and Prevention | The US Government | 6.8 | 9.5 | 8.63 | 8.3 | 8.3075 | https://www.cdc.gov/hiv/pdf/library/consumer-info-sheets/cdc-hiv-consumer-info-sheet-safer-sex-101.pdf | https://web.archive.org/web/20220515170351/https://www.cdc.gov/hiv/pdf/library/consumer-info-sheets/cdc-hiv-consumer-info-sheet-safer-sex-101.pdf | HIV | Search Engine | General PrEP | General | Brochure |
| 53 | Centers for Disease Control and Prevention | The US Government | 6.3 | 8.5 | 9.62 | 8.7 | 8.28 | https://www.cdc.gov/hiv/pdf/library/consumer-info-sheets/cdc-hiv-consumer-info-sheet-injecting-drugs-101.pdf | https://web.archive.org/web/20220515170406/https://www.cdc.gov/hiv/pdf/library/consumer-info-sheets/cdc-hiv-consumer-info-sheet-injecting-drugs-101.pdf | HIV | Search Engine | General PrEP | General | Brochure |
| 54 | Centers for Disease Control and Prevention | The US Government | 9.3 | 11.7 | 11.3 | 10.7 | 10.75 | https://www.cdc.gov/hiv/basics/prep.html | https://web.archive.org/web/20220515170526/https://www.cdc.gov/hiv/basics/prep.html | PrEP | Search Engine | General PrEP | General | Website |
| 55 | Centers for Disease Control and Prevention | The US Government | 5.7 | 8.8 | 8.05 | 6.7 | 7.3125 | https://www.cdc.gov/hiv/basics/prep/about-prep.html | https://web.archive.org/web/20220613220341/https://www.cdc.gov/hiv/basics/prep/about-prep.html | PrEP | Search Engine | Oral | General | Website |
| 56 | Centers for Disease Control and Prevention | The US Government | 10.1 | 12 | 8.88 | 10.8 | 10.445 | https://www.cdc.gov/hiv/basics/prep/on-demand-prep.html | https://web.archive.org/web/20220515170538/https://www.cdc.gov/hiv/basics/prep/on-demand-prep.html | PrEP | Search Engine | Oral | General | Website |
| 57 | Centers for Disease Control and Prevention | The US Government | 6.8 | 10 | 9.61 | 7.8 | 8.5525 | https://www.cdc.gov/hiv/basics/prep/paying-for-prep/index.html | https://web.archive.org/web/20220613221831/https://www.cdc.gov/hiv/basics/prep/paying-for-prep/index.html | PrEP | Search Engine | General PrEP | General | Website |
| 58 | Centers for Disease Control and Prevention | The US Government | 12.5 | 12.8 | 8.6 | 14.6 | 12.125 | https://www.cdc.gov/hiv/basics/prep/prep-decision.html | https://web.archive.org/web/20220613221859/https://www.cdc.gov/hiv/basics/prep/prep-decision.html | PrEP | Search Engine | General PrEP | General | Website |
| 59 | Centers for Disease Control and Prevention | The US Government | 6.7 | 10.4 | 8.34 | 7.8 | 8.31 | https://www.cdc.gov/hiv/basics/prep/prep-effectiveness.html | https://web.archive.org/web/20220515172317/https://www.cdc.gov/hiv/basics/prep/prep-effectiveness.html | PrEP | Search Engine | General PrEP | General | Website |
| 60 | Centers for Disease Control and Prevention | The US Government | 6.3 | 8.8 | 7.89 | 8.2 | 7.7975 | https://www.cdc.gov/hiv/basics/prep/starting-stopping-prep.html | https://web.archive.org/web/20220515172315/https://www.cdc.gov/hiv/basics/prep/starting-stopping-prep.html | PrEP | Search Engine | General PrEP | General | Website |
| 61 | Centers for Disease Control and Prevention | The US Government | 6.7 | 9.9 | 8.69 | 8.3 | 8.3975 | https://www.cdc.gov/hiv/clinicians/prevention/prep-and-pep.html | https://web.archive.org/web/20220515172259/https://www.cdc.gov/hiv/clinicians/prevention/prep-and-pep.html | PrEP & PEP | Search Engine | General PrEP | General | Website |
| 62 | Centers for Disease Control and Prevention | The US Government | 7 | 10.4 | 9.97 | 9.4 | 9.1925 | https://www.cdc.gov/stophivtogether/library/topics/prevention/brochures/cdc-lsht-prevention-brochure-prep-medication-guide-patient.pdf | N/A | PrEP | Search Engine | Oral | General | Brochure |
| 63 | Centers for Disease Control and Prevention | The US Government | 10.1 | 12.6 | 12.7 | 12.8 | 12.05 | https://www.cdc.gov/hiv/clinicians/prevention/prep.html | https://web.archive.org/web/20220515172332/https://www.cdc.gov/hiv/clinicians/prevention/prep.html | PrEP | Search Engine | Oral & Injection PrEP | General | Website |
| 64 | Centers for Disease Control and Prevention | The US Government | 7.3 | 10.3 | 10.03 | 8.7 | 9.0825 | https://www.cdc.gov/hiv/risk/prep/index.html | https://web.archive.org/web/20220613221929/https://www.cdc.gov/hiv/risk/prep/index.html | PrEP | Search Engine | General PrEP | General | Website |
| 65 | Centers for Disease Control and Prevention | The US Government | 12.4 | 14.3 | 12.6 | 15 | 13.575 | https://www.cdc.gov/nchhstp/newsroom/docs/factsheets/prep-factsheet-508.pdf | N/A | PrEP | Search Engine | Oral | General | Information Sheet |
| 66 | Champaign-Urbana Public Health District | The US Government | 5.1 | 8.3 | 6.77 | 5.1 | 6.3175 | https://www.c-uphd.org/prep-information.html | N/A | PrEP | Search Engine | General PrEP | General | Website |
| 67 | Cleaveland Clinic | Non Government Organization | 7.5 | 10.6 | 8.99 | 9.2 | 9.0725 | https://health.clevelandclinic.org/what-is-prep-and-who-should-take-it/ | https://web.archive.org/web/20220515184544/https://health.clevelandclinic.org/what-is-prep-and-who-should-take-it/ | PrEP | Search Engine | General PrEP | General | Website |
| 68 | Compass - United States Agency for International Development (USAID) & Johns Hopkins University. | The US Government | 6.1 | 9.9 | 7.53 | 6.7 | 7.5575 | https://www.thecompassforsbc.org/project-examples/frequently-asked-questions-about-pre-exposure-prophylaxis-prep | https://web.archive.org/web/20220515184600/https://www.thecompassforsbc.org/project-examples/frequently-asked-questions-about-pre-exposure-prophylaxis-prep | PrEP | Search Engine | Oral | General | Brochure |
| 69 | dhec - South Carolina Department of Health and Environmental Control | The US Government | 10.4 | 12 | 13.56 | 12.7 | 12.165 | https://scdhec.gov/infectious-diseases/hiv-std-viral-hepatitis/pre-exposure-prophylaxis-prep | https://web.archive.org/web/20220515184608/https://scdhec.gov/infectious-diseases/hiv-std-viral-hepatitis/pre-exposure-prophylaxis-prep | PrEP | Search Engine | Oral & Injection PrEP | General | Website |
| 70 | Centers for Disease Control and Prevention | The US Government | 9.7 | 10.9 | 8.59 | 11.3 | 10.1225 | https://www.cdc.gov/std/hiv/stdfact-std-hiv.htm | https://web.archive.org/web/20220515184705/https://www.cdc.gov/std/hiv/stdfact-std-hiv.htm | STDs & HIV | Search Engine | General PrEP | General | Website |
| 71 | Drugs.com | Non Government Organization | 16 | 17.2 | 14.45 | 18.5 | 16.5375 | https://www.drugs.com/dosage/truvada.html | https://web.archive.org/web/20220515184719/https://www.drugs.com/dosage/truvada.html | Truvada PrEP | Search Engine | Oral | General | Website |
| 72 | Drugs.com | Non Government Organization | 9.4 | 11.4 | 10.91 | 11.9 | 10.9025 | https://www.drugs.com/truvada.html | https://web.archive.org/web/20220515184726/https://www.drugs.com/truvada.html | Truvada PrEP | Search Engine | Oral | General | Website |
| 73 | Elizabeth Glaser Pediatric AIDS Foundation | Non Government Organization | 7.4 | 10.5 | 8.87 | 7.8 | 8.6425 | https://www.pedaids.org/resource/prep-brochure/. https://www.pedaids.org/wp-content/uploads/2019/02/2018PrEPBrochure_11.27.pdf | https://web.archive.org/web/20220515185009/https://www.pedaids.org/wp-content/uploads/2019/02/2018PrEPBrochure_11.27.pdf | PrEP | Search Engine | Oral | General | Brochure |
| 74 | ENDING HIV | Non Government Organization | 9.2 | 11.5 | 9.52 | 11.4 | 10.405 | https://endinghiv.org.au/blog/6-side-effects-of-prep/ | https://web.archive.org/web/20220515184937/https://endinghiv.org.au/blog/6-side-effects-of-prep/ | PrEP | Search Engine | Oral | General | Website |
| 75 | ENDING HIV | Non Government Organization | 9 | 10.6 | 7.49 | 9.7 | 9.1975 | https://endinghiv.org.au/blog/new-ways-to-take-prep/ | https://web.archive.org/web/20220515184936/https://endinghiv.org.au/blog/new-ways-to-take-prep/ | PrEP | Search Engine | Oral | General | Website |
| 76 | Equaltiy Health Center | Non Government Organization | 13.1 | 15.5 | 13.35 | 16.7 | 14.6625 | https://equalityhc.org/prep-for-hiv-prevention/ | https://web.archive.org/web/20220515184938/https://equalityhc.org/prep-for-hiv-prevention/ | PrEP | Search Engine | Oral | General | Website |
| 77 | Family Care of Kent | For Profit | 8.7 | 11.4 | 10.84 | 10.7 | 10.41 | https://www.familycareofkent.com/how-does-prep-work-to-reduce-hiv-infections-2/ | https://web.archive.org/web/20220515184945/https://www.familycareofkent.com/how-does-prep-work-to-reduce-hiv-infections-2/ | PrEP | Search Engine | Oral | General | Website |
| 78 | Family Health Centers of San Diego | Non Government Organization | 7.4 | 10.7 | 8.87 | 8.9 | 8.9675 | https://www.fhcsd.org/prep-information/ | https://web.archive.org/web/20220515185120/https://www.fhcsd.org/prep-information/ | PrEP | Search Engine | Oral | General | Website |
| 79 | Florida HEALTH | The US Government | 11.8 | 14.3 | 14.85 | 15 | 13.9875 | https://www.floridahealth.gov/diseases-and-conditions/aids/PreP/index.html | https://web.archive.org/web/20220515185125/https://www.floridahealth.gov/diseases-and-conditions/aids/PreP/index.html | PrEP & PEP | Search Engine | General PrEP | General | Website |
| 80 | FOLX | For Profit | 10.7 | 12.8 | 10.33 | 12.7 | 11.6325 | https://folxhealth.com/library/what-is-prep | https://web.archive.org/web/20220515185129/https://folxhealth.com/library/what-is-prep/ | PrEP | Search Engine | Oral | General | Website |
| 81 | FOLX | For Profit | 9.6 | 12 | 10.27 | 11.2 | 10.7675 | https://folxhealth.com/library/9-daily-prep-basic-facts/ | https://web.archive.org/web/20220515185139/https://folxhealth.com/library/9-daily-prep-basic-facts/ | PrEP | Search Engine | Oral | General | Website |
| 82 | freddie | For Profit | 10.4 | 13.2 | 10.91 | 11.5 | 11.5025 | https://www.gofreddie.com/medications/truvada | https://web.archive.org/web/20220515185147/https://www.gofreddie.com/medications/truvada | Truvada PrEP | Search Engine | Oral | General | Website |
| 83 | freddie | For Profit | 9.4 | 12.7 | 10.97 | 11.5 | 11.1425 | https://www.gofreddie.com/resources/what-is-prep-hiv | https://web.archive.org/web/20220515185736/https://www.gofreddie.com/resources/what-is-prep-hiv | PrEP | Search Engine | Oral | General | Website |
| 84 | freddie | For Profit | 8 | 10.9 | 8.87 | 8.6 | 9.0925 | https://www.gofreddie.com/resources/what-is-prep-on-demand | https://web.archive.org/web/20220515185744/https://www.gofreddie.com/resources/what-is-prep-on-demand | PrEP | Search Engine | Oral | General | Website |
| 85 | freddie | For Profit | 11.2 | 13.5 | 11.55 | 14 | 12.5625 | https://www.gofreddie.com/resources/who-should-take-prep | https://web.archive.org/web/20220515185754/https://www.gofreddie.com/resources/who-should-take-prep | PrEP | Search Engine | Oral | General | Website |
| 86 | freddie | For Profit | 12.8 | 13.3 | 9.12 | 15.2 | 12.605 | https://www.gofreddie.com/resources/how-to-take-prep | https://web.archive.org/web/20220515185757/https://www.gofreddie.com/resources/how-to-take-prep | PrEP | Search Engine | Oral | General | Website |
| 87 | freddie | For Profit | 9.8 | 12 | 12.06 | 12.2 | 11.515 | https://www.gofreddie.com/resources/prep-side-effects-interactions | https://web.archive.org/web/20220515185806/https://www.gofreddie.com/resources/prep-side-effects-interactions | PrEP | Search Engine | General PrEP | General | Website |
| 88 | freddie | For Profit | 8.7 | 11.5 | 9.45 | 9.8 | 9.8625 | https://www.gofreddie.com/resources/how-effective-is-prep | https://web.archive.org/web/20220515185825/https://www.gofreddie.com/resources/how-effective-is-prep | PrEP | Search Engine | Oral | General | Website |
| 89 | freddie | For Profit | 10.5 | 11.8 | 9.81 | 12 | 11.0275 | https://www.gofreddie.com/resources/how-to-get-prep-canada | https://web.archive.org/web/20220515185950/https://www.gofreddie.com/resources/how-to-get-prep-canada | PrEP | Search Engine | Oral | General | Website |
| 90 | freddie | For Profit | 9.5 | 12.5 | 11.13 | 10.7 | 10.9575 | https://www.gofreddie.com/resources/truvada-generic-prep-descovy | https://web.archive.org/web/20220515185955/https://www.gofreddie.com/resources/truvada-generic-prep-descovy | Truvada PrEP, DESCOVY | Search Engine | Oral | General | Website |
| 91 | freddie | For Profit | 8.7 | 10.8 | 9.68 | 10 | 9.795 | https://www.gofreddie.com/faq | https://web.archive.org/web/20220515190008/https://www.gofreddie.com/faq | PrEP | Search Engine | Oral | General | Website |
| 92 | freddie | For Profit | 7.9 | 10.4 | 7.89 | 8.8 | 8.7475 | https://www.gofreddie.com/magazine/6-myths-about-prep | https://web.archive.org/web/20220515190023/https://www.gofreddie.com/magazine/6-myths-about-prep | PrEP | Search Engine | General PrEP | General | Website |
| 93 | Gay Men's Health | N/A | 8.9 | 11.4 | 10.73 | 11.2 | 10.5575 | http://gaymenshealth.com.au/hiv-prevention/truvada-side-effects/ | `https://web.archive.org/web/20220515190030/http://gaymenshealth.com.au/hiv-prevention/truvada-side-effects/ | Truvada PrEP | Search Engine | Oral | General | Website |
| 94 | Gay Men's Health | N/A | 8.7 | 11.3 | 10.21 | 10.3 | 10.1275 | http://gaymenshealth.com.au/hiv-prevention/hiv-infections/ | https://web.archive.org/web/20220515190156/http://gaymenshealth.com.au/hiv-prevention/hiv-infections/ | HIV | Search Engine | General PrEP | General | Website |
| 95 | Gay Men's Health | N/A | 9 | 11.6 | 10.09 | 10.6 | 10.3225 | http://gaymenshealth.com.au/hiv-prevention/how-to-prevent-hiv/ | https://web.archive.org/web/20220515190211/http://gaymenshealth.com.au/hiv-prevention/how-to-prevent-hiv/ | HIV | Search Engine | General PrEP | General | Website |
| 96 | Gay Men's Health | N/A | 8.2 | 11 | 8.76 | 9.5 | 9.365 | http://gaymenshealth.com.au/hiv-prevention/pre-exposure-prophylaxis/ | https://web.archive.org/web/20220515190223/http://gaymenshealth.com.au/hiv-prevention/pre-exposure-prophylaxis/ | PrEP | Search Engine | Oral | General | Website |
| 97 | Gay Men's Health | N/A | 8.6 | 12.1 | 10.09 | 9.9 | 10.1725 | http://gaymenshealth.com.au/hiv-prevention/truvada/ | https://web.archive.org/web/20220515190415/http://gaymenshealth.com.au/sexual-health/hiv/ | Truvada PrEP | Search Engine | Oral | General | Website |
| 98 | GET PrEP - the Village Pharmacy | For Profit | 8.1 | 11.1 | 9.68 | 9.3 | 9.545 | https://www.get-prep.com/about-prep | https://web.archive.org/web/20220515190418/https://www.get-prep.com/about-prep | PrEP | Search Engine | Oral | General | Website |
| 99 | GET PrEP - the Village Pharmacy | For Profit | 6.6 | 9.4 | 4.58 | 6 | 6.645 | https://www.get-prep.com/is-prep-right-for-me | https://web.archive.org/web/20220515190425/https://www.get-prep.com/is-prep-right-for-me | PrEP | Search Engine | Oral | General | Website |
| 100 | GET PrEP - the Village Pharmacy | For Profit | 7.4 | 10.4 | 7.42 | 7.9 | 8.28 | https://www.get-prep.com/prep-vs-pep | https://web.archive.org/web/20220515190449/https://www.get-prep.com/prep-vs-pep | PrEP & PEP | Search Engine | Oral | General | Website |
| 101 | Gilead Sciences | For Profit | 8.3 | 10.7 | 10.15 | 11 | 10.0375 | https://services.gileadhiv.com/content/pdf/DESCOVY/PrEP/DVYC0312_DVY_Consumer_Brochure_Digital_Update_08_2020_r2v1jl.pdf | https://web.archive.org/web/20220515190459/https://services.gileadhiv.com/content/pdf/DESCOVY/PrEP/DVYC0312_DVY_Consumer_Brochure_Digital_Update_08_2020_r2v1jl.pdf | DESCOVY - PrEP | Search Engine | Oral | General | Brochure |
| 102 | Gilead Sciences | For Profit | 7.5 | 10 | 10.21 | 10.4 | 9.5275 | https://www.descovy.com/side-effects?utm_source=bing&utm_medium=cpc&utm_campaign=USA_MA_SEM_NB_EX_Descovy-DTP-Stay+On+A+Gilead+Medication+PrEP-NB_Copy&utm_content=PrEP_Side+Effects_General&utm_term=prep+side+effects&gclid=32f516deed8c10e9b6f674ab68bada99&gclsrc=3p.ds&msclkid=32f516deed8c10e9b6f674ab68bada99 | https://web.archive.org/web/20220515190508/https://www.descovy.com/side-effects?msclkid=32f516deed8c10e9b6f674ab68bada99 | DESCOVY - PrEP | Search Engine | Oral | General | Website |
| 103 | Gilead Sciences | For Profit | 20.5 | 19.6 | 15.5 | 24.6 | 20.05 | https://www.descovyhcp.com/renal-and-bone-over-time?utm_source=google&utm_medium=cpc&utm_campaign=USA_GO_SEM_C_EX_Descovy-HCP-Learn+About+a+Gilead+Medication+Truvada+Supporting-Standard&utm_content=Truvada_Truvada+PrEP&utm_term=Truvada+prep&gclid=EAIaIQobChMI0vqQ-OjI9wIViXxvBB0SdQysEAAYAiAAEgLWa_D_BwE&gclsrc=aw.ds | N/A | DESCOVY - PrEP | Search Engine | Oral | General | Website |
| 104 | Gilead Sciences | For Profit | 6.1 | 10.1 | 7.36 | 7.5 | 7.765 | https://www.truvada.com/is-truvada-right-for-me/taking-truvada-for-prep | https://web.archive.org/web/20220613220928/https://www.truvada.com/is-truvada-right-for-me/taking-truvada-for-prep | Truvad PrEP | Search Engine | Oral | General | Website |
| 105 | Gilead Sciences | For Profit | 7.1 | 10.7 | 10.32 | 9.9 | 9.505 | https://www.truvada.com/what-is-truvada/side-effects | https://web.archive.org/web/20220613220407/https://www.truvada.com/what-is-truvada/side-effects | Truvad PrEP | Search Engine | Oral | General | Website |
| 106 | GoodRX Health | For Profit | 9.4 | 11.4 | 9.28 | 10.4 | 10.12 | https://www.goodrx.com/truvada/truvada-hiv-prep-cost-generic-how-to-save | https://web.archive.org/web/20220515233700/https://www.goodrx.com/truvada/truvada-hiv-prep-cost-generic-how-to-save | Truvad PrEP | Search Engine | Oral | General | Website |
| 107 | Harvard Health Publishing - Harvard Medical School | For Profit | 9.4 | 12.3 | 10.97 | 11.6 | 11.0675 | https://www.health.harvard.edu/blog/prep-prevents-hiv-so-why-arent-more-people-taking-it-2019100417942 | https://web.archive.org/web/20220515233712/https://www.health.harvard.edu/blog/prep-prevents-hiv-so-why-arent-more-people-taking-it-2019100417942 | PrEP | Search Engine | Oral | General | Website |
| 108 | healthline | Non Government Organization | 9.2 | 11.5 | 9.86 | 10.5 | 10.265 | https://www.healthline.com/health/healthy-sex/talk-about-prep-hiv-aids | https://web.archive.org/web/20220515233722/https://www.healthline.com/health/healthy-sex/talk-about-prep-hiv-aids | PrEP | Search Engine | Oral | General | Website |
| 109 | healthline | Non Government Organization | 9.1 | 12.5 | 10.61 | 11 | 10.8025 | https://www.healthline.com/health/hiv-aids/hiv-prevention/hiv-prep | https://web.archive.org/web/20220515233825/https://www.healthline.com/health/hiv-aids/hiv-prevention/hiv-prep | PrEP | Search Engine | Oral | General | Website |
| 110 | U.S. Department of Health and Human Services - Office of Infectious Disease and HIV/AIDS Policy | The US Government | 8.9 | 11.3 | 9.74 | 10.1 | 10.01 | https://www.hiv.gov/federal-response/ending-the-hiv-epidemic/prep-program | https://web.archive.org/web/20220515233846/https://www.hiv.gov/federal-response/ending-the-hiv-epidemic/prep-program | PrEP | Search Engine | Oral | General | Website |
| 111 | U.S. Department of Health and Human Services - Office of Infectious Disease and HIV/AIDS Policy | The US Government | 7.1 | 10.8 | 9.97 | 9.5 | 9.3425 | https://www.hiv.gov/hiv-basics/hiv-prevention/using-hiv-medication-to-reduce-risk/pre-exposure-prophylaxis | https://web.archive.org/web/20220515233852/https://www.hiv.gov/hiv-basics/hiv-prevention/using-hiv-medication-to-reduce-risk/pre-exposure-prophylaxis | PrEP | Search Engine | Oral & Injection PrEP | General | Website |
| 112 | U.S. Department of Health and Human Services - Office of Infectious Disease and HIV/AIDS Policy | The US Government | 7.8 | 10.7 | 9.56 | 9 | 9.265 | https://www.hiv.gov/hiv-basics/overview/about-hiv-and-aids/what-are-hiv-and-aids | https://web.archive.org/web/20220515233908/https://www.hiv.gov/hiv-basics/overview/about-hiv-and-aids/what-are-hiv-and-aids | HIV-AIDS | Search Engine | General PrEP | General | Website |
| 113 | U.S. Department of Health and Human Services - Office of Infectious Disease and HIV/AIDS Policy | The US Government | 10.8 | 12.4 | 11.9 | 13.7 | 12.2 | https://www.hiv.gov/hiv-basics/hiv-prevention/using-hiv-medication-to-reduce-risk/hiv-treatment-as-prevention | https://web.archive.org/web/20220515234040/https://www.hiv.gov/hiv-basics/hiv-prevention/using-hiv-medication-to-reduce-risk/hiv-treatment-as-prevention | HIV | Search Engine | General PrEP | General | Website |
| 114 | U.S. Department of Health and Human Services - Office of Infectious Disease and HIV/AIDS Policy | The US Government | 8.8 | 11 | 9.8 | 10.1 | 9.925 | https://www.hiv.gov/hiv-basics/hiv-prevention/reducing-sexual-risk/preventing-sexual-transmission-of-hiv | https://web.archive.org/web/20220515234153/https://www.hiv.gov/hiv-basics/hiv-prevention/reducing-sexual-risk/preventing-sexual-transmission-of-hiv | HIV | Search Engine | General PrEP | General | Website |
| 115 | U.S. Department of Health and Human Services - Office of Infectious Disease and HIV/AIDS Policy | The US Government | 8.2 | 11.1 | 8.65 | 9.5 | 9.3625 | https://www.hiv.gov/hiv-basics/hiv-prevention/reducing-risk-from-alcohol-and-drug-use/alcohol-and-hiv-risk | https://web.archive.org/web/20220515234204/https://www.hiv.gov/hiv-basics/hiv-prevention/reducing-risk-from-alcohol-and-drug-use/alcohol-and-hiv-risk | HIV | Search Engine | General PrEP | General | Website |
| 116 | U.S. Department of Health and Human Services - Office of Infectious Disease and HIV/AIDS Policy | The US Government | 8.8 | 10.7 | 9.75 | 11.3 | 10.1375 | https://www.hiv.gov/hiv-basics/hiv-prevention/reducing-risk-from-alcohol-and-drug-use/substance-use-and-hiv-risk | https://web.archive.org/web/20220515234213/https://www.hiv.gov/hiv-basics/hiv-prevention/reducing-risk-from-alcohol-and-drug-use/substance-use-and-hiv-risk | HIV | Search Engine | General PrEP | General | Website |
| 117 | U.S. Department of Health and Human Services - Office of Infectious Disease and HIV/AIDS Policy | The US Government | 7.8 | 10.7 | 9.97 | 10.3 | 9.6925 | https://www.hiv.gov/hiv-basics/hiv-prevention/reducing-mother-to-child-risk/preventing-mother-to-child-transmission-of-hiv | https://web.archive.org/web/20220515234222/https://www.hiv.gov/hiv-basics/hiv-prevention/reducing-mother-to-child-risk/preventing-mother-to-child-transmission-of-hiv | HIV | Search Engine | General PrEP | General | Website |
| 118 | U.S. Department of Health and Human Services - Office of Infectious Disease and HIV/AIDS Policy | The US Government | 10.4 | 13.5 | 12.41 | 12.7 | 12.2525 | https://www.hiv.gov/hiv-basics/hiv-prevention/potential-future-options/long-acting-prep | https://web.archive.org/web/20220515234229/https://www.hiv.gov/hiv-basics/hiv-prevention/potential-future-options/long-acting-prep | PrEP | Search Engine | Injection | General | Website |
| 119 | U.S. Department of Health and Human Services - Office of Infectious Disease and HIV/AIDS Policy | The US Government | 13.6 | 15.6 | 14.45 | 16.5 | 15.0375 | https://www.hiv.gov/hiv-basics/hiv-prevention/potential-future-options/microbicides | https://web.archive.org/web/20220515234236/https://www.hiv.gov/hiv-basics/hiv-prevention/potential-future-options/microbicides | HIV | Search Engine | General PrEP | General | Website |
| 120 | U.S. Department of Health and Human Services - Office of Infectious Disease and HIV/AIDS Policy | The US Government | 8 | 10.9 | 9.28 | 9.9 | 9.52 | https://www.hiv.gov/hiv-basics/hiv-testing/learn-about-hiv-testing/understanding-hiv-test-results | https://web.archive.org/web/20220515234418/https://www.hiv.gov/hiv-basics/hiv-testing/learn-about-hiv-testing/understanding-hiv-test-results | HIV | Search Engine | General PrEP | General | Website |
| 121 | HIVPrEP | Non Government Organization | 9.1 | 12.2 | 9.8 | 10.4 | 10.375 | https://hivprep.org/free-prep-truvada/ | https://web.archive.org/web/20220515234438/https://hivprep.org/free-prep-truvada/ | Truvada PrEP | Search Engine | Oral | General | Website |
| 122 | HIVPrEP | Non Government Organization | 9.6 | 12.9 | 10.73 | 11.6 | 11.2075 | https://hivprep.org/how-truvada-works/ | https://web.archive.org/web/20220515234616/https://hivprep.org/how-truvada-works/ | Truvada PrEP | Search Engine | Oral | General | Website |
| 123 | HIVPrEP | Non Government Organization | 9.8 | 12.1 | 11.55 | 12.5 | 11.4875 | https://hivprep.org/prep-hiv-side-effects/ | https://web.archive.org/web/20220515234743/https://hivprep.org/prep-hiv-side-effects/ | PrEP | Search Engine | Oral | General | Website |
| 124 | HIVPrEP | Non Government Organization | 10.3 | 12.8 | 11.48 | 11.7 | 11.57 | https://hivprep.org/truvada-manufacturer/ | https://web.archive.org/web/20220515234749/https://hivprep.org/truvada-manufacturer/ | Truvada PrEP | Search Engine | Oral | General | Website |
| 125 | i-base | For Profit | 7.4 | 8.8 | 8 | 8.5 | 8.175 | https://i-base.info/guides/prep/how-to-take-prep | N/A | PrEP | Search Engine | Oral | General | Website |
| 126 | TERRENCE HUGGINS TRUST (BHIVA - Brirish HIV Association) | Non Government Organization | 9.7 | 10.6 | 7.61 | 10.3 | 9.5525 | https://www.iwantprepnow.co.uk/how-to-take-prep/ | https://web.archive.org/web/20220515234802/https://www.iwantprepnow.co.uk/how-to-take-prep/ | PrEP | Search Engine | Oral | General | Website |
| 127 | Kaiser Permanente | For Profit | 7.3 | 10.4 | 7.54 | 8.9 | 8.535 | https://mydoctor.kaiserpermanente.org/ncal/Images/PrEP%20FAQs_tcm75-947123.pdf | N/A | PrEP | Search Engine | Oral | General | Information Sheet |
| 128 | MARIN HEALTH AND HUMAN SERVICES | The US Government | 11.2 | 13.9 | 10.85 | 12.5 | 12.1125 | https://www.marinhhs.org/prep-information-health-care-providers. https://www.marinhhs.org/pre-exposure-prophylaxis-prep | https://web.archive.org/web/20220515234818/https://www.marinhhs.org/pre-exposure-prophylaxis-prep | PrEP | Search Engine | Oral | General | Website |
| 129 | marshal | For Profit | 5.6 | 10 | 8.86 | 7.3 | 7.94 | https://heymarshal.com/?gclid=Cj0KCQjwma6TBhDIARIsAOKuANxsOV4H10gRrD0aTprIEaEXHeKoilSuFZNevkO4rOUMdRTS6lIm15UaAv7EEALw_wcB. https://heymarshal.com/faq/ | https://web.archive.org/web/20220515234948/https://heymarshal.com/ | PrEP | Search Engine | General PrEP | General | Website |
| 130 | Massachusetts Department of Public Health | The US Government | 14 | 16.3 | 15.72 | 16.9 | 15.73 | https://view.officeapps.live.com/op/view.aspx?src=https%3A%2F%2Fwww.mass.gov%2Fdoc%2Ffrequently-asked-questions-pre-exposure-prophylaxis-prep-for-hiv-infection%2Fdownload&wdOrigin=BROWSELINK | https://web.archive.org/web/20220515235004/https://view.officeapps.live.com/op/view.aspx?src=https%3A%2F%2Fwww.mass.gov%2Fdoc%2Ffrequently-asked-questions-pre-exposure-prophylaxis-prep-for-hiv-infection%2Fdownload&wdOrigin=BROWSELINK | PrEP | Search Engine | General PrEP | General | Information Sheet |
| 131 | Massachusetts Department of Public Health | The US Government | 8.8 | 10.8 | 7.54 | 9.4 | 9.135 | https://www.mass.gov/files/documents/2016/08/wq/prep-for-patients.pdf | https://web.archive.org/web/20220515235013/https://www.mass.gov/files/documents/2016/08/wq/prep-for-patients.pdf | PrEP | Search Engine | Oral | General | Information Sheet |
| 132 | Mayo Clinic | Non Government Organization | 8.3 | 11.2 | 11.02 | 10.6 | 10.28 | https://www.mayoclinic.org/diseases-conditions/hiv-aids/expert-answers/prep-hiv/faq-20456940 | https://web.archive.org/web/20220515235022/https://www.mayoclinic.org/diseases-conditions/hiv-aids/expert-answers/prep-hiv/faq-20456940 | PrEP | Search Engine | General PrEP | General | Website |
| 133 | Mayo Clinic | Non Government Organization | 9.1 | 11.6 | 11.36 | 10.6 | 10.665 | https://www.mayoclinic.org/diseases-conditions/hiv-aids/symptoms-causes/syc-20373524 | https://web.archive.org/web/20220515235241/https://www.mayoclinic.org/diseases-conditions/hiv-aids/symptoms-causes/syc-20373524 | HIV-AIDS | Search Engine | General PrEP | General | Website |
| 134 | MedicalNewsToday | For Profit | 8.6 | 11.8 | 10.84 | 10.7 | 10.485 | https://www.medicalnewstoday.com/articles/drugs-truvada-for-prep#introduction | https://web.archive.org/web/20220515235249/https://www.medicalnewstoday.com/articles/drugs-truvada-for-prep | Truvada PrEP | Search Engine | Oral | General | Website |
| 135 | Michigan Department of Health & Human Services | The US Government | 7.4 | 10.4 | 10.2 | 8.8 | 9.2 | https://www.michigan.gov/-/media/Project/Websites/mdhhs/Folder3/Folder45/Folder2/Folder145/Folder1/Folder245/MDHHS_PrEP_Brochure.pdf?rev=d21fc8116e234f84bc95b6817507bb77 | https://web.archive.org/web/20220515235258/https://www.michigan.gov/-/media/Project/Websites/mdhhs/Folder3/Folder45/Folder2/Folder145/Folder1/Folder245/MDHHS_PrEP_Brochure.pdf?rev=d21fc8116e234f84bc95b6817507bb77 | PrEP | Search Engine | General PrEP | General | Brochure |
| 136 | mira - TalktoMira, Inc | For Profit | 9.1 | 12.7 | 10.55 | 11 | 10.8375 | https://www.talktomira.com/post/how-much-is-prep-how-to-get-it-for-free-truvana-descovy | https://web.archive.org/web/20220515235308/https://www.talktomira.com/post/how-much-is-prep-how-to-get-it-for-free-truvana-descovy | PrEP | Search Engine | General PrEP | General | Website |
| 137 | mistr | For Profit | 7.9 | 10.8 | 9.39 | 8.9 | 9.2475 | https://heymistr.com/?gclid=Cj0KCQjwma6TBhDIARIsAOKuANwE_xf-vAqDzDqmRm0-LoUizvRYmoArPUI8ga4RIrSw2YGYsl-Xp5QaAnyAEALw_wcB. https://heymistr.com/faq/ | https://web.archive.org/web/20220515235329/https://heymistr.com/faq/ | PrEP | Search Engine | General PrEP | General | Website |
| 138 | National Clinician Consultation Center - University of California, San Francisco | For Profit | 15.4 | 16.3 | 14.22 | 19 | 16.23 | https://nccc.ucsf.edu/clinical-resources/prep-guidelines-and-resources/. https://www.cdc.gov/hiv/pdf/risk/prep/cdc-hiv-prep-guidelines-2021.pdf | N/A | PrEP | Search Engine | Oral & Injection PrEP | Provider | Information Sheet |
| 139 | New York State Department of Health | The US Government | 9.5 | 12.7 | 11.14 | 11.9 | 11.31 | https://health.ny.gov/diseases/aids/general/prep/faqs.htm | https://web.archive.org/web/20220515235459/https://health.ny.gov/diseases/aids/general/prep/faqs.htm | PrEP & PEP | Search Engine | Oral | General | Website |
| 140 | New York State Department of Health | The US Government | 13.9 | 16.1 | 14.39 | 17 | 15.3475 | https://www.health.ny.gov/diseases/aids/general/prep/truvada.htm | https://web.archive.org/web/20220515235505/https://www.health.ny.gov/diseases/aids/general/prep/truvada.htm | Truvada PrEP, PEP | Search Engine | Oral | General | Website |
| 141 | New York State Department of Health | The US Government | 5.6 | 9.2 | 9.03 | 7.7 | 7.8825 | https://www.health.ny.gov/publications/0130.pdf | N/A | PrEP | Search Engine | General PrEP | General | Information Sheet |
| 142 | New York State Department of Health | The US Government | 9.7 | 12.3 | 11.37 | 11.1 | 11.1175 | https://www.health.ny.gov/publications/0265/#:~:text=Truvada%2C%20the%20PrEP%20drug%2C%20costs%20between%20%248%2C000%20and,medical%20insurer%20to%20see%20if%20PrEP%20is%20covered. | https://web.archive.org/web/20220515235702/https://www.health.ny.gov/publications/0265/ | PrEP | Search Engine | General PrEP | General | Website |
| 143 | New York State Department of Health | The US Government | 10.9 | 11.9 | 9.93 | 12.4 | 11.2825 | https://www.health.ny.gov/diseases/aids/general/publications/. https://www.health.ny.gov/diseases/aids/providers/testing/guidance/negativetestresults.htm | https://web.archive.org/web/20220515235722/https://www.health.ny.gov/diseases/aids/providers/testing/guidance/negativetestresults.htm | HIV | Search Engine | General PrEP | General | Website |
| 144 | North Dakota Health | The US Government | 7.7 | 10.8 | 9.92 | 10.3 | 9.68 | https://www.health.nd.gov/HIV/Prevent/PrEP | https://web.archive.org/web/20220516000057/https://www.health.nd.gov/HIV/Prevent/PrEP | PrEP | Search Engine | General PrEP | General | Website |
| 145 | NOVUS | For Profit | 9.6 | 12.8 | 11.37 | 12.2 | 11.4925 | https://novusacs.com/how-does-prep-work-to-prevent-hiv/ | https://web.archive.org/web/20220516001310/https://novusacs.com/how-does-prep-work-to-prevent-hiv/ | PrEP | Search Engine | General PrEP | General | Website |
| 146 | nurx | For Profit | 8.5 | 11.2 | 9.75 | 10.8 | 10.0625 | https://www.nurx.com/prep/?utm_campaign=PrEP_Exact_NBS_NURX&utm_content=prep&g_adtype=search&utm_term=prep&utm_medium=cpc&utm_source=google&g_acctid=687-940-1110&g_campaign=PrEP_Exact_NBS_NURX&g_campaignid=1657267658&g_adgroupid=67418863807&g_adid=498850611321&g_keyword=prep&g_keywordid=kwd-296931818053&g_network=g&gclid=Cj0KCQjwma6TBhDIARIsAOKuANzob-precyh8rTDji3Ha_ItMfTZ74eKl6UvPglK2fdv1Z-X3XZUc1gaAkaeEALw_wcB. https://www.nurx.com/faq/category/hiv-prevention-faqs/. https://www.nurx.com/faq/can-hiv-be-transmitted-to-newborns/ | https://web.archive.org/web/20220516001436/https://www.nurx.com/faq/category/hiv-prevention-faqs/.%20https:/www.nurx.com/faq/can-hiv-be-transmitted-to-newborns/ | PrEP | Search Engine | General PrEP | General | Website |
| 147 | nurx | For Profit | 9.9 | 12.8 | 10.39 | 11.5 | 11.1475 | https://www.nurx.com/faq/what-is-the-risk-of-getting-hiv-from-oral-sex/ | https://web.archive.org/web/20220516001347/https://www.nurx.com/faq/what-is-the-risk-of-getting-hiv-from-oral-sex/ | HIV | Search Engine | General PrEP | General | Website |
| 148 | nurx | For Profit | 8.5 | 10.1 | 9.39 | 9.5 | 9.3725 | https://www.nurx.com/faq/can-i-get-hiv-from-kissing/ | https://web.archive.org/web/20220516001356/https://www.nurx.com/faq/can-i-get-hiv-from-kissing/ | HIV | Search Engine | General PrEP | General | Website |
| 149 | nurx | For Profit | 11.1 | 13.2 | 12.82 | 14 | 12.78 | https://www.nurx.com/faq/how-is-hiv-transmitted/ | https://web.archive.org/web/20220516001534/https://www.nurx.com/faq/how-is-hiv-transmitted/ | HIV | Search Engine | General PrEP | General | Website |
| 150 | nurx | For Profit | 9.5 | 11.9 | 11.31 | 12.2 | 11.2275 | https://www.nurx.com/faq/what-payment-assistance-programs-are-available-for-prep/ | https://web.archive.org/web/20220516001540/https://www.nurx.com/faq/what-payment-assistance-programs-are-available-for-prep/ | PrEP | Search Engine | General PrEP | General | Website |
| 151 | nurx | For Profit | 10.3 | 0 | 13.04 | 12.1 | 8.86 | https://www.nurx.com/faq/does-the-gilead-copay-card-advancing-access-or-paf-grant-cover-my-nurx-prep-home-testing-kit/ | https://web.archive.org/web/20220516001548/https://www.nurx.com/faq/does-the-gilead-copay-card-advancing-access-or-paf-grant-cover-my-nurx-prep-home-testing-kit/ | PrEP | Search Engine | General PrEP | General | Website |
| 152 | nurx | For Profit | 13.7 | 14.1 | 9.7 | 17 | 13.625 | https://www.nurx.com/faq/what-can-i-do-if-i-am-not-in-a-state-that-nurx-serves-and-my-provider-refuses-to-give-me-prep/ | https://web.archive.org/web/20220516001554/https://www.nurx.com/faq/what-can-i-do-if-i-am-not-in-a-state-that-nurx-serves-and-my-provider-refuses-to-give-me-prep/ | PrEP | Search Engine | General PrEP | General | Website |
| 153 | nurx | For Profit | 12.7 | 14 | 12.02 | 14.8 | 13.38 | https://www.nurx.com/faq/how-does-the-gilead-patient-assistance-program-work/ | https://web.archive.org/web/20220516001602/https://www.nurx.com/faq/how-does-the-gilead-patient-assistance-program-work/ | PrEP | Search Engine | General PrEP | General | Website |
| 154 | nurx | For Profit | 11 | 13.1 | 12.07 | 12.7 | 12.2175 | https://www.nurx.com/faq/can-i-use-my-copay-card-for-descovy/ | https://web.archive.org/web/20220516001609/https://www.nurx.com/faq/can-i-use-my-copay-card-for-descovy/ | DESCOVY-PrEP | Search Engine | Oral | General | Website |
| 155 | nurx | For Profit | 9.2 | 12.5 | 12.11 | 11.3 | 11.2775 | https://www.nurx.com/faq/what-questions-should-i-ask-my-insurance-company-if-im-interested-in-prep/ | https://web.archive.org/web/20220516001803/https://www.nurx.com/faq/what-questions-should-i-ask-my-insurance-company-if-im-interested-in-prep/ | PrEP | Search Engine | General PrEP | General | Website |
| 156 | nurx | For Profit | 10.9 | 13.2 | 10.68 | 12.9 | 11.92 | https://www.nurx.com/faq/what-happens-if-results-come-back-hiv-positive-after-using-the-nurx-home-prep-test-kit/ | https://web.archive.org/web/20220516004523/https://www.nurx.com/faq/what-happens-if-results-come-back-hiv-positive-after-using-the-nurx-home-prep-test-kit/ | PrEP | Search Engine | General PrEP | General | Website |
| 157 | nurx | For Profit | 8.7 | 11.8 | 8.99 | 9 | 9.6225 | https://www.nurx.com/faq/how-do-i-take-truvada/ | https://web.archive.org/web/20220516004706/https://www.nurx.com/faq/how-do-i-take-truvada/ | Truvada PrEP | Search Engine | Oral | General | Website |
| 158 | nurx | For Profit | 9.9 | 12.5 | 14.09 | 12.1 | 12.1475 | https://www.nurx.com/faq/what-is-truvada/ | https://web.archive.org/web/20220516004712/https://www.nurx.com/faq/what-is-truvada/ | Truvada PrEP | Search Engine | Oral | General | Website |
| 159 | nurx | For Profit | 8.8 | 11.2 | 14.25 | 11.8 | 11.5125 | https://www.nurx.com/faq/does-prep-protect-against-stis/ | https://web.archive.org/web/20220516004721/https://www.nurx.com/faq/does-prep-protect-against-stis/ | PrEP | Search Engine | General PrEP | General | Website |
| 160 | nurx | For Profit | 10.2 | 11.2 | 9.75 | 11.3 | 10.6125 | https://www.nurx.com/faq/is-prep-safe-to-use-during-pregnancy/ | https://web.archive.org/web/20220516005030/https://www.nurx.com/faq/is-prep-safe-to-use-during-pregnancy/ | PrEP | Search Engine | General PrEP | General | Website |
| 161 | nurx | For Profit | 8.5 | 11.2 | 11.19 | 10.8 | 10.4225 | https://www.nurx.com/faq/what-should-i-do-if-i-take-too-much-descovy/ | https://web.archive.org/web/20220516005037/https://www.nurx.com/faq/what-should-i-do-if-i-take-too-much-descovy/ | DESCOVY-PrEP | Search Engine | Oral | General | Website |
| 162 | nurx | For Profit | 9.1 | 11.6 | 12.24 | 12.4 | 11.335 | https://www.nurx.com/faq/what-is-descovy-used-for/ | https://web.archive.org/web/20220516005045/https://www.nurx.com/faq/what-is-descovy-used-for/ | DESCOVY-PrEP | Search Engine | Oral | General | Website |
| 163 | nurx | For Profit | 9.7 | 12.8 | 10.44 | 11.4 | 11.085 | https://www.nurx.com/faq/can-i-still-get-hiv-if-im-on-prep/ | https://web.archive.org/web/20220516005149/https://www.nurx.com/faq/can-i-still-get-hiv-if-im-on-prep/ | PrEP | Search Engine | General PrEP | General | Website |
| 164 | nurx | For Profit | 9.9 | 11.8 | 8.13 | 10.9 | 10.1825 | https://www.nurx.com/faq/can-i-get-hiv-from-someone-taking-prep/ | https://web.archive.org/web/20220516005158/https://www.nurx.com/faq/can-i-get-hiv-from-someone-taking-prep/ | PrEP | Search Engine | General PrEP | General | Website |
| 165 | nurx | For Profit | 4.6 | 8.3 | 4.86 | 4.2 | 5.49 | https://www.nurx.com/faq/what-if-i-miss-a-dose-of-prep/ | https://web.archive.org/web/20220516005205/https://www.nurx.com/faq/what-if-i-miss-a-dose-of-prep/ | PrEP | Search Engine | Oral | General | Website |
| 166 | nurx | For Profit | 6.8 | 9.6 | 10.37 | 9.6 | 9.0925 | https://www.nurx.com/faq/how-do-i-take-descovy/ | https://web.archive.org/web/20220516005213/https://www.nurx.com/faq/how-do-i-take-descovy/ | DESCOVY-PrEP | Search Engine | Oral | General | Website |
| 167 | nurx | For Profit | 9.4 | 12.8 | 9.17 | 10.1 | 10.3675 | https://www.nurx.com/faq/can-i-take-a-hiv-test-while-on-prep/ | https://web.archive.org/web/20220516005751/https://www.nurx.com/faq/can-i-take-a-hiv-test-while-on-prep/ | PrEP | Search Engine | Oral | General | Website |
| 168 | nurx | For Profit | 5.1 | 11.9 | 7.98 | 6 | 7.745 | https://www.nurx.com/faq/do-alcohol-or-drugs-interact-with-prep/ | https://web.archive.org/web/20220516005758/https://www.nurx.com/faq/do-alcohol-or-drugs-interact-with-prep/ | PrEP | Search Engine | General PrEP | General | Website |
| 169 | nurx | For Profit | 9.2 | 11.7 | 8.88 | 11 | 10.195 | https://www.nurx.com/faq/can-you-take-prep-if-you-are-hiv-positive/ | https://web.archive.org/web/20220516005804/https://www.nurx.com/faq/can-you-take-prep-if-you-are-hiv-positive/ | PrEP | Search Engine | Oral | General | Website |
| 170 | nurx | For Profit | 9.1 | 12 | 11.25 | 11.4 | 10.9375 | https://www.nurx.com/faq/what-is-descovy/ | https://web.archive.org/web/20220516005810/https://www.nurx.com/faq/what-is-descovy/ | DESCOVY-PrEP | Search Engine | Oral | General | Website |
| 171 | nurx | For Profit | 9 | 12.2 | 11.42 | 11.6 | 11.055 | https://www.nurx.com/faq/what-are-hiv-mouth-sores/ | https://web.archive.org/web/20220516005938/https://www.nurx.com/faq/what-are-hiv-mouth-sores/ | HIV | Search Engine | General PrEP | General | Website |
| 172 | nurx | For Profit | 7.2 | 10.8 | 9.45 | 9.2 | 9.1625 | https://www.nurx.com/faq/what-are-the-early-signs-of-an-hiv-infection/ | https://web.archive.org/web/20220516005944/https://www.nurx.com/faq/what-are-the-early-signs-of-an-hiv-infection/ | HIV | Search Engine | General PrEP | General | Website |
| 173 | nurx | For Profit | 7.3 | 10 | 8.29 | 8.3 | 8.4725 | https://www.nurx.com/faq/can-i-get-the-nurx-prep-home-testing-kit-if-i-am-in-rhode-island/ | https://web.archive.org/web/20220516010159/https://www.nurx.com/faq/can-i-get-the-nurx-prep-home-testing-kit-if-i-am-in-rhode-island/ | HIV | Search Engine | General PrEP | General | Website |
| 174 | nurx | For Profit | 7.3 | 10 | 7.65 | 7.8 | 8.1875 | https://www.nurx.com/faq/can-i-get-the-nurx-prep-home-testing-kit-if-i-am-in-new-york/ | https://web.archive.org/web/20220516010206/https://www.nurx.com/faq/can-i-get-the-nurx-prep-home-testing-kit-if-i-am-in-new-york/ | HIV | Search Engine | General PrEP | General | Website |
| 175 | nurx | For Profit | 6 | 8.1 | 7.19 | 7.4 | 7.1725 | https://www.nurx.com/faq/how-do-i-use-the-nurx-prep-home-testing-kit/ | https://web.archive.org/web/20220516010215/https://www.nurx.com/faq/how-do-i-use-the-nurx-prep-home-testing-kit/ | PrEP | Search Engine | General PrEP | General | Website |
| 176 | nurx | For Profit | 4.5 | 7.6 | 7.58 | 6.1 | 6.445 | https://www.nurx.com/faq/how-does-prep-work/ | https://web.archive.org/web/20220516010350/https://www.nurx.com/faq/how-does-prep-work/ | PrEP | Search Engine | Oral | General | Website |
| 177 | nurx | For Profit | 11.1 | 13.7 | 10.74 | 13.2 | 12.185 | https://www.nurx.com/faq/how-do-i-get-a-prescription-for-truvada/ | https://web.archive.org/web/20220516010406/https://www.nurx.com/faq/how-do-i-get-a-prescription-for-truvada/ | Truvada PrEP | Search Engine | Oral | General | Website |
| 178 | nurx | For Profit | 8.9 | 12 | 11.53 | 10.2 | 10.6575 | https://www.nurx.com/faq/im-transgender-is-there-anything-i-need-to-know-before-requesting-a-prescription-for-prep-from-nurx/ | https://web.archive.org/web/20220516010803/https://www.nurx.com/faq/im-transgender-is-there-anything-i-need-to-know-before-requesting-a-prescription-for-prep-from-nurx/ | PrEP | Search Engine | Oral | General | Website |
| 179 | nurx | For Profit | 6.5 | 10.5 | 8.29 | 7.5 | 8.1975 | https://www.nurx.com/faq/how-effective-is-prep/ | https://web.archive.org/web/20220516011111/https://www.nurx.com/faq/how-effective-is-prep/ | PrEP | Search Engine | General PrEP | General | Website |
| 180 | nurx | For Profit | 5.7 | 9.5 | 6.6 | 5.5 | 6.825 | https://www.nurx.com/faq/how-old-do-i-need-to-be-to-start-taking-prep/ | https://web.archive.org/web/20220516011117/https://www.nurx.com/faq/how-old-do-i-need-to-be-to-start-taking-prep/ | PrEP | Search Engine | General PrEP | General | Website |
| 181 | nurx | For Profit | 6.6 | 8.8 | 9.94 | 7.9 | 8.31 | https://www.nurx.com/faq/who-makes-descovy/ | https://web.archive.org/web/20220516011123/https://www.nurx.com/faq/who-makes-descovy/ | DESCOVY-PrEP | Search Engine | Oral | General | Website |
| 182 | nurx | For Profit | 8.9 | 11.2 | 10.61 | 10.6 | 10.3275 | https://www.nurx.com/faq/how-will-my-prep-order-arrive/ | https://web.archive.org/web/20220516011132/https://www.nurx.com/faq/how-will-my-prep-order-arrive/ | PrEP | Search Engine | General PrEP | General | Website |
| 183 | nurx | For Profit | 10.2 | 13 | 12.7 | 12.9 | 12.2 | https://www.nurx.com/faq/how-do-you-get-prep-for-free/ | https://web.archive.org/web/20220516011142/https://www.nurx.com/faq/how-do-you-get-prep-for-free/ | PrEP | Search Engine | General PrEP | General | Website |
| 184 | nurx | For Profit | 8 | 11.2 | 8.47 | 9.3 | 9.2425 | https://www.nurx.com/faq/does-insurance-cover-prep/ | https://web.archive.org/web/20220516011240/https://www.nurx.com/faq/does-insurance-cover-prep/ | PrEP | Search Engine | General PrEP | General | Website |
| 185 | nurx | For Profit | 10.3 | 13.1 | 12.87 | 13 | 12.3175 | https://www.nurx.com/faq/is-descovy-approved-for-prep/ | https://web.archive.org/web/20220516011401/https://www.nurx.com/faq/is-descovy-approved-for-prep/ | PrEP | Search Engine | Oral | General | Website |
| 186 | nurx | For Profit | 8.7 | 11.3 | 9.69 | 11 | 10.1725 | https://www.nurx.com/faq/where-can-i-get-prep-medication/ | https://web.archive.org/web/20220516011422/https://www.nurx.com/faq/where-can-i-get-prep-medication/ | PrEP | Search Engine | General PrEP | General | Website |
| 187 | nurx | For Profit | 9.3 | 12.4 | 11.66 | 12 | 11.34 | https://www.nurx.com/faq/how-do-i-get-prep/ | https://web.archive.org/web/20220516011414/https://www.nurx.com/faq/how-do-i-get-prep/ | PrEP | Search Engine | General PrEP | General | Website |
| 188 | nurx | For Profit | 13.4 | 14.2 | 16.01 | 15.2 | 14.7025 | https://www.nurx.com/faq/who-makes-truvada/ | https://web.archive.org/web/20220516011421/https://www.nurx.com/faq/who-makes-truvada/ | Truvada PrEP | Search Engine | Oral | General | Website |
| 189 | nurx | For Profit | 9.5 | 13.3 | 11.25 | 10.6 | 11.1625 | https://www.nurx.com/faq/what-is-the-difference-between-prep-and-pep/ | https://web.archive.org/web/20220516011427/https://www.nurx.com/faq/what-is-the-difference-between-prep-and-pep/ | PrEP & PEP | Search Engine | General PrEP | General | Website |
| 190 | nurx | For Profit | 8.3 | 12 | 9.86 | 9.5 | 9.915 | https://www.nurx.com/faq/what-is-truvada-used-for/ | https://web.archive.org/web/20220516011434/https://www.nurx.com/faq/what-is-truvada-used-for/ | Truvada PrEP | Search Engine | Oral | General | Website |
| 191 | nurx | For Profit | 9.1 | 12.7 | 9.8 | 10.4 | 10.5 | https://www.nurx.com/faq/what-does-hiv-negative-on-prep-mean/ | https://web.archive.org/web/20220516011541/https://www.nurx.com/faq/what-does-hiv-negative-on-prep-mean/ | PrEP | Search Engine | Oral | General | Website |
| 192 | nurx | For Profit | 9.1 | 12.1 | 10.26 | 10.8 | 10.565 | https://www.nurx.com/faq/how-do-i-get-descovy-for-free/ | https://web.archive.org/web/20220516011549/https://www.nurx.com/faq/how-do-i-get-descovy-for-free/ | PrEP | Search Engine | Oral | General | Website |
| 193 | nurx | For Profit | 8.8 | 11.2 | 8.24 | 10.3 | 9.635 | https://www.nurx.com/faq/what-lab-work-do-i-need-to-do-in-order-to-get-prep/ | https://web.archive.org/web/20220516011556/https://www.nurx.com/faq/what-lab-work-do-i-need-to-do-in-order-to-get-prep/ | PrEP | Search Engine | General PrEP | General | Website |
| 194 | nurx | For Profit | 9.7 | 11.7 | 11.48 | 10.9 | 10.945 | https://www.nurx.com/faq/how-much-does-descovy-cost/ | https://web.archive.org/web/20220516011602/https://www.nurx.com/faq/how-much-does-descovy-cost/ | DESCOVY-PrEP | Search Engine | Oral | General | Website |
| 195 | nurx | For Profit | 10.5 | 12.7 | 10.44 | 12.3 | 11.485 | https://www.nurx.com/faq/where-can-i-buy-truvada/ | https://web.archive.org/web/20220516011608/https://www.nurx.com/faq/where-can-i-buy-truvada/ | Truvada PrEP | Search Engine | Oral | General | Website |
| 196 | nurx | For Profit | 8.4 | 11 | 10.84 | 10.4 | 10.16 | https://www.nurx.com/faq/does-insurance-cover-descovy/ | https://web.archive.org/web/20220516011614/https://www.nurx.com/faq/does-insurance-cover-descovy/ | DESCOVY-PrEP | Search Engine | Oral | General | Website |
| 197 | nurx | For Profit | 11.8 | 13.2 | 12.47 | 14 | 12.8675 | https://www.nurx.com/faq/whats-the-difference-between-descovy-and-truvada/ | https://web.archive.org/web/20220516011721/https://www.nurx.com/faq/whats-the-difference-between-descovy-and-truvada/ | Truvada PrEP, DESCOVY | Search Engine | Oral | General | Website |
| 198 | nurx | For Profit | 10.1 | 12 | 9.12 | 11.9 | 10.78 | https://www.nurx.com/faq/what-is-generic-truvada/ | https://web.archive.org/web/20220516011729/https://www.nurx.com/faq/what-is-generic-truvada/ | Truvada PrEP | Search Engine | Oral | General | Website |
| 199 | nurx | For Profit | 11.4 | 13.1 | 11.72 | 13.1 | 12.33 | https://www.nurx.com/faq/where-can-i-buy-descovy/ | https://web.archive.org/web/20220516011736/https://www.nurx.com/faq/where-can-i-buy-descovy/ | DESCOVY-PrEP | Search Engine | Oral | General | Website |
| 200 | nurx | For Profit | 7.6 | 11.9 | 10.49 | 9.6 | 9.8975 | https://www.nurx.com/faq/how-much-does-truvada-cost/ | https://web.archive.org/web/20220516011738/https://www.nurx.com/faq/how-much-does-truvada-cost/ | Truvada PrEP | Search Engine | Oral | General | Website |
| 201 | nurx | For Profit | 11.8 | 14.4 | 12.82 | 13 | 13.005 | https://www.nurx.com/faq/how-much-does-truvada-cost-with-insurance/ | https://web.archive.org/web/20220516011743/https://www.nurx.com/faq/how-much-does-truvada-cost-with-insurance/ | Truvada PrEP | Search Engine | Oral | General | Website |
| 202 | nurx | For Profit | 10.3 | 12 | 12.98 | 11.8 | 11.77 | https://www.nurx.com/faq/what-is-prep/ | https://web.archive.org/web/20220516011904/https://www.nurx.com/faq/what-is-prep/ | PrEP | Search Engine | General PrEP | General | Website |
| 203 | nurx | For Profit | 7.2 | 10.9 | 7.2 | 8.4 | 8.425 | https://www.nurx.com/faq/how-long-do-i-need-to-take-prep/ | https://web.archive.org/web/20220516011915/https://www.nurx.com/faq/how-long-do-i-need-to-take-prep/ | PrEP | Search Engine | General PrEP | General | Website |
| 204 | nurx | For Profit | 7.6 | 11.2 | 9.51 | 9.7 | 9.5025 | https://www.nurx.com/faq/how-do-i-get-a-prescription-for-descovy/ | https://web.archive.org/web/20220516011926/https://www.nurx.com/faq/how-do-i-get-a-prescription-for-descovy/ | PrEP | Search Engine | General PrEP | General | Website |
| 205 | nurx | For Profit | 9.7 | 12.2 | 12.28 | 10.9 | 11.27 | https://www.nurx.com/faq/how-quickly-does-prep-start-working/ | https://web.archive.org/web/20220516011926/https://www.nurx.com/faq/how-do-i-get-a-prescription-for-descovy/ | PrEP | Search Engine | General PrEP | General | Website |
| 206 | nurx | For Profit | 9.6 | 12.2 | 13.27 | 11.7 | 11.6925 | https://www.nurx.com/faq/how-do-i-get-truvada-for-free/ | https://web.archive.org/web/20220516011942/https://www.nurx.com/faq/how-do-i-get-truvada-for-free/ | PrEP | Search Engine | Oral | General | Website |
| 207 | nurx | For Profit | 10.7 | 13.2 | 10.85 | 12.9 | 11.9125 | https://www.nurx.com/faq/does-insurance-cover-truvada/ | https://web.archive.org/web/20220516012044/https://www.nurx.com/faq/does-insurance-cover-truvada/ | Truvada PrEP | Search Engine | Oral | General | Website |
| 208 | nurx | For Profit | 13.8 | 14.6 | 9.88 | 15.7 | 13.495 | https://www.nurx.com/faq/is-prep-right-for-me/ | https://web.archive.org/web/20220516012003/https://www.nurx.com/faq/is-prep-right-for-me/ | PrEP | Search Engine | General PrEP | General | Website |
| 209 | nurx | For Profit | 11.6 | 13.5 | 10.27 | 12.1 | 11.8675 | https://www.nurx.com/faq/how-long-do-the-side-effects-of-truvada-last/ | https://web.archive.org/web/20220516012137/https://www.nurx.com/faq/how-long-do-the-side-effects-of-truvada-last/ | PrEP | Search Engine | Oral | General | Website |
| 210 | nurx | For Profit | 10.2 | 12.4 | 12.75 | 11.5 | 11.7125 | https://www.nurx.com/faq/what-are-the-side-effects-of-descovy/ | https://web.archive.org/web/20220516012143/https://www.nurx.com/faq/what-are-the-side-effects-of-descovy/ | DESCOVY-PrEP | Search Engine | Oral | General | Website |
| 211 | nurx | For Profit | 7.3 | 10.5 | 9.79 | 8.5 | 9.0225 | https://www.nurx.com/faq/what-are-the-side-effects-of-prep/ | https://web.archive.org/web/20220516012150/https://www.nurx.com/faq/what-are-the-side-effects-of-prep/ | PrEP | Search Engine | Oral | General | Website |
| 212 | nurx | For Profit | 9 | 11.5 | 11.3 | 10.4 | 10.55 | https://www.nurx.com/faq/what-are-the-side-effects-of-truvada/ | https://web.archive.org/web/20220516012159/https://www.nurx.com/faq/what-are-the-side-effects-of-truvada/ | Truvada PrEP | Search Engine | Oral | General | Website |
| 213 | NYC Health | The US Government | 12 | 14.5 | 13.87 | 14.4 | 13.6925 | https://home3.nyc.gov/site/doh/providers/health-topics/pre-exposure-prophylaxis-provider-faq.page | https://web.archive.org/web/20220516012211/https://home3.nyc.gov/site/doh/providers/health-topics/pre-exposure-prophylaxis-provider-faq.page | PrEP | Search Engine | Oral | Provider | Website |
| 214 | Oklahoma Government | The US Government | 13.3 | 14.6 | 13.35 | 15.5 | 14.1875 | https://www.ok.gov/health2/documents/PrEP%20Your%20Provider%20Quick-start%20Brochure.pdf | https://web.archive.org/web/20220516012503/https://www.ok.gov/health2/documents/PrEP%20Your%20Provider%20Quick-start%20Brochure.pdf | PrEP | Search Engine | Oral | Provider | Brochure |
| 215 | Planned Parenthood | Non Government Organization | 5.6 | 9.7 | 7.65 | 7.5 | 7.6125 | https://www.plannedparenthood.org/learn/stds-hiv-safer-sex/hiv-aids/prep | https://web.archive.org/web/20220516012510/https://www.plannedparenthood.org/learn/stds-hiv-safer-sex/hiv-aids/prep | PrEP | Search Engine | Oral | General | Website |
| 216 | Plush Care | For Profit | 7.8 | 11.2 | 10.08 | 9.2 | 9.57 | https://plushcare.com/prep-online/#:~:text=Commonly%20reported%20PrEP%20side%20effects%20include%3A%201,Dizziness%202%20Vomiting%203%20Fatigue%204%20Nausea | https://web.archive.org/web/20220516012521/https://plushcare.com/prep-online/ | PrEP | Search Engine | General PrEP | General | Website |
| 217 | PrEP Daily - The Health Foundation of Greater Indianapolis | Non Government Organization | 8.7 | 11.7 | 10.21 | 10.1 | 10.1775 | https://prepdaily.org/how-does-prep-prevent-hiv-transmission/ | https://web.archive.org/web/20220516012524/https://prepdaily.org/how-does-prep-prevent-hiv-transmission/ | PrEP | Search Engine | General PrEP | General | Website |
| 218 | PrEP Daily - The Health Foundation of Greater Indianapolis | Non Government Organization | 9.7 | 12.4 | 9.57 | 10.6 | 10.5675 | https://prepdaily.org/how-much-does-prep-cost/ | https://web.archive.org/web/20220516012535/https://prepdaily.org/how-much-does-prep-cost/ | PrEP | Search Engine | General PrEP | General | Website |
| 219 | PrEP Daily - The Health Foundation of Greater Indianapolis | Non Government Organization | 10.1 | 13 | 10.15 | 11.6 | 11.2125 | https://prepdaily.org/tops-bottoms-and-prep-what-you-need-to-know-about-hiv-prevention/ | https://web.archive.org/web/20220516012648/https://prepdaily.org/tops-bottoms-and-prep-what-you-need-to-know-about-hiv-prevention/ | PrEP | Search Engine | General PrEP | General | Website |
| 220 | PrEP Daily - The Health Foundation of Greater Indianapolis | Non Government Organization | 10.8 | 13.1 | 12.3 | 12.8 | 12.25 | https://prepdaily.org/what-are-the-side-effects-of-prep/ | https://web.archive.org/web/20220516012655/https://prepdaily.org/what-are-the-side-effects-of-prep/ | PrEP | Search Engine | Oral | General | Website |
| 221 | PrEP.Health | For Profit | 7.7 | 11.2 | 9.56 | 8.7 | 9.29 | https://prep.health/frequently-asked-questions-faq-prep-health/ | https://web.archive.org/web/20220516012711/https://prep.health/frequently-asked-questions-faq-prep-health/ | PrEP | Search Engine | Oral | General | Website |
| 222 | prep.scot - NHS | Non-US Government/other Public Health Organization | 6.5 | 9.6 | 7.6 | 8.1 | 7.95 | https://www.prep.scot/wp-content/uploads/2022/05/PrEP-booklet-generic-220419-web.pdf | https://web.archive.org/web/20220516012708/https://www.prep.scot/wp-content/uploads/2022/05/PrEP-booklet-generic-220419-web.pdf | PrEP | Search Engine | Oral | General | Brochure |
| 223 | PrEPAccessNow | Non Government Organization | 7.6 | 10.4 | 9.1 | 9.3 | 9.1 | https://www.pan.org.au/resources/brochures. https://www.pan.org.au/blog/2021/9/10/prep-hiv-and-the-covid-vaccine | https://web.archive.org/web/20220516012744/https://www.pan.org.au/blog/2021/9/10/prep-hiv-and-the-covid-vaccine | PrEP | Search Engine | General PrEP | General | Brochure |
| 224 | PrEPAccessNow | Non Government Organization | 6.3 | 9.7 | 7.76 | 6.9 | 7.665 | https://www.pan.org.au/blog/2018/11/23/descovy-as-prep | https://web.archive.org/web/20220516012725/https://www.pan.org.au/blog/2018/11/23/descovy-as-prep | DESCOVY - PrEP | Search Engine | Oral | General | Website |
| 225 | PrEPAccessNow | Non Government Organization | 7.1 | 9.3 | 6.73 | 8 | 7.7825 | https://www.pan.org.au/blog/2018/11/23/self-testing-on-prep | https://web.archive.org/web/20220516012841/https://www.pan.org.au/blog/2018/11/23/self-testing-on-prep | PrEP | Search Engine | General PrEP | General | Website |
| 226 | PrEPAccessNow | Non Government Organization | 7.7 | 10.9 | 8.94 | 10.6 | 9.535 | https://www.pan.org.au/blog/2019/2/5/what-if-my-prep-is-a-different-colour | https://web.archive.org/web/20220516012848/https://www.pan.org.au/blog/2019/2/5/what-if-my-prep-is-a-different-colour | PrEP | Search Engine | Oral | General | Website |
| 227 | PrEPAccessNow | Non Government Organization | 6.4 | 9.3 | 6.84 | 7.5 | 7.51 | https://www.pan.org.au/blog/2018/11/28/do-i-need-to-take-prep-every-day | https://web.archive.org/web/20220516012854/https://www.pan.org.au/blog/2018/11/28/do-i-need-to-take-prep-every-day | PrEP | Search Engine | Oral | General | Website |
| 228 | PrEPAccessNow | Non Government Organization | 9.8 | 12 | 11.71 | 11.5 | 11.2525 | https://www.pan.org.au/blog/2018/11/26/can-i-take-viagra-if-i-am-on-prep | https://web.archive.org/web/20220516012859/https://www.pan.org.au/blog/2018/11/26/can-i-take-viagra-if-i-am-on-prep | PrEP | Search Engine | Oral | General | Website |
| 229 | PrEPAccessNow | Non Government Organization | 8.9 | 11.4 | 9.34 | 9.7 | 9.835 | https://www.pan.org.au/blog/2018/11/23/damaged-prep | https://web.archive.org/web/20220516012904/https://www.pan.org.au/blog/2018/11/23/damaged-prep | PrEP | Search Engine | Oral | General | Website |
| 230 | PrEPAccessNow | Non Government Organization | 7.7 | 11.1 | 8.58 | 8.9 | 9.07 | https://www.pan.org.au/blog/2018/11/12/can-i-stop-using-condoms | https://web.archive.org/web/20220516012911/https://www.pan.org.au/blog/2018/11/12/can-i-stop-using-condoms | PrEP | Search Engine | General PrEP | General | Website |
| 231 | PrEPAccessNow | Non Government Organization | 7.2 | 10.8 | 7.48 | 7.6 | 8.27 | https://www.pan.org.au/blog/2018/11/12/how-effective-is-prep | https://web.archive.org/web/20220516013014/https://www.pan.org.au/blog/2018/11/12/how-effective-is-prep | PrEP | Search Engine | Oral | General | Website |
| 232 | PrEPAccessNow | Non Government Organization | 9.3 | 10.9 | 8.94 | 11 | 10.035 | https://www.pan.org.au/blog/2018/11/12/what-if-my-doctor-doesnt-know-about-prep | https://web.archive.org/web/20220516013021/https://www.pan.org.au/blog/2018/11/12/what-if-my-doctor-doesnt-know-about-prep | PrEP | Search Engine | General PrEP | General | Website |
| 233 | PrEPAccessNow | Non Government Organization | 8.2 | 10 | 9.46 | 10.1 | 9.44 | https://www.pan.org.au/blog/2018/11/8/does-generic-prep-work-the-same-as-brand-name-truvada | https://web.archive.org/web/20220516013026/https://www.pan.org.au/blog/2018/11/8/does-generic-prep-work-the-same-as-brand-name-truvada | PrEP | Search Engine | Oral | General | Website |
| 234 | PrEPAccessNow | Non Government Organization | 8.1 | 10.9 | 9.52 | 10.1 | 9.655 | https://www.pan.org.au/tasp-faqs | https://web.archive.org/web/20220516013040/https://www.pan.org.au/tasp-faqs | HIV | Search Engine | General PrEP | General | Website |
| 235 | PrEPAccessNow | Non Government Organization | 7.6 | 10.5 | 9.05 | 9.3 | 9.1125 | https://www.pan.org.au/prep-101 | https://web.archive.org/web/20220516014600/https://www.pan.org.au/prep-101 | PrEP | Search Engine | Oral | General | Website |
| 236 | PrEPAccessNow | Non Government Organization | 6 | 9.3 | 7.19 | 7.4 | 7.4725 | https://www.pan.org.au/prep-faqs | https://web.archive.org/web/20220516014608/https://www.pan.org.au/prep-faqs | PrEP | Search Engine | General PrEP | General | Website |
| 237 | PrEPglobal | Non Government Organization | 6.3 | 9.5 | 7.54 | 7.9 | 7.81 | https://www.prep.global/prep-facts. https://www.prep.global/faq | https://web.archive.org/web/20220516014615/https://www.prep.global/prep-facts.-https%3A/www.prep.global/faq | PrEP | Search Engine | Oral | General | Website |
| 238 | prepwatch, AVAC - Global Advocacy for HIV Prevention | Non Government Organization | 6.2 | 10.3 | 7.88 | 6.8 | 7.795 | http://www.prepwatch.org/wp-content/uploads/2016/08/z-fold_FAQbrochurePrEPsa.pdf | N/A | PrEP | Search Engine | Oral | General | Brochure |
| 239 | prepwatch, AVAC - Global Advocacy for HIV Prevention | Non Government Organization | 5 | 8.8 | 7.24 | 6.3 | 6.835 | https://www.prepwatch.org/wp-content/uploads/2018/01/PrEP_PalmCard.pdf | https://web.archive.org/web/20220516014628/https://www.prepwatch.org/wp-content/uploads/2018/01/PrEP_PalmCard.pdf | PrEP | Search Engine | Oral | General | Brochure |
| 240 | Public Health Institute at Denver Health | Non Government Organization | 11.2 | 12.9 | 11.89 | 13.1 | 12.2725 | https://www.phidenverhealth.org/clinics-services/hiv/resources-education/prep | https://web.archive.org/web/20220516015704/https://www.phidenverhealth.org/clinics-services/hiv/resources-education/prep | PrEP | Search Engine | Oral | General | Website |
| 241 | Public Health Institute at Denver Health | Non Government Organization | 8.6 | 11.4 | 10.66 | 9.3 | 9.99 | https://www.phidenverhealth.org/clinics-services/hiv/resources-education/pep | https://web.archive.org/web/20220516015712/https://www.phidenverhealth.org/clinics-services/hiv/resources-education/pep | PrEP | Search Engine | Oral | General | Website |
| 242 | Public Health Institute at Denver Health | Non Government Organization | 7.6 | 10.5 | 10.14 | 9 | 9.31 | https://www.phidenverhealth.org/clinics-services/hiv/resources-education/hiv-facts | https://web.archive.org/web/20220516015721/https://www.phidenverhealth.org/clinics-services/hiv/resources-education/hiv-facts | PrEP | Search Engine | General PrEP | General | Website |
| 243 | Qcare+ | For Profit | 9.6 | 12.3 | 12.12 | 11.6 | 11.405 | https://qcareplus.com/?gclid=Cj0KCQjwma6TBhDIARIsAOKuANzcOEbGYCScSTQFrSFxOeIkI1YNyxDKst1ob3bnewkpr_TSCkoWquIaAt4tEALw_wcB. https://qcareplus.com/learn-about-prep/ | https://web.archive.org/web/20220516015904/https://qcareplus.com/learn-about-prep/ | PrEP | Search Engine | Oral | General | Website |
| 244 | RUSH | For Profit | 9.2 | 12.3 | 10.5 | 11.1 | 10.775 | https://www.rush.edu/news/6-facts-about-prep | https://web.archive.org/save/https://www.rush.edu/news/6-facts-about-prep | PrEP | Search Engine | Oral | General | Website |
| 245 | sexualwellbeing - HSE | Non-US Government/other Public Health Organization | 7 | 10.3 | 7.43 | 8.4 | 8.2825 | https://www.sexualwellbeing.ie/sexual-health/prep/how-to-take-prep/ | https://web.archive.org/web/20220516020120/https://www.sexualwellbeing.ie/sexual-health/prep/how-to-take-prep/ | PrEP | Search Engine | Oral | General | Website |
| 246 | Society of Behavioral Medicine | Non Government Organization | 7 | 10.1 | 8.41 | 8 | 8.3775 | https://www.sbm.org/healthy-living/prep-facts--hiv-prevention | https://web.archive.org/web/20220516020125/https://www.sbm.org/healthy-living/prep-facts--hiv-prevention | PrEP | Search Engine | Oral | General | Website |
| 247 | Stanford HEALTH CARE | For Profit | 9.4 | 11.8 | 10.73 | 10 | 10.4825 | https://stanfordhealthcare.org/medical-conditions/sexual-and-reproductive-health/hiv-aids/treatments/prep.html | https://web.archive.org/web/20220516020152/https://stanfordhealthcare.org/medical-conditions/sexual-and-reproductive-health/hiv-aids/treatments/prep.html | PrEP | Search Engine | Oral | General | Website |
| 248 | Teva Pharmaceuticals Inc. | For Profit | 8.5 | 10.4 | 12.76 | 12.2 | 10.965 | https://www.tevahivgenerics.com/truvada-generic. https://www.tevahivgenerics.com/Truvada-generic/patient-faqs | https://web.archive.org/web/20220516020456/https://www.tevahivgenerics.com/Truvada-generic/patient-faqs | Truvada PrEP | Search Engine | Oral | General | Website |
| 249 | Teva Pharmaceuticals Inc. | For Profit | 21.2 | 19.2 | 17.24 | 26.6 | 21.06 | https://www.tevahivgenerics.com/truvada-generic/dosing-and-administration | https://web.archive.org/web/20220516020606/https://www.tevahivgenerics.com/truvada-generic/dosing-and-administration | Truvada PrEP | Search Engine | Oral | General | Website |
| 250 | TheBody | N/A | 11.5 | 13.5 | 11.96 | 15.2 | 13.04 | https://www.thebody.com/article/what-does-prep-cost-outside-the-united-states. | https://web.archive.org/web/20220516020612/https://www.thebody.com/article/what-does-prep-cost-outside-the-united-states. | PrEP | Search Engine | Oral | General | Website |
| 251 | TheBody | N/A | 8.3 | 11.3 | 9.28 | 10.2 | 9.77 | https://www.thebody.com/health/hiv-prep-pre-exposure-prophylaxis | https://web.archive.org/web/20220516020623/https://www.thebody.com/health/hiv-prep-pre-exposure-prophylaxis | PrEP | Search Engine | Oral | General | Website |
| 252 | Gilead Sciences | For Profit | 8.9 | 11.5 | 10.09 | 10.3 | 10.1975 | https://services.gileadhiv.com/content/pdf/truvadaprep/truvadaprep-brochure.pdf | https://web.archive.org/web/20220516020818/https://services.gileadhiv.com/content/pdf/truvadaprep/truvadaprep-brochure.pdf | Truvada - PrEP | Search Engine | Oral | General | Brochure |
| 253 | FDA - U.S. Food and Drug Administration | The US Government | 17 | 18 | 13.88 | 18.5 | 16.845 | https://www.fda.gov/media/83586/download | https://web.archive.org/web/20220516020841/https://www.fda.gov/media/83586/download | PrEP | Search Engine | Oral | General | Information Sheet |
| 254 | Centers for Disease Control and Prevention | The US Government | 5.9 | 9 | 7.35 | 6.3 | 7.1375 | https://www.vdh.virginia.gov/content/uploads/sites/10/2016/02/CDC_PrEP_Brochure.pdf | N/A | PrEP | Search Engine | General PrEP | General | Brochure |
| 255 | U.S. Department of Veterans Affairs | The US Government | 9.4 | 12.8 | 10.73 | 11.4 | 11.0825 | https://www.hiv.va.gov/patient/faqs/preexposure-prophylaxis.asp | https://web.archive.org/web/20220516020900/https://www.hiv.va.gov/patient/faqs/preexposure-prophylaxis.asp | PrEP | Search Engine | General PrEP | General | Website |
| 256 | U.S. President‚Äôs Emergency Plan for AIDS Relief (PEPFAR) and the U.S. Agency for International Development (USAID) | The US Government | 5.5 | 9.5 | 6.31 | 6.4 | 6.9275 | https://publications.jsi.com/JSIInternet/Inc/Common/_download_pub.cfm?id=23834&lid=3 | https://web.archive.org/web/20220516021015/https://publications.jsi.com/JSIInternet/Inc/Common/_download_pub.cfm?id=23834&lid=3 | PrEP | Search Engine | Oral | General | Information Sheet |
| 257 | U.S. President‚Äôs Emergency Plan for AIDS Relief (PEPFAR) and the U.S. Agency for International Development (USAID) | The US Government | 7.3 | 10.4 | 8.92 | 7.7 | 8.58 | https://www.pedaids.org/wp-content/uploads/2019/02/2018PrEPBrochure_11.27.pdf | N/A | PrEP | Search Engine | Oral | General | Brochure |
| 258 | verywellhealth | Non Government Organization | 12.7 | 14.2 | 12.07 | 14.8 | 13.4425 | https://www.verywellhealth.com/how-effective-is-prep-in-preventing-hiv-4010575 | https://web.archive.org/web/20220516021031/https://www.verywellhealth.com/how-effective-is-prep-in-preventing-hiv-4010575 | HIV-AIDS | Search Engine | General PrEP | General | Website |
| 259 | Washington Health Institue | The US Government | 9.1 | 11.8 | 11.36 | 10.4 | 10.665 | https://dc-whi.org/what-is-prep-and-how-does-it-work/ | https://web.archive.org/web/20220516021037/https://dc-whi.org/what-is-prep-and-how-does-it-work/ | PrEP | Search Engine | Oral | General | Website |
| 260 | Washington State Department of Health | The US Government | 15 | 15.5 | 11.79 | 17.4 | 14.9225 | https://doh.wa.gov/sites/default/files/legacy/Documents/Pubs//150-055-PrEPDAPBrochure.pdf | https://web.archive.org/web/20220516021133/https://doh.wa.gov/sites/default/files/legacy/Documents/Pubs//150-055-PrEPDAPBrochure.pdf | PrEP | Search Engine | Oral | General | Brochure |
| 261 | WebMD | For Profit | 9.1 | 12 | 10.5 | 11.3 | 10.725 | https://www.webmd.com/hiv-aids/how-much-truvada-for-prep-costs | https://web.archive.org/web/20220516021140/https://www.webmd.com/hiv-aids/how-much-truvada-for-prep-costs | Truvada PrEP | Search Engine | Oral | General | Website |
| 262 | WebMD | For Profit | 7.3 | 10.2 | 8.76 | 9 | 8.815 | https://www.webmd.com/hiv-aids/prep-faq-preexposure-prophylaxis | https://web.archive.org/web/20220516021151/https://www.webmd.com/hiv-aids/prep-faq-preexposure-prophylaxis | PrEP | Search Engine | Oral & Injection PrEP | General | Website |
| 263 | WebMD | For Profit | 6.7 | 9.6 | 8.23 | 7.9 | 8.1075 | https://www.webmd.com/hiv-aids/prep-for-hiv-and-aids-how-effective | https://web.archive.org/web/20220516021210/https://www.webmd.com/hiv-aids/prep-for-hiv-and-aids-how-effective | PrEP | Search Engine | General PrEP | General | Website |
| 264 | WHAT IS TRUVADA | N/A | 7 | 11.3 | 8.7 | 8.2 | 8.8 | https://whatistruvada.com/ | https://web.archive.org/web/20220516021218/https://whatistruvada.com/ | Truvada PrEP | Search Engine | Oral | General | Website |
| 265 | WHAT IS TRUVADA | N/A | 7.1 | 10 | 9.09 | 7.7 | 8.4725 | https://whatistruvada.com/about/ | https://web.archive.org/web/20220516021305/https://whatistruvada.com/about/ | Truvada PrEP | Search Engine | Oral | General | Website |
| 266 | WHAT IS TRUVADA | N/A | 8 | 11.2 | 10.25 | 8.3 | 9.4375 | https://whatistruvada.com/ways-to-prep/ | https://web.archive.org/web/20220516021310/https://whatistruvada.com/ways-to-prep/ | Truvada PrEP | Search Engine | Oral | General | Website |
| 267 | whatisprep | Non Government Organization | 8.4 | 11.4 | 7.54 | 8.7 | 9.01 | http://www.whatisprep.org/ | https://web.archive.org/web/20220516021314/http://www.whatisprep.org/ | PrEP | Search Engine | General PrEP | General | Website |
| 268 | Wisconsin Department of Health Services | The US Government | 12 | 14.1 | 13.23 | 15.3 | 13.6575 | https://www.dhs.wisconsin.gov/hiv/prep-defined.htm | https://web.archive.org/web/20220516021320/https://www.dhs.wisconsin.gov/hiv/prep-defined.htm | PrEP | Search Engine | Oral | General | Website |
| 269 | Wisconsin Department of Health Services | The US Government | 7.4 | 10.2 | 8.18 | 8.3 | 8.52 | https://www.dhs.wisconsin.gov/hiv/prep.htm. https://www.dhs.wisconsin.gov/hiv/prep-who.htm | https://web.archive.org/web/20220516021327/https://www.dhs.wisconsin.gov/hiv/prep-who.htm | PrEP | Search Engine | Oral | General | Website |
| 270 | Wisconsin Department of Health Services | The US Government | 11.7 | 14.9 | 12.18 | 14.1 | 13.22 | https://www.dhs.wisconsin.gov/hiv/prep-where.htm | https://web.archive.org/web/20220516021423/https://www.dhs.wisconsin.gov/hiv/prep-where.htm | PrEP | Search Engine | General PrEP | General | Website |
| 271 | afao - Australian Federation of AIDS Organizations | Non Government Organization | 8.4 | 11.1 | 9.51 | 9.3 | 9.5775 | https://www.afao.org.au/wp-content/uploads/2018/03/2335_afao_prep_fact_sheet_11-1.pdf | N/A | PrEP | Twitter | General PrEP | General | Information Sheet |
| 272 | afao - Australian Federation of AIDS Organizations | Non Government Organization | 5.6 | 9.4 | 6.95 | 7 | 7.2375 | https://www.afao.org.au/wp-content/uploads/2021/06/2991_afao_hivfactsheets_prep_art.pdf | N/A | PrEP | Twitter | Oral | General | Brochure |
| 273 | AID ATLANTA | Non Government Organization | 12.3 | 13.3 | 10.8 | 14.6 | 12.75 | https://www.aidatlanta.org/prep/ | https://web.archive.org/web/20220423205939/https://www.aidatlanta.org/prep/ | PrEP | Twitter | Oral | General | Website |
| 274 | aidsmap | Non Government Organization | 9.2 | 11.6 | 11.25 | 11.7 | 10.9375 | https://www.aidsmap.com/about-hiv/arv-factsheet/cabotegravir-and-rilpivirine-injections | https://web.archive.org/web/20220420225129/https://www.aidsmap.com/about-hiv/arv-factsheet/cabotegravir-and-rilpivirine-injections | HIV, Cabotegravir | Twitter | Injection | General | Website |
| 275 | aidsmap | Non Government Organization | 10.5 | 12.6 | 13.46 | 13.5 | 12.515 | https://www.aidsmap.com/about-hiv/arv-factsheet/emtricitabinetenofovir-disoproxil-fumarate | https://web.archive.org/web/20220423210738/https://www.aidsmap.com/about-hiv/arv-factsheet/emtricitabinetenofovir-disoproxil-fumarate | HIV | Twitter | Oral | General | Website |
| 276 | aidsmap | Non Government Organization | 10.4 | 12.5 | 12.41 | 12.6 | 11.9775 | https://www.aidsmap.com/about-hiv/what-do-we-know-about-injectable-hiv-medication | https://web.archive.org/web/20220425191305/https://www.aidsmap.com/about-hiv/what-do-we-know-about-injectable-hiv-medication | HIV | Twitter | Injection | General | Website |
| 277 | amfAR | For Profit | 8.9 | 11.8 | 10.26 | 10.7 | 10.415 | https://archive.amfar.org/About-HIV-and-AIDS/Young-People-and-HIV/Young-People-and-HIV-AIDS/ | https://web.archive.org/web/20220425191319/https://archive.amfar.org/About-HIV-and-AIDS/Young-People-and-HIV/Young-People-and-HIV-AIDS/ | HIV-AIDS | Twitter | General PrEP | General | Website |
| 278 | amfAR | For Profit | 8.4 | 11.8 | 11.48 | 11 | 10.67 | https://www.amfar.org/About-HIV-and-AIDS/Basic-Facts-About-HIV/ | https://web.archive.org/web/20220421145033/https://www.amfar.org/about-hiv-aids/basic-facts-about-hiv-aids/ | HIV-AIDS | Twitter | General PrEP | General | Website |
| 279 | AVAC - Global Advocacy for HIV Prevention | Non Government Organization | 8.6 | 11.6 | 10.84 | 10.7 | 10.435 | https://www.avac.org/sites/default/files/resource-files/PrEP_Factsheet_Nov2020.pdf | N/A | PrEP | Twitter | Oral & Injection PrEP | General | Information Sheet |
| 280 | BE iN THE KNOW | For Profit | 8.4 | 10.3 | 9.28 | 9.1 | 9.27 | https://www.beintheknow.org/hiv-and-stis/hiv-prevention/how-can-i-protect-myself-and-others-hiv | https://web.archive.org/web/20220425191330/https://www.beintheknow.org/hiv-and-stis/hiv-prevention/how-can-i-protect-myself-and-others-hiv | HIV | Twitter | General PrEP | General | Website |
| 281 | BE iN THE KNOW | For Profit | 8.2 | 10.7 | 9.45 | 9.2 | 9.3875 | https://www.beintheknow.org/hiv-and-stis/hiv-prevention/pre-exposure-prophylaxis-prep | https://web.archive.org/web/20220425192241/https://www.beintheknow.org/hiv-and-stis/hiv-prevention/pre-exposure-prophylaxis-prep | PrEP | Twitter | General PrEP | General | Website |
| 282 | BE iN THE KNOW | For Profit | 6.3 | 8.9 | 7.13 | 6.4 | 7.1825 | https://www.beintheknow.org/hiv-and-stis/hiv-prevention/sex-and-hiv | https://web.archive.org/web/20220425192251/https://www.beintheknow.org/hiv-and-stis/hiv-prevention/sex-and-hiv | HIV | Twitter | General PrEP | General | Website |
| 283 | BE iN THE KNOW | For Profit | 6 | 9.2 | 7.53 | 6.7 | 7.3575 | https://www.beintheknow.org/hiv-and-stis/hiv-prevention/using-condoms-prevent-hiv | https://web.archive.org/web/20220425192258/https://www.beintheknow.org/hiv-and-stis/hiv-prevention/using-condoms-prevent-hiv | HIV | Twitter | General PrEP | General | Website |
| 284 | BE iN THE KNOW | For Profit | 7 | 10.8 | 9.21 | 7.6 | 8.6525 | https://www.beintheknow.org/hiv-and-stis/hiv-prevention/voluntary-medical-male-circumcision-vmmc | https://web.archive.org/web/20220425192308/https://www.beintheknow.org/hiv-and-stis/hiv-prevention/voluntary-medical-male-circumcision-vmmc | HIV | Twitter | General PrEP | General | Website |
| 285 | BE iN THE KNOW | For Profit | 5.4 | 8.9 | 6.94 | 5.6 | 6.71 | https://www.beintheknow.org/hiv-and-stis/understanding-hiv-and-aids/what-are-hiv-and-aids | https://web.archive.org/web/20220425192723/https://www.beintheknow.org/hiv-and-stis/understanding-hiv-and-aids/what-are-hiv-and-aids | HIV | Twitter | General PrEP | General | Website |
| 286 | BE iN THE KNOW | For Profit | 4.9 | 9.1 | 6.37 | 5.6 | 6.4925 | https://www.beintheknow.org/living-hiv/hiv-sex-and-relationships/i-have-hiv-can-i-have-sex | https://web.archive.org/web/20220425193008/https://www.beintheknow.org/living-hiv/hiv-sex-and-relationships/i-have-hiv-can-i-have-sex | HIV | Twitter | General PrEP | General | Website |
| 287 | BE iN THE KNOW | For Profit | 5.4 | 9.3 | 8.45 | 6.8 | 7.4875 | https://www.beintheknow.org/living-hiv/hiv-treatment/undetectable | https://web.archive.org/web/20220425192109/https://www.beintheknow.org/living-hiv/hiv-treatment/undetectable | HIV | Twitter | General PrEP | General | Website |
| 288 | BETTER TO KNOW | Non Government Organization | 7.5 | 9.4 | 7.65 | 8.1 | 8.1625 | https://www.bettertoknow.org.au/list-of-stis/hiv/ | https://web.archive.org/web/20220420231701/https://www.bettertoknow.org.au/list-of-stis/hiv/ | HIV | Twitter | General PrEP | General | Website |
| 289 | Birmingham LGBT | For Profit | 8.7 | 11.5 | 8.94 | 10.3 | 9.86 | https://blgbt.org/services/sexual-health/get-tested-2/prep/ | https://web.archive.org/web/20220421212557/https://blgbt.org/services/sexual-health/get-tested-2/prep/ | PrEP | Twitter | Oral | General | Website |
| 290 | Black PrEP | For Profit | 7.3 | 10.3 | 7.83 | 8 | 8.3575 | https://www.blackprep.org/prep-faq.html | N/A | PrEP | Twitter | Oral | General | Website |
| 291 | CATIE - Canada's source for HIV and hepatitis C information | For Profit | 8 | 10.3 | 8 | 8.8 | 8.775 | https://orders.catie.ca/book/prep-to-prevent-hiv-your-questions-answered/?utm_source=fb&utm_medium=socmed&utm_campaign=022719&utm_content=en | https://web.archive.org/web/20220422000705/https://orders.catie.ca/book/prep-to-prevent-hiv-your-questions-answered/ | HIV | Twitter | Oral | General | Brochure |
| 292 | CATIE - Canada's source for HIV and hepatitis C information | For Profit | 8.5 | 11.2 | 9.92 | 10.9 | 10.13 | https://www.catie.ca/client-publication/prep-q-a | https://web.archive.org/web/20220425195506/https://www.catie.ca/client-publication/prep-q-a | HIV | Twitter | Oral | General | Brochure |
| 293 | CATIE - Canada's source for HIV and hepatitis C information | For Profit | 7.5 | 9.2 | 8.58 | 8.9 | 8.545 | https://www.catie.ca/essentials/hiv-basics | https://web.archive.org/web/20220420225703/https://www.catie.ca/essentials/hiv-basics | HIV | Twitter | General PrEP | General | Website |
| 294 | CATIE - Canada's source for HIV and hepatitis C information | For Profit | 11.1 | 12.3 | 10.91 | 13.4 | 11.9275 | https://www.catie.ca/hiv-transmission | https://web.archive.org/web/20220421171151/https://www.catie.ca/hiv-transmission | HIV | Twitter | General PrEP | General | Website |
| 295 | CATIE - Canada's source for HIV and hepatitis C information | For Profit | 7.4 | 10 | 8.12 | 8.2 | 8.43 | https://www.catie.ca/prep | https://web.archive.org/web/20220425195515/https://www.catie.ca/prep | PrEP | Twitter | Oral | General | Website |
| 296 | Centers for Disease Control and Prevention | The US Government | 9.1 | 11.7 | 9.45 | 10.1 | 10.0875 | https://gettested.cdc.gov/faq-page | https://web.archive.org/web/20220425195559/https://gettested.cdc.gov/faq-page | STIs & HIV | Twitter | General PrEP | General | Website |
| 297 | Centers for Disease Control and Prevention | The US Government | 9.1 | 11.1 | 9.57 | 11.5 | 10.3175 | https://hivrisk.cdc.gov/can-increase-hiv-risk/ | https://web.archive.org/web/20220428020653/https://hivrisk.cdc.gov/can-increase-hiv-risk/ | STIs & HIV | Twitter | General PrEP | General | Website |
| 298 | Centers for Disease Control and Prevention | The US Government | 9 | 11.7 | 10.15 | 11.8 | 10.6625 | https://hivrisk.cdc.gov/can-decrease-hiv-risk/ | https://web.archive.org/web/20220428020602/https://hivrisk.cdc.gov/can-decrease-hiv-risk/ | STIs & HIV | Twitter | General PrEP | General | Website |
| 299 | Centers for Disease Control and Prevention | The US Government | 7.2 | 10.4 | 9.73 | 8.4 | 8.9325 | https://www.cdc.gov/hiv/basics/hiv-prevention/other-methods.html | https://web.archive.org/web/20220425200101/https://www.cdc.gov/hiv/basics/hiv-prevention/other-methods.html | HIV | Twitter | General PrEP | General | Website |
| 300 | Centers for Disease Control and Prevention | The US Government | 9.4 | 10.8 | 10.73 | 11.3 | 10.5575 | https://www.cdc.gov/hiv/basics/livingwithhiv/protecting-others.html | N/A | HIV | Twitter | General PrEP | General | Website |
| 301 | Centers for Disease Control and Prevention | The US Government | 7.8 | 10.4 | 9.05 | 9.6 | 9.2125 | https://www.cdc.gov/hiv/basics/hiv-prevention/protect-yourself-during-sex.html | https://web.archive.org/web/20220426202507/https://www.cdc.gov/hiv/basics/hiv-prevention/protect-yourself-during-sex.html | STIs & HIV | Twitter | General PrEP | General | Website |
| 302 | Centers for Disease Control and Prevention | The US Government | 6.4 | 10.3 | 9.16 | 8.3 | 8.54 | https://www.cdc.gov/hiv/pdf/library/consumer-info-sheets/cdc-hiv-consumer-info-sheet-prep-101.pdf#page=1 | N/A | PrEP | Twitter | General PrEP | General | Brochure |
| 303 | DAP Health | For Profit | 7 | 11.3 | 8.93 | 8.6 | 8.9575 | https://www.daphealth.org/answered-your-most-commonly-asked-questions-about-prep-for-hiv-prevention/ | https://web.archive.org/web/20220421171804/https://www.daphealth.org/answered-your-most-commonly-asked-questions-about-prep-for-hiv-prevention/ | HIV and PrEP | Twitter | General PrEP | General | Website |
| 304 | DAP Health | For Profit | 8.9 | 11.1 | 9.74 | 10.3 | 10.01 | https://www.daphealth.org/article/hiv/ | https://web.archive.org/web/20220425203846/https://www.daphealth.org/article/hiv/ | HIV | Twitter | General PrEP | General | Website |
| 305 | debt free guys | Non Government Organization | 10.4 | 13.1 | 12.36 | 13.8 | 12.415 | https://debtfreeguys.com/questions-about-prep-answered/ | https://web.archive.org/web/20220421171445/https://debtfreeguys.com/questions-about-prep-answered/ | PrEP | Twitter | General PrEP | General | Website |
| 306 | DESMOND TUTU HEALTH FOUNDATION | Non Government Organization | 9.4 | 12.5 | 10.56 | 11.2 | 10.915 | https://desmondtutuhealthfoundation.org.za/blog_post/prep-your-guide-to-this-hiv-prevention-drug/ | https://web.archive.org/web/20220420015139/https://desmondtutuhealthfoundation.org.za/blog_post/prep-your-guide-to-this-hiv-prevention-drug/ | PrEP | Twitter | General PrEP | General | Website |
| 307 | dtap | For Profit | 8.2 | 11.7 | 10.26 | 9.7 | 9.965 | https://dtapclinic.com.my/hiv-pre-exposure-prophylaxis-prep/ | https://web.archive.org/web/20220425203930/https://dtapclinic.com.my/hiv-pre-exposure-prophylaxis-prep/ | PrEP | Twitter | Oral | General | Website |
| 308 | dtap | For Profit | 10.5 | 12.1 | 9.28 | 11.6 | 10.87 | https://www.dtapclinic.com/articles/7-faqs-hiv-pre-exposure-prophylaxis-hiv-prep/ | https://web.archive.org/web/20220425203958/https://www.dtapclinic.com/articles/7-faqs-hiv-pre-exposure-prophylaxis-hiv-prep/ | PrEP | Twitter | Oral | General | Website |
| 309 | dtap | For Profit | 8.7 | 11.9 | 8.82 | 10.3 | 9.93 | https://www.dtapclinic.com/articles/a-guide-to-hiv-prep-and-hiv-pep-pills-for-hiv-prevention/ | https://web.archive.org/web/20220420202337/https://www.dtapclinic.com/articles/a-guide-to-hiv-prep-and-hiv-pep-pills-for-hiv-prevention/ | HIV, PrEP & PEP | Twitter | General PrEP | General | Website |
| 310 | dtap | For Profit | 10.7 | 13.2 | 10.5 | 12.7 | 11.775 | https://www.dtapclinic.com/hiv/hiv-prep/#toggle-id-5 | https://web.archive.org/web/20220425204035/https://www.dtapclinic.com/hiv/hiv-prep/ | PrEP | Twitter | Oral | General | Website |
| 311 | ENDING HIV | Non Government Organization | 9.5 | 11.7 | 8.65 | 11.1 | 10.2375 | https://endinghiv.org.au/stay-safe/prep/ | https://web.archive.org/web/20220420231929/https://endinghiv.org.au/stay-safe/prep/ | HIV & PrEP | Twitter | Oral | General | Website |
| 312 | ENDING HIV TOGETHER - Marion County Public Health Department | Non Government Organization | 9.2 | 10.7 | 8.24 | 10.4 | 9.635 | https://endinghivtogether.org/hiv-101/ | https://web.archive.org/web/20220425205057/https://endinghivtogether.org/hiv-101/ | HIV | Twitter | General PrEP | General | Website |
| 313 | ENDING HIV TOGETHER - Marion County Public Health Department | Non Government Organization | 8.9 | 11.5 | 10.03 | 10.2 | 10.1575 | https://endinghivtogether.org/hiv-prevention-and-you-a-complete-guide/ | https://web.archive.org/web/20220425205118/https://endinghivtogether.org/hiv-prevention-and-you-a-complete-guide/ | HIV, PrEP & PEP | Twitter | General PrEP | General | Website |
| 314 | ENDING HIV TOGETHER - Marion County Public Health Department | Non Government Organization | 5.4 | 8.9 | 7.4 | 5.9 | 6.9 | https://endinghivtogether.org/learn-about-prep/ | https://web.archive.org/web/20220425205109/https://endinghivtogether.org/learn-about-prep/ | PrEP | Twitter | General PrEP | General | Website |
| 315 | ENDING HIV TOGETHER - Marion County Public Health Department | Non Government Organization | 9.1 | 12.1 | 10.5 | 11 | 10.675 | https://endinghivtogether.org/youth-and-hiv-a-guide-for-parents/ | https://web.archive.org/web/20220425205159/https://endinghivtogether.org/youth-and-hiv-a-guide-for-parents/ | HIV | Twitter | General PrEP | General | Website |
| 316 | EVERYDAY HEALTH | Non Government Organization | 12.3 | 14 | 10.74 | 14.7 | 12.935 | https://www.everydayhealth.com/hiv-aids/faqs-for-when-your-partner-has-hiv/ | https://web.archive.org/web/20220420224859/https://www.everydayhealth.com/favicon.png? | HIV-AIDS | Twitter | General PrEP | General | Website |
| 317 | Gilead Sciences | For Profit | 8.5 | 11.4 | 10.61 | 10.5 | 10.2525 | https://services.gileadhiv.com/content/pdf/TRUVADA_for_PrEP_Important_Facts.pdf | N/A | PrEP | Twitter | Oral | General | Information Sheet |
| 318 | Gilead Sciences | For Profit | 6.7 | 10.3 | 9.56 | 9 | 8.89 | https://www.truvada.com/how-to-get-truvada-for-prep/talking-to-your-doctor | N/A | Truvada | Twitter | Oral | General | Website |
| 319 | Gilead Sciences | For Profit | 7 | 10.6 | 9.68 | 9.3 | 9.145 | https://www.truvada.com/is-truvada-right-for-me/understanding-hiv-risk | N/A | Truvada | Twitter | Oral | General | Website |
| 320 | GMHC | Non Government Organization | 12.3 | 14.1 | 11.84 | 13.4 | 12.91 | https://www.gmhc.org/resources/prep-pre-exposure-prophylaxis/ | https://web.archive.org/web/20220425205401/https://www.gmhc.org/resources/prep-pre-exposure-prophylaxis/ | PrEP | Twitter | General PrEP | General | Website |
| 321 | Greater Than AIDS (WE AIDS) | Non Government Organization | 8.3 | 11.1 | 10.15 | 9.7 | 9.8125 | https://www.greaterthan.org/hiv-faq/ | https://web.archive.org/web/20220425205438/https://www.greaterthan.org/hiv-faq/ | HIV | Twitter | General PrEP | General | Website |
| 322 | Greater Than AIDS (WE AIDS) | Non Government Organization | 8 | 11.3 | 9.97 | 9.5 | 9.6925 | https://www.greaterthan.org/prep-faq/ | https://web.archive.org/web/20220419215640/https://www.greaterthan.org/prep-faq/ | PrEP | Twitter | Oral | General | Website |
| 323 | Greater Than AIDS (WE AIDS) | Non Government Organization | 6.4 | 9.8 | 7.94 | 7.1 | 7.81 | https://www.greaterthan.org/test-faq/ | https://web.archive.org/web/20220425205602/https://www.greaterthan.org/test-faq/ | HIV | Twitter | General PrEP | General | Website |
| 324 | Health & Wellness Tips Guide | N/A | 8.2 | 10.1 | 8.47 | 9.3 | 9.0175 | https://wellnesstipsguide.health.blog/2019/12/04/hiv-prep-and-pep-health-tips-reviews/ | https://web.archive.org/web/20220420204737/https://wellnesstipsguide.health.blog/2019/12/04/hiv-prep-and-pep-health-tips-reviews/ | PrEP & PEP | Twitter | General PrEP | General | Website |
| 325 | HealthTap | For Profit | 11.1 | 13 | 13.46 | 14.8 | 13.09 | https://www.healthtap.com/questions/1271336-is-daily-truvada-emtricitabine-and-tenofovir-a-good-preventative-medication-for-hiv-what-are-my-r/?utm_content=bufferf1074&utm_medium=social&utm_source=twitter.com&utm_campaign=buffer | https://web.archive.org/web/20220420224401/https://www.healthtap.com/questions/1271336-is-daily-truvada-emtricitabine-and-tenofovir-a-good-preventative-medication-for-hiv-what-are-my-r/ | Truvada | Twitter | Oral | General | Website |
| 326 | HiM - Health Initaitive for Men | Non Government Organization | 7.9 | 11.6 | 9.1 | 9.8 | 9.6 | https://checkhimout.ca/gay-mens-health/hiv/hiv-detection-transmission/ | https://web.archive.org/web/20220425205554/https://checkhimout.ca/gay-mens-health/hiv/hiv-detection-transmission/ | HIV | Twitter | General PrEP | General | Website |
| 327 | HiM - Health Initaitive for Men | Non Government Organization | 8.7 | 12.1 | 8.88 | 10.5 | 10.045 | https://checkhimout.ca/pep/how-much-does-prep-cost/ | https://web.archive.org/web/20220425210511/https://checkhimout.ca/pep/how-much-does-prep-cost/ | PrEP | Twitter | General PrEP | General | Website |
| 328 | HiM - Health Initaitive for Men | Non Government Organization | 9.2 | 11.9 | 9.45 | 10.2 | 10.1875 | https://checkhimout.ca/what-is-prep/ | https://web.archive.org/web/20220425210533/https://checkhimout.ca/what-is-prep/ | PrEP | Twitter | Oral | General | Website |
| 329 | HiM - Health Initaitive for Men | Non Government Organization | 9.1 | 12.4 | 9.34 | 11.2 | 10.51 | https://checkhimout.ca/what-is-prep/how-effective-is-prep/ | https://web.archive.org/web/20220425210540/https://checkhimout.ca/what-is-prep/how-effective-is-prep/ | PrEP | Twitter | Oral | General | Website |
| 330 | HiM - Health Initaitive for Men | Non Government Organization | 12.2 | 14.2 | 9.01 | 14.5 | 12.4775 | https://checkhimout.ca/what-is-prep/is-prep-right-for-me/ | https://web.archive.org/web/20220425210636/https://checkhimout.ca/what-is-prep/is-prep-right-for-me/ | PrEP | Twitter | General PrEP | General | Website |
| 331 | HiM - Health Initaitive for Men | Non Government Organization | 7.8 | 11.2 | 8.93 | 9.5 | 9.3575 | https://checkhimout.ca/what-is-prep/is-prep-safe-to-take/ | https://web.archive.org/web/20220420225847/https://checkhimout.ca/what-is-prep/is-prep-safe-to-take/ | PrEP | Twitter | General PrEP | General | Website |
| 332 | HIV Media Guide | Non Government Organization | 13.6 | 15.9 | 13.18 | 15.2 | 14.47 | https://www.hivmediaguide.org.au/facts-about-hiv/hiv-prevention/safe-sex/index.html | N/A | STIs & HIV | Twitter | General PrEP | General | Website |
| 333 | HIV Media Guide | Non Government Organization | 14.8 | 16.4 | 12.25 | 16.2 | 14.9125 | https://www.hivmediaguide.org.au/facts-about-hiv/hiv-prevention/pre-exposure-prophylaxis/index.html | N/A | PrEP | Twitter | General PrEP | General | Website |
| 334 | HIVEonline | For Profit | 7.9 | 11.4 | 9.79 | 9.1 | 9.5475 | https://hiveonline.org/hiv_negative_women/prep_pep_pregnancy_breastfeeding.pdf | N/A | HIV, Prep & PEP | Twitter | Oral | General | Brochure |
| 335 | HIVEonline | For Profit | 7.6 | 9.9 | 10.72 | 9.7 | 9.48 | https://www.hiveonline.org/hiv_negative_women/is_prep_right.pdf | N/A | PrEP | Twitter | Oral | General | Brochure |
| 336 | National Institutes of Health - Office of AIDS Research | The US Government | 9 | 11.4 | 10.21 | 10.6 | 10.3025 | https://hivinfo.nih.gov/understanding-hiv/fact-sheets/basics-hiv-prevention | https://web.archive.org/web/20220425210702/https://hivinfo.nih.gov/understanding-hiv/fact-sheets/basics-hiv-prevention | HIV | Twitter | General PrEP | General | Website |
| 337 | National Institutes of Health - Office of AIDS Research | The US Government | 8.5 | 11 | 10.32 | 10.1 | 9.98 | https://hivinfo.nih.gov/understanding-hiv/fact-sheets/hiv-and-aids-basics | https://web.archive.org/web/20220425210735/https://hivinfo.nih.gov/understanding-hiv/fact-sheets/hiv-and-aids-basics | HIV-AIDS | Twitter | General PrEP | General | Website |
| 338 | National Institutes of Health - Office of AIDS Research | The US Government | 8.4 | 11.4 | 9.45 | 9.2 | 9.6125 | https://hivinfo.nih.gov/understanding-hiv/fact-sheets/post-exposure-prophylaxis-pep | https://web.archive.org/web/20220425212936/https://hivinfo.nih.gov/understanding-hiv/fact-sheets/post-exposure-prophylaxis-pep | HIV | Twitter | General PrEP | General | Website |
| 339 | National Institutes of Health - Office of AIDS Research | The US Government | 9.4 | 11.6 | 10.79 | 10.1 | 10.4725 | https://hivinfo.nih.gov/understanding-hiv/fact-sheets/what-preventive-hiv-vaccine | https://web.archive.org/web/20220425213435/https://hivinfo.nih.gov/understanding-hiv/fact-sheets/what-preventive-hiv-vaccine | HIV | Twitter | General PrEP | General | Website |
| 340 | i-base | For Profit | 6.3 | 9.4 | 7.94 | 7.1 | 7.685 | https://i-base.info/guides/prep | https://web.archive.org/web/20220421212324/https://i-base.info/guides/prep/prep-for-women | PrEP | Twitter | Oral & Injection PrEP | General | Website |
| 341 | i-base | For Profit | 5.9 | 9.9 | 7.35 | 6.3 | 7.3625 | https://i-base.info/qa/factsheets/hiv-transmission-and-testing | https://web.archive.org/web/20220425213557/https://i-base.info/qa/factsheets/hiv-transmission-and-testing | HIV | Twitter | General PrEP | General | Website |
| 342 | i-base | For Profit | 6.1 | 9.1 | 7.12 | 6.3 | 7.155 | https://i-base.info/qa-on-prep-in-the-uk-and-changes-to-the-hiv-proud-study/ | https://web.archive.org/web/20220425213527/https://i-base.info/qa-on-prep-in-the-uk-and-changes-to-the-hiv-proud-study/ | PrEP | Twitter | General PrEP | General | Website |
| 343 | IS PrEP FOR ME | Non Government Organization | 8.1 | 10.6 | 7.54 | 8.5 | 8.685 | https://isprepforme.com/can-i-pass-hiv-if-its-inside-my-body/ | https://web.archive.org/web/20220425213619/https://isprepforme.com/can-i-pass-hiv-if-its-inside-my-body/ | PrEP | Twitter | General PrEP | General | Website |
| 344 | IS PrEP FOR ME | Non Government Organization | 8.7 | 11.7 | 7.89 | 9.5 | 9.4475 | https://isprepforme.com/consent/ | https://web.archive.org/web/20220425213648/https://isprepforme.com/consent/ | PrEP | Twitter | General PrEP | General | Website |
| 345 | IS PrEP FOR ME | Non Government Organization | 10.3 | 11.5 | 6.8 | 10.2 | 9.7 | https://isprepforme.com/consent/on-demand/ | https://web.archive.org/web/20220425213701/https://isprepforme.com/consent/on-demand/ | PrEP | Twitter | Oral | General | Website |
| 346 | IS PrEP FOR ME | Non Government Organization | 6.4 | 10.4 | 9.09 | 7.3 | 8.2975 | https://isprepforme.com/difference-between-intermittent-daily-prep/ | https://web.archive.org/web/20220425213706/https://isprepforme.com/difference-between-intermittent-daily-prep/ | PrEP | Twitter | Oral | General | Website |
| 347 | IS PrEP FOR ME | Non Government Organization | 7.3 | 11.3 | 8.06 | 8.1 | 8.69 | https://isprepforme.com/does-prep-cause-hiv-resistance/ | https://web.archive.org/web/20220425213711/https://isprepforme.com/does-prep-cause-hiv-resistance/ | HIV & PrEP | Twitter | General PrEP | General | Website |
| 348 | IS PrEP FOR ME | Non Government Organization | 6.8 | 11 | 10.83 | 8.8 | 9.3575 | https://isprepforme.com/does-prep-protect-against-stds/ | https://web.archive.org/web/20220425213718/https://isprepforme.com/does-prep-protect-against-stds/ | PrEP | Twitter | General PrEP | General | Website |
| 349 | IS PrEP FOR ME | Non Government Organization | 7.8 | 12.2 | 9.27 | 8.4 | 9.4175 | https://isprepforme.com/does-truvada-cause-osteoporosis/ | https://web.archive.org/web/20220425213818/https://isprepforme.com/does-truvada-cause-osteoporosis/ | PrEP | Twitter | Oral | General | Website |
| 350 | IS PrEP FOR ME | Non Government Organization | 6.1 | 9.4 | 5.46 | 7.2 | 7.04 | https://isprepforme.com/do-i-double-up-my-truvada-if-i-miss-a-tablet/ | https://web.archive.org/web/20220425213824/https://isprepforme.com/do-i-double-up-my-truvada-if-i-miss-a-tablet/ | PrEP | Twitter | Oral | General | Website |
| 351 | IS PrEP FOR ME | Non Government Organization | 4.8 | 9.1 | 4.63 | 4.4 | 5.7325 | https://isprepforme.com/do-i-need-prep-if-im-a-top/ | https://web.archive.org/web/20220425213829/https://isprepforme.com/do-i-need-prep-if-im-a-top/ | PrEP | Twitter | General PrEP | General | Website |
| 352 | IS PrEP FOR ME | Non Government Organization | 7.7 | 12.2 | 10.61 | 9.7 | 10.0525 | https://isprepforme.com/do-i-still-need-condoms-with-prep/ | https://web.archive.org/web/20220425213834/https://isprepforme.com/do-i-still-need-condoms-with-prep/ | PrEP | Twitter | General PrEP | General | Website |
| 353 | IS PrEP FOR ME | Non Government Organization | 5.4 | 8.6 | 4.28 | 4.3 | 5.645 | https://isprepforme.com/do-i-take-truvada-with-food/ | https://web.archive.org/web/20220425213839/https://isprepforme.com/do-i-take-truvada-with-food/ | PrEP | Twitter | Oral | General | Website |
| 354 | IS PrEP FOR ME | Non Government Organization | 9.6 | 12.3 | 8.88 | 11.6 | 10.595 | https://isprepforme.com/dont-take-prep-with-metamucil/ | https://web.archive.org/web/20220425213933/https://isprepforme.com/dont-take-prep-with-metamucil/ | PrEP | Twitter | General PrEP | General | Website |
| 355 | IS PrEP FOR ME | Non Government Organization | 10 | 12.8 | 10.33 | 13.2 | 11.5825 | https://isprepforme.com/drug-interactions-with-truvada/ | https://web.archive.org/web/20220425213940/https://isprepforme.com/drug-interactions-with-truvada/ | Truvada | Twitter | Oral | General | Website |
| 356 | IS PrEP FOR ME | Non Government Organization | 8.7 | 12.2 | 9.16 | 9.3 | 9.84 | https://isprepforme.com/have-people-been-infected-with-hiv-when-taking-prep/ | https://web.archive.org/web/20220425213944/https://isprepforme.com/have-people-been-infected-with-hiv-when-taking-prep/ | PrEP | Twitter | Oral | General | Website |
| 357 | IS PrEP FOR ME | Non Government Organization | 5.7 | 9.4 | 6.83 | 5.5 | 6.8575 | https://isprepforme.com/how-does-prep-block-hiv/ | https://web.archive.org/web/20220425213950/https://isprepforme.com/how-does-prep-block-hiv/ | PrEP | Twitter | General PrEP | General | Website |
| 358 | IS PrEP FOR ME | Non Government Organization | 5.6 | 9.7 | 7.3 | 7.1 | 7.425 | https://isprepforme.com/how-does-prep-work/ | https://web.archive.org/web/20220425213955/https://isprepforme.com/how-does-prep-work/ | PrEP | Twitter | General PrEP | General | Website |
| 359 | IS PrEP FOR ME | Non Government Organization | 6 | 9.6 | 6.55 | 6.6 | 7.1875 | https://isprepforme.com/how-do-i-stop-taking-prep/ | https://web.archive.org/web/20220425214044/https://isprepforme.com/how-do-i-stop-taking-prep/ | PrEP | Twitter | Oral | General | Website |
| 360 | IS PrEP FOR ME | Non Government Organization | 5.7 | 9.2 | 7.82 | 6.6 | 7.33 | https://isprepforme.com/how-often-do-i-need-to-see-the-doctor/ | https://web.archive.org/web/20220425214050/https://isprepforme.com/how-often-do-i-need-to-see-the-doctor/ | PrEP | Twitter | Oral | General | Website |
| 361 | IS PrEP FOR ME | Non Government Organization | 7.3 | 9.4 | 6.96 | 8.4 | 8.015 | https://isprepforme.com/how-to-start-prep-after-stopping/ | https://web.archive.org/web/20220425214055/https://isprepforme.com/how-to-start-prep-after-stopping/ | PrEP | Twitter | Oral | General | Website |
| 362 | IS PrEP FOR ME | Non Government Organization | 9.3 | 11.2 | 7.89 | 10.3 | 9.6725 | https://isprepforme.com/is-prep-just-for-gay-men/ | https://web.archive.org/web/20220425214101/https://isprepforme.com/is-prep-just-for-gay-men/ | PrEP | Twitter | Oral | General | Website |
| 363 | IS PrEP FOR ME | Non Government Organization | 4.8 | 9.6 | 6.19 | 5.4 | 6.4975 | https://isprepforme.com/should-i-increase-my-prep-if-ive-very-sexual/ | https://web.archive.org/web/20220425214154/https://isprepforme.com/should-i-increase-my-prep-if-ive-very-sexual/ | PrEP | Twitter | Oral | General | Website |
| 364 | IS PrEP FOR ME | Non Government Organization | 9.3 | 10.9 | 9.46 | 10.3 | 9.99 | https://isprepforme.com/so-i-can-stop-using-condoms/ | https://web.archive.org/web/20220425214203/https://isprepforme.com/so-i-can-stop-using-condoms/ | PrEP | Twitter | General PrEP | General | Website |
| 365 | IS PrEP FOR ME | Non Government Organization | 8.2 | 10.8 | 6.56 | 9.2 | 8.69 | https://isprepforme.com/travel/ | https://web.archive.org/web/20220425214209/https://isprepforme.com/travel/ | PrEP | Twitter | General PrEP | General | Website |
| 366 | IS PrEP FOR ME | Non Government Organization | 8.2 | 10.3 | 8.3 | 9.3 | 9.025 | https://isprepforme.com/what-are-the-side-effects-of-prep/ | https://web.archive.org/web/20220425214214/https://isprepforme.com/what-are-the-side-effects-of-prep/ | PrEP | Twitter | General PrEP | General | Website |
| 367 | IS PrEP FOR ME | Non Government Organization | 5.9 | 11 | 7.7 | 6.5 | 7.775 | https://isprepforme.com/what-happens-if-i-drink-or-take-drugs/ | https://web.archive.org/web/20220425214218/https://isprepforme.com/what-happens-if-i-drink-or-take-drugs/ | PrEP | Twitter | General PrEP | General | Website |
| 368 | IS PrEP FOR ME | Non Government Organization | 4.7 | 9 | 5.51 | 5.7 | 6.2275 | https://isprepforme.com/what-if-i-miss-a-tablet-of-prep/ | https://web.archive.org/web/20220425214231/https://isprepforme.com/what-if-i-miss-a-tablet-of-prep/ | PrEP | Twitter | Oral | General | Website |
| 369 | IS PrEP FOR ME | Non Government Organization | 9.3 | 13 | 8.53 | 10.8 | 10.4075 | https://isprepforme.com/what-is-prep/ | https://web.archive.org/web/20220420174225/https://isprepforme.com/what-is-prep/ | PrEP | Twitter | Oral | General | Website |
| 370 | IS PrEP FOR ME | Non Government Organization | 6.6 | 10.9 | 8.75 | 8 | 8.5625 | https://isprepforme.com/what-is-the-difference-between-prep-and-tasp/ | https://web.archive.org/web/20220425214341/https://isprepforme.com/what-is-the-difference-between-prep-and-tasp/ | PrEP | Twitter | General PrEP | General | Website |
| 371 | IS PrEP FOR ME | Non Government Organization | 6.6 | 10.5 | 9.03 | 7.3 | 8.3575 | https://isprepforme.com/who-should-take-prep/ | https://web.archive.org/web/20220425214341/https://isprepforme.com/who-should-take-prep/ | PrEP | Twitter | General PrEP | General | Website |
| 372 | IS PrEP FOR ME | Non Government Organization | 5.1 | 10.1 | 6.2 | 5.8 | 6.8 | https://isprepforme.com/will-prep-work-if-i-have-an-sti/ | https://web.archive.org/web/20220425214429/https://isprepforme.com/will-prep-work-if-i-have-an-sti/ | PrEP | Twitter | General PrEP | General | Website |
| 373 | TERRENCE HUGGINS TRUST (BHIVA - Brirish HIV Association) | Non Government Organization | 5.9 | 9.6 | 6.89 | 5.9 | 7.0725 | https://www.iwantprepnow.co.uk/ | https://web.archive.org/web/20220421211346/https://www.iwantprepnow.co.uk/ | PrEP | Twitter | Oral | General | Website |
| 374 | TERRENCE HUGGINS TRUST (BHIVA - Brirish HIV Association) | Non Government Organization | 7 | 10.7 | 8.29 | 7.9 | 8.4725 | https://www.iwantprepnow.co.uk/about/ | https://web.archive.org/web/20220425214358/https://www.iwantprepnow.co.uk/about/ | PrEP | Twitter | Oral | General | Website |
| 375 | TERRENCE HUGGINS TRUST (BHIVA - Brirish HIV Association) | Non Government Organization | 8.7 | 11 | 9.8 | 9.9 | 9.85 | https://www.iwantprepnow.co.uk/who-needs-prep/ | https://web.archive.org/web/20220425214400/https://www.iwantprepnow.co.uk/who-needs-prep/ | PrEP | Twitter | General PrEP | General | Website |
| 376 | Lambda Legal | For Profit | 9.7 | 12.5 | 9.4 | 10.4 | 10.5 | https://www.lambdalegal.org/know-your-rights/article/hiv-prep | https://web.archive.org/web/20220425222135/https://www.lambdalegal.org/know-your-rights/article/hiv-prep | PrEP | Twitter | Oral | General | Website |
| 377 | Local Government Association | Non-US Government/other Public Health Organization | 13.7 | 15.1 | 13.23 | 15.5 | 14.3825 | https://www.local.gov.uk/sites/default/files/documents/1.102%20PrEP%20faqs_05.pdf | N/A | PrEP | Twitter | General PrEP | General | Information Sheet |
| 378 | MARIEN APOTHEKE WIEN | For Profit | 10 | 12.5 | 11.31 | 11.2 | 11.2525 | https://www.marienapo.eu/hiv/prophylaxe-und-therapie/faqs-zur-prep/ | https://web.archive.org/web/20220419231525/https://www.marienapo.eu/hiv/prophylaxe-und-therapie/faqs-zur-prep/ | PrEP | Twitter | Oral | General | Website |
| 379 | NATIONAL AIDS TRUST | For Profit | 11.8 | 12.1 | 8.36 | 13.6 | 11.465 | https://www.nat.org.uk/about-hiv/do-i-have-hiv | https://web.archive.org/web/20220421011634/https://www.nat.org.uk/about-hiv/do-i-have-hiv | HIV | Twitter | General PrEP | General | Website |
| 380 | NATIONAL ASSOCIATION OF SOCIAL WORKERS | Non Government Organization | 13.8 | 15.1 | 12.94 | 15.4 | 14.31 | http://www.socialworkblog.org/practice-and-professional-development/health-care/2014/05/social-work-practice-update-cdc-releases-practice-guidelines-for-pre-exposure-prophylaxis-prep/ | https://web.archive.org/web/20220420011800/http://www.socialworkblog.org/practice-and-professional-development/health-care/2014/05/social-work-practice-update-cdc-releases-practice-guidelines-for-pre-exposure-prophylaxis-prep/ | PrEP | Twitter | General PrEP | General | Website |
| 381 | National Institute of Allergies and Infectious Diseases | The US Government | 10.6 | 13.4 | 13.87 | 13.7 | 12.8925 | https://www.niaid.nih.gov/diseases-conditions/10-things-know-about-hiv-suppression | https://web.archive.org/web/20220421213627/https://www.niaid.nih.gov/diseases-conditions/10-things-know-about-hiv-suppression | HIV | Twitter | General PrEP | General | Website |
| 382 | NEW YORK STATE DEPARTMENT OF HEALTH AIDS INSTITUTE - CLINICAL GUIDELINES PROGRAM | The US Government | 7.1 | 10.1 | 8.35 | 8.2 | 8.4375 | https://www.hivguidelines.org/prep-for-prevention/selected-resources-prep/#tab_1 | https://web.archive.org/web/20220420172737/https://www.hivguidelines.org/prep-for-prevention/selected-resources-prep/ | HIV & PrEP | Twitter | General PrEP | General | Website |
| 383 | Nola Health Link | The US Government | 8 | 11.3 | 9.79 | 9.3 | 9.5975 | https://www.nolahealthlink.com/lets-talk-about-prep/prep-faqs/ | https://web.archive.org/web/20220419210520/https://www.nolahealthlink.com/lets-talk-about-prep/prep-faqs/ | PrEP | Twitter | Oral | General | Website |
| 384 | NYC Health | The US Government | 11.7 | 14.6 | 14.45 | 14.4 | 13.7875 | https://www1.nyc.gov/assets/doh/downloads/pdf/csi/csi-prep-hcp-faq.pdf | N/A | PrEP | Twitter | Oral | Provider | Information Sheet |
| 385 | Office on Women's Health - U.S. Department of Health and Human Services | The US Government | 8.1 | 10 | 9.23 | 10 | 9.3325 | https://www.womenshealth.gov/hiv-and-aids/hiv-and-aids-basics/how-hiv-spread | https://web.archive.org/web/20220425222357/https://www.womenshealth.gov/hiv-and-aids/hiv-and-aids-basics/how-hiv-spread | HIV | Twitter | General PrEP | General | Website |
| 386 | Office on Women's Health - U.S. Department of Health and Human Services | The US Government | 7.6 | 10.6 | 9.22 | 9.5 | 9.23 | https://www.womenshealth.gov/hiv-and-aids/hiv-prevention | https://web.archive.org/web/20220425222400/https://www.womenshealth.gov/hiv-and-aids/hiv-prevention | HIV | Twitter | General PrEP | General | Website |
| 387 | OUT HERE - APLA HEALTH | The US Government | 6.2 | 10.5 | 9.1 | 7.9 | 8.425 | https://outherehealth.com/prep/ | https://web.archive.org/web/20220421161724/https://outherehealth.com/prep/ | PrEP | Twitter | General PrEP | General | Website |
| 388 | PleasePrEPme | Non Government Organization | 7.1 | 10.2 | 8.87 | 8.5 | 8.6675 | https://www.pleaseprepme.org/sites/default/files/file-attachments/PrEP%20for%20MSM%20-%20Is%20taking%20PrEP%20the%20right%20choice%20for%20you%3F_en.pdf | N/A | PrEP | Twitter | Oral | General | Brochure |
| 389 | POZ | For Profit | 9.9 | 12.5 | 10.21 | 11.5 | 11.0275 | https://www.poz.com/article/hiv-prep-questions-22701-6459#.UBU-XUnPFZU.twitter | https://web.archive.org/web/20220419224923/https://www.poz.com/article/hiv-prep-questions-22701-6459 | PrEP & Truvada | Twitter | Oral | General | Website |
| 390 | PrEP Daily | Non Government Organization | 9.2 | 11.9 | 9.22 | 9.9 | 10.055 | https://prepdaily.org/5-questions-to-ask-your-doctor-about-prep-for-hiv-prevention/ | https://web.archive.org/web/20220425222453/https://prepdaily.org/5-questions-to-ask-your-doctor-about-prep-for-hiv-prevention/ | HIV & PrEP | Twitter | Oral | General | Website |
| 391 | PrEP Daily | Non Government Organization | 9.1 | 12 | 10.61 | 10.9 | 10.6525 | https://prepdaily.org/hiv-and-family-planning-answering-the-faqs/ | https://web.archive.org/web/20210707152231/https://prepdaily.org/hiv-and-family-planning-answering-the-faqs/ | HIV & PrEP | Twitter | General PrEP | General | Website |
| 392 | PrEP Daily | Non Government Organization | 10.3 | 12.8 | 11.31 | 11.5 | 11.4775 | https://prepdaily.org/what-you-need-to-know-about-the-new-hiv-prevention-medication-apretude/ | https://web.archive.org/web/20220425223007/https://prepdaily.org/what-you-need-to-know-about-the-new-hiv-prevention-medication-apretude/ | HIV | Twitter | Injection | General | Website |
| 393 | PrEP IN EUROPE | Non Government Organization | 10.9 | 13.1 | 10.16 | 12.5 | 11.665 | https://www.prepineurope.org/wp-content/uploads/2019/04/PrEP-in-Europe-fact-sheet-03.2019.pdf | https://web.archive.org/web/20220425222433/https://outherehealth.com/pep/ | HIV & PrEP | Twitter | Oral & Injection PrEP | General | Information Sheet |
| 394 | PrEP&Prejudice | Non Government Organization | 9.9 | 12.6 | 10.73 | 11.9 | 11.2825 | https://www.prepandprejudice.org.uk/about-prep | https://web.archive.org/web/20220421012118/https://www.prepandprejudice.org.uk/about-prep | PrEP | Twitter | Oral | General | Website |
| 395 | PrEP.scot - NHS | Non-US Government/other Public Health Organization | 6.3 | 9.5 | 7.65 | 7.9 | 7.8375 | https://www.prep.scot/about-prep/ | https://web.archive.org/web/20220420204303/https://www.prep.scot/about-prep/ | HIV & PrEP | Twitter | Oral | General | Website |
| 396 | PrEP.scot - NHS | Non-US Government/other Public Health Organization | 5.5 | 9.2 | 7.64 | 6.1 | 7.11 | https://www.prep.scot/who-should-take-prep/bme/ | https://web.archive.org/web/20220425223314/https://www.prep.scot/who-should-take-prep/bme/ | HIV & PrEP | Twitter | General PrEP | General | Website |
| 397 | PrEP.scot - NHS | Non-US Government/other Public Health Organization | 8.5 | 11.9 | 8.87 | 8.6 | 9.4675 | https://www.prep.scot/who-should-take-prep/gbmsm/ | https://web.archive.org/web/20220425223316/https://www.prep.scot/who-should-take-prep/gbmsm/ | HIV & PrEP | Twitter | General PrEP | General | Website |
| 398 | PrEP.scot - NHS | Non-US Government/other Public Health Organization | 6 | 9.9 | 9.67 | 8.1 | 8.4175 | https://www.prep.scot/who-should-take-prep/inject-drugs/ | https://web.archive.org/web/20220425223320/https://www.prep.scot/who-should-take-prep/inject-drugs/ | HIV & PrEP | Twitter | General PrEP | General | Website |
| 399 | PrEP.scot - NHS | Non-US Government/other Public Health Organization | 8.9 | 12 | 7.07 | 7.7 | 8.9175 | https://www.prep.scot/who-should-take-prep/serodiscordant/ | https://web.archive.org/web/20220425223324/https://www.prep.scot/who-should-take-prep/serodiscordant/ | HIV & PrEP | Twitter | General PrEP | General | Website |
| 400 | PrEP.scot - NHS | Non-US Government/other Public Health Organization | 11.7 | 13.3 | 10.39 | 13.5 | 12.2225 | https://www.prep.scot/who-should-take-prep/sex-for-payment/ | https://web.archive.org/web/20220425223331/https://www.prep.scot/who-should-take-prep/sex-for-payment/ | HIV | Twitter | General PrEP | General | Website |
| 401 | PrEP.scot - NHS | Non-US Government/other Public Health Organization | 8.6 | 11.9 | 10.55 | 9 | 10.0125 | https://www.prep.scot/who-should-take-prep/trans-and-non-binary-people/ | https://web.archive.org/web/20220425223445/https://www.prep.scot/who-should-take-prep/trans-and-non-binary-people/ | HIV | Twitter | General PrEP | General | Website |
| 402 | PrEP'D For Change | Non Government Organization | 7.5 | 10.8 | 9.8 | 9.8 | 9.475 | https://www.prepdforchange.com/. https://www.prepdforchange.com/choose-prep | https://web.archive.org/web/20220420201600/https://www.prepdforchange.com/ | PrEP | Twitter | General PrEP | General | Website |
| 403 | PrEP'D For Change | Non Government Organization | 8.7 | 11.1 | 10.26 | 10.2 | 10.065 | https://www.prepdforchange.com/access-prep | https://web.archive.org/web/20220425223515/https://www.prepdforchange.com/access-prep | PrEP | Twitter | General PrEP | General | Website |
| 404 | PrEP'D For Change | Non Government Organization | 7.7 | 11 | 8.81 | 9.3 | 9.2025 | https://www.prepdforchange.com/faqs | https://web.archive.org/web/20220425223533/https://www.prepdforchange.com/faqs | PrEP | Twitter | Oral | General | Website |
| 405 | PrEP'D For Change | Non Government Organization | 8.7 | 11.4 | 8.76 | 10.2 | 9.765 | https://www.prepdforchange.com/use-prep | https://web.archive.org/web/20220425223539/https://www.prepdforchange.com/use-prep | PrEP | Twitter | Oral | General | Website |
| 406 | Prepster | For Profit | 6.5 | 10 | 8.52 | 7.8 | 8.205 | https://prepster.info/faq/ | https://web.archive.org/web/20220419223003/https://prepster.info/faq/ | PrEP | Twitter | Oral | General | Website |
| 407 | Prepster | For Profit | 6.8 | 9.8 | 9.21 | 8.6 | 8.6025 | https://prepster.info/tasp/ | https://web.archive.org/web/20220425223605/https://prepster.info/tasp/ | HIV & PrEP | Twitter | General PrEP | General | Website |
| 408 | Prepster | For Profit | 8.9 | 12.3 | 11.02 | 11.3 | 10.88 | https://prepster.info/wp-content/uploads/2018/06/PrEP-QA-booklet-e-use.pdf | N/A | PrEP | Twitter | Oral | General | Website |
| 409 | prevention access campaign | Non Government Organization | 11.3 | 13 | 11.78 | 13.1 | 12.295 | https://preventionaccess.org/faq/ | https://web.archive.org/web/20220419190810/https://preventionaccess.org/faq/ | HIV | Twitter | General PrEP | General | Website |
| 410 | QOOH.me | Non Government Organization | 6.5 | 9.9 | 6.48 | 5.2 | 7.02 | https://qooh.me/question/question/qid/11009868/ | https://web.archive.org/web/20220419224825/https://qooh.me/question/question/qid/11009868/ | Truvada | Twitter | Oral | General | Website |
| 411 | QOOH.me | Non Government Organization | 7 | 10.5 | 7.99 | 6.9 | 8.0975 | https://qooh.me/question/question/qid/25933232/ | https://web.archive.org/web/20220425223655/https://qooh.me/question/question/qid/25933232/ | Truvada | Twitter | Oral | General | Website |
| 412 | QUIERO SABER - American Sexual Health Association | Non Government Organization | 8.4 | 11.3 | 9.69 | 10.7 | 10.0225 | https://www.quierosaber.org/ets/vih-sida | https://web.archive.org/web/20220421175748/https://www.quierosaber.org/ets/vih-sida | HIV | Twitter | General PrEP | General | Website |
| 413 | San Francisco AIDS Foundation | Non Government Organization | 9.4 | 10.5 | 7.66 | 10.5 | 9.515 | https://www.sfaf.org/collections/beta/prep-facts-what-are-the-ways-to-take-prep/#slide4 | https://web.archive.org/web/20220425224703/https://www.sfaf.org/collections/beta/prep-facts-what-are-the-ways-to-take-prep/ | PrEP | Twitter | Oral & Injection PrEP | General | Website |
| 414 | San Francisco AIDS Foundation | Non Government Organization | 9.1 | 10.8 | 10.73 | 11.2 | 10.4575 | https://www.sfaf.org/collections/beta/prep-facts-where-can-i-get-prep/ | https://web.archive.org/web/20220425224716/https://www.sfaf.org/collections/beta/prep-facts-where-can-i-get-prep/ | PrEP | Twitter | General PrEP | General | Website |
| 415 | San Francisco AIDS Foundation | Non Government Organization | 8.6 | 11.1 | 9.8 | 9.7 | 9.8 | https://www.sfaf.org/resource-library/prep/ | https://web.archive.org/web/20220425224825/https://www.sfaf.org/resource-library/prep/ | PrEP | Twitter | Oral | General | Website |
| 416 | San Francisco AIDS Foundation | Non Government Organization | 9 | 10 | 8.19 | 12 | 9.7975 | https://www.sfaf.org/services/prep-pep/prep-2-1-1/ | https://web.archive.org/web/20220425224831/https://www.sfaf.org/services/prep-pep/prep-2-1-1/ | PrEP | Twitter | Oral | General | Website |
| 417 | San Francisco AIDS Foundation | Non Government Organization | 6.2 | 9.8 | 7.65 | 7.1 | 7.6875 | https://www.sfaf.org/wp-content/uploads/PrEP-211-study-info-flyer-ENGLISH-final.pdf | N/A | PrEP | Twitter | Oral | General | Website |
| 418 | San Francisco City Clinic | Non Government Organization | 7.5 | 10.9 | 9.05 | 9.4 | 9.2125 | https://www.sfcityclinic.org/faqs | https://web.archive.org/web/20220421212849/https://www.sfcityclinic.org/faqs | STIs & HIV | Twitter | General PrEP | General | Website |
| 419 | SAY Yes TO PrEP - American Sexual Health Association | For Profit | 9.4 | 11.9 | 9.4 | 10.3 | 10.25 | http://www.sayyestoprep.org/faqs/ | https://web.archive.org/web/20220421175117/http://www.sayyestoprep.org/faqs/ | PrEP & PEP | Twitter | General PrEP | General | Website |
| 420 | sexualwellbeing - HSE | Non-US Government/other Public Health Organization | 9.2 | 11.7 | 9.74 | 10.4 | 10.26 | https://www.sexualwellbeing.ie/sexual-health/prep/prep-in-other-languages/hiv-prep-leaflet-english.pdf | N/A | HIV & PrEP | Twitter | Oral | General | Brochure |
| 421 | TERRENCE HUGGINS TRUST (BHIVA - Brirish HIV Association) | Non Government Organization | 8.9 | 11.1 | 9.57 | 9.9 | 9.8675 | https://www.tht.org.uk/hiv-and-sexual-health/being-diagnosed-hiv/newly-diagnosed | https://web.archive.org/web/20220420203144/https://www.tht.org.uk/hiv-and-sexual-health/being-diagnosed-hiv/newly-diagnosed | HIV, PrEP & PEP, Stigma | Twitter | General PrEP | General | Website |
| 422 | THE ADVOCATE | For Profit | 9.4 | 12.7 | 11.2 | 11.5 | 11.2 | https://www.advocate.com/current-issue/2017/11/14/3-most-common-questions-about-prep | https://web.archive.org/web/20220419230146/https://www.advocate.com/current-issue/2017/11/14/3-most-common-questions-about-prep | PrEP | Twitter | General PrEP | General | Website |
| 423 | The American College of Obstetricians and Gynecologists | For Profit | 6.2 | 9.7 | 8.17 | 7.1 | 7.7925 | https://www.acog.org/womens-health/faqs/preventing-hiv-with-medication?utm_source=redirect&utm_medium=web&utm_campaign=otn | https://web.archive.org/web/20220421180744/https://www.acog.org/womens-health/faqs/preventing-hiv-with-medication | HIV & PrEP | Twitter | General PrEP | General | Website |
| 424 | the PROJECT | For Profit | 7.6 | 11 | 9.27 | 8.3 | 9.0425 | https://tpqcblog.org/2020/07/14/7-common-questions-about-prep/ | https://web.archive.org/web/20220419232001/https://tpqcblog.org/contact/ | PrEP | Twitter | Oral | General | Website |
| 425 | TheBody | N/A | 7.6 | 10.6 | 9.16 | 9.6 | 9.24 | https://www.thebody.com/article/pre-exposure-prophylaxis-prep-fact-sheet?ap=sakm | https://web.archive.org/web/20220421012419/https://www.thebody.com/article/pre-exposure-prophylaxis-prep-fact-sheet?ap=sakm | PrEP | Twitter | General PrEP | General | Website |
| 426 | TheBody | N/A | 9.6 | 11.5 | 10.15 | 11.2 | 10.6125 | https://www.thebody.com/health/hiv-transmission-risk | https://web.archive.org/web/20220425230735/https://www.thebody.com/health/hiv-transmission-risk | HIV | Twitter | General PrEP | General | Website |
| 427 | U.S. Preventive Services Task Force | The US Government | 5.6 | 9 | 7.47 | 7.2 | 7.3175 | https://www.uspreventiveservicestaskforce.org/uspstf/sites/default/files/inline-files/hiv-prep-guide-2020_0.pdf | N/A | PrEP | Twitter | Oral | General | Brochure |
| 428 | umbrella health | Non Government Organization | 9.3 | 11.3 | 11.89 | 11.4 | 10.9725 | https://umbrellahealth.co.uk/hiv-and-aids/hiv-faqs/ | https://web.archive.org/web/20220419185226/https://umbrellahealth.co.uk/hiv-and-aids/hiv-faqs/ | HIV-AIDS & PrEP | Twitter | General PrEP | General | Website |
| 429 | umbrella health | Non Government Organization | 10.7 | 11.8 | 9.34 | 11.7 | 10.885 | https://umbrellahealth.co.uk/hiv-and-aids/how-to-avoid-hiv/ | https://web.archive.org/web/20220425233352/https://umbrellahealth.co.uk/hiv-and-aids/how-to-avoid-hiv/ | HIV | Twitter | General PrEP | General | Website |
| 430 | umbrella health | Non Government Organization | 34.5 | 0 | 10.06 | 41.7 | 21.565 | https://umbrellahealth.co.uk/hiv-and-aids/prep/who-should-consider-prep/ | https://web.archive.org/web/20220425233358/https://umbrellahealth.co.uk/hiv-and-aids/prep/who-should-consider-prep/ | PrEP | Twitter | General PrEP | General | Website |
| 431 | UNAIDS | Non-US Government/other Public Health Organization | 12.5 | 14.8 | 13.87 | 14.7 | 13.9675 | https://www.unaids.org/en/resources/documents/2015/Oral_pre-exposure_prophylaxis_questions_and_answers | https://web.archive.org/web/20220420011535/https://www.unaids.org/en/resources/documents/2015/Oral_pre-exposure_prophylaxis_questions_and_answers | HIV & PrEP | Twitter | Oral | General | Website |
| 432 | USA Today News | For Profit | 7.9 | 10.5 | 9.39 | 9.1 | 9.2225 | https://www.usatoday.com/in-depth/news/world/2018/11/30/world-aids-day-2018-30-hiv-aids-facts-and-faqs-30th-anniversary/2150044002/ | https://web.archive.org/web/20220419230557/https://www.usatoday.com/in-depth/news/world/2018/11/30/world-aids-day-2018-30-hiv-aids-facts-and-faqs-30th-anniversary/2150044002/ | HIV-AIDS, PrEP & PEP | Twitter | General PrEP | General | Website |
| 433 | Vermont CARES | Non Government Organization | 8.5 | 11.8 | 10.26 | 10.1 | 10.165 | https://vtcares.org/prevention/your-guide-to-prep-pre-exposure-prophylaxis/ | https://web.archive.org/web/20210416162155/https://vtcares.org/prevention/your-guide-to-prep-pre-exposure-prophylaxis/ | PrEP | Twitter | Oral | General | Website |
| 434 | verywellhealth | Non Government Organization | 12.1 | 13.6 | 11.61 | 13.8 | 12.7775 | http://www.google.com/url?sa=X&q=http://aids.about.com/b/2011/01/30/cdc-issues-guidelines-for-using-truvada-to-prevent-hiv.htm&ct=ga&cad=CAcQARgAIAAoATAAOABAkoCY6gRIAVAAWABiBWVuLVVT&cd=HffmsKj8AuA&usg=AFQjCNHiNGGJ4-Ukhg0sZBqHT1-8ERyEGg | https://web.archive.org/web/20220425233639/https://www.verywellhealth.com/how-effective-is-prep-in-preventing-hiv-4010575 | PrEP | Twitter | Oral | General | Website |
| 435 | verywellhealth | Non Government Organization | 11.4 | 13 | 11.72 | 13.3 | 12.355 | https://www.verywellhealth.com/hiv-aids-5216828#toc-living-with-hiv | https://web.archive.org/web/20220425233950/https://www.verywellhealth.com/hiv-aids-5216828 | HIV-AIDS | Twitter | General PrEP | General | Website |
| 436 | verywellhealth | Non Government Organization | 10.8 | 13.1 | 11.78 | 12.5 | 12.045 | https://www.verywellhealth.com/hiv-prevention-plan-to-reduce-risk-49177 | https://web.archive.org/web/20220425234222/https://www.verywellhealth.com/hiv-prevention-plan-to-reduce-risk-49177 | HIV | Twitter | General PrEP | General | Website |
| 437 | verywellhealth | Non Government Organization | 9.1 | 12.2 | 10.44 | 11 | 10.685 | https://www.verywellhealth.com/hiv-risk-without-ejaculation-during-sex-48788 | https://web.archive.org/web/20220425234227/https://www.verywellhealth.com/hiv-risk-without-ejaculation-during-sex-48788 | HIV | Twitter | General PrEP | General | Website |
| 438 | verywellhealth | Non Government Organization | 8.1 | 11.1 | 9.52 | 10.1 | 9.705 | https://www.verywellhealth.com/how-long-does-it-take-to-show-symptoms-of-hiv-5197749 | https://web.archive.org/web/20220425234232/https://www.verywellhealth.com/how-long-does-it-take-to-show-symptoms-of-hiv-5197749 | HIV | Twitter | General PrEP | General | Website |
| 439 | verywellhealth | Non Government Organization | 10.3 | 12.3 | 12.18 | 12.5 | 11.82 | https://www.verywellhealth.com/truvada-emtricitabine-tenofovir-disoproxil-fumarate-oral-5209014#toc-frequently-asked-questions-daf24a70-fdf3-44fe-9572-d346e57946aa | https://web.archive.org/web/20220425234358/https://www.verywellhealth.com/truvada-emtricitabine-tenofovir-disoproxil-fumarate-oral-5209014 | Truvada | Twitter | Oral | General | Website |
| 440 | WAAC | Non Government Organization | 8.1 | 10 | 8.88 | 9.5 | 9.12 | https://www.waac.com.au/learn/hiv/ | https://web.archive.org/web/20220420231508/https://www.waac.com.au/learn/hiv/ | HIV | Twitter | General PrEP | General | Website |
| 441 | WAAC | Non Government Organization | 8.8 | 11.3 | 10.09 | 10.2 | 10.0975 | https://www.waac.com.au/learn/living-with-hiv/treatment/ | https://web.archive.org/web/20220425234603/https://www.waac.com.au/learn/living-with-hiv/treatment/ | HIV | Twitter | General PrEP | General | Website |
| 442 | Women and PrEP | For Profit | 7.3 | 10 | 8.29 | 8.5 | 8.5225 | https://www.womenandprep.org.uk/hiv-101 | https://web.archive.org/web/20220425234635/https://www.womenandprep.org.uk/hiv-101 | HIV | Twitter | General PrEP | General | Website |
| 443 | Women and PrEP | For Profit | 7.4 | 10.1 | 7.83 | 8.1 | 8.3575 | https://www.womenandprep.org.uk/what-is-prep | https://web.archive.org/web/20220421181759/https://www.womenandprep.org.uk/what-is-prep | HIV & PrEP | Twitter | Oral | General | Website |
| 444 | Workit Health | For Profit | 7.4 | 10.8 | 9.16 | 9.3 | 9.165 | https://www.workithealth.com/blog/answering-your-questions-about-prep-for-hiv/?utm_content=172801886&utm_medium=social&utm_source=twitter&hss_channel=tw-2930160344 | https://web.archive.org/web/20220419233856/https://www.workithealth.com/blog/answering-your-questions-about-prep-for-hiv/?hss_channel=tw-2930160344 | PrEP | Twitter | Oral | General | Website |
| 445 | World Health Organization | Non-US Government/other Public Health Organization | 16.9 | 17.8 | 16.6 | 20.8 | 18.025 | https://www.who.int/emergencies/diseases/novel-coronavirus-2019/question-and-answers-hub/q-a-detail/coronavirus-disease-covid-19-hiv-and-antiretrovirals | https://web.archive.org/web/20220409011817/https://www.who.int/emergencies/diseases/novel-coronavirus-2019/question-and-answers-hub/q-a-detail/coronavirus-disease-covid-19-hiv-and-antiretrovirals | HIV & COVID-19 | Twitter | General PrEP | General | Website |
| 446 | World Health Organization | Non-US Government/other Public Health Organization | 11.1 | 13.2 | 12.24 | 13.2 | 12.435 | https://www.who.int/news-room/questions-and-answers/item/hiv-aids | https://web.archive.org/web/20220425234651/https://www.who.int/news-room/questions-and-answers/item/hiv-aids | HIV-AIDS | Twitter | General PrEP | General | Website |
