# Supplementary figures and images for: Health Information on Pre-Exposure Prophylaxis From Search Engines and Twitter: Readability Analysis
Source: JMIR Public Health Surveill. 2023 Sep 4;9:e48630. doi: 10.2196/48630 (PMC10507523; doi:10.2196/48630)

Information sheet


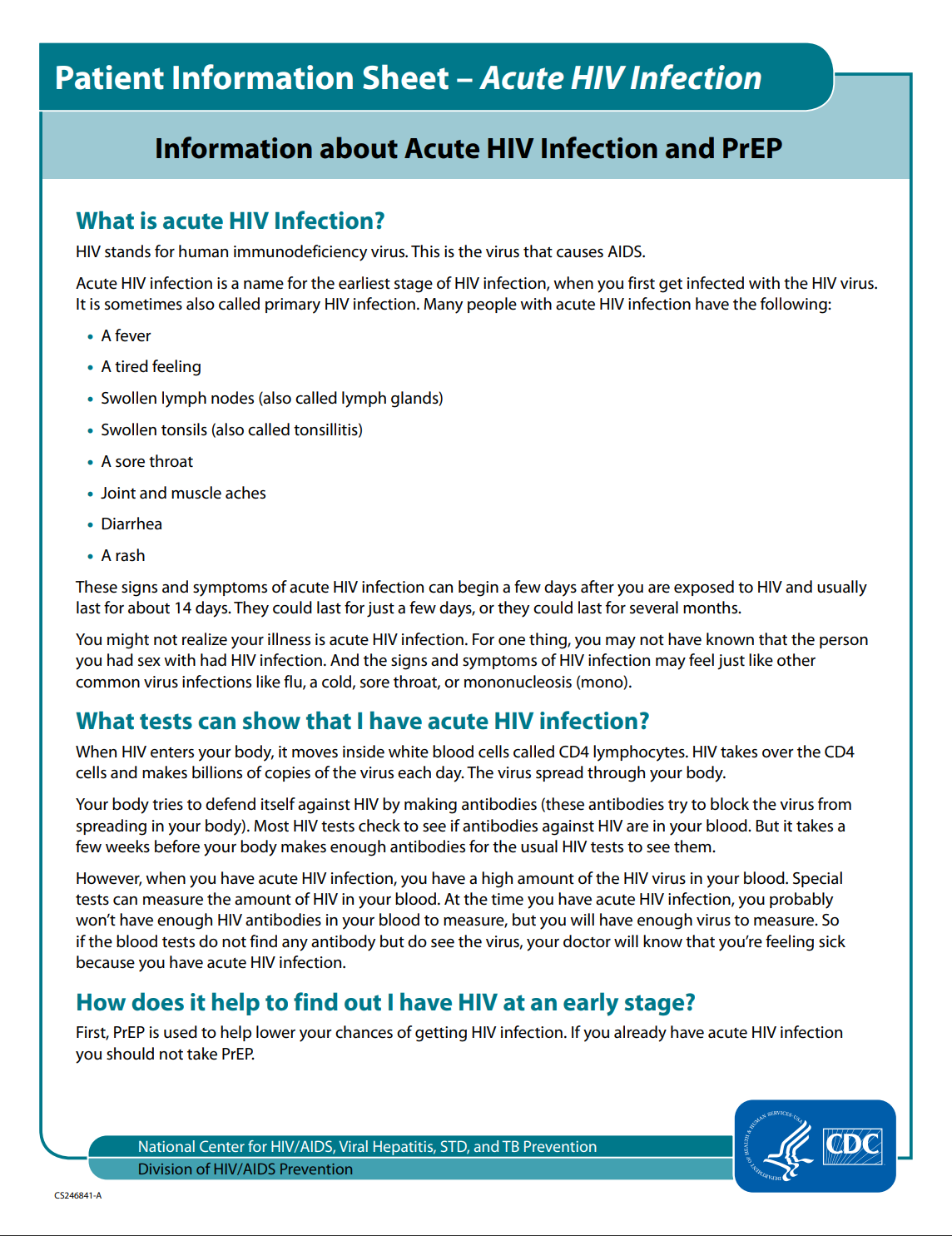


Brochure


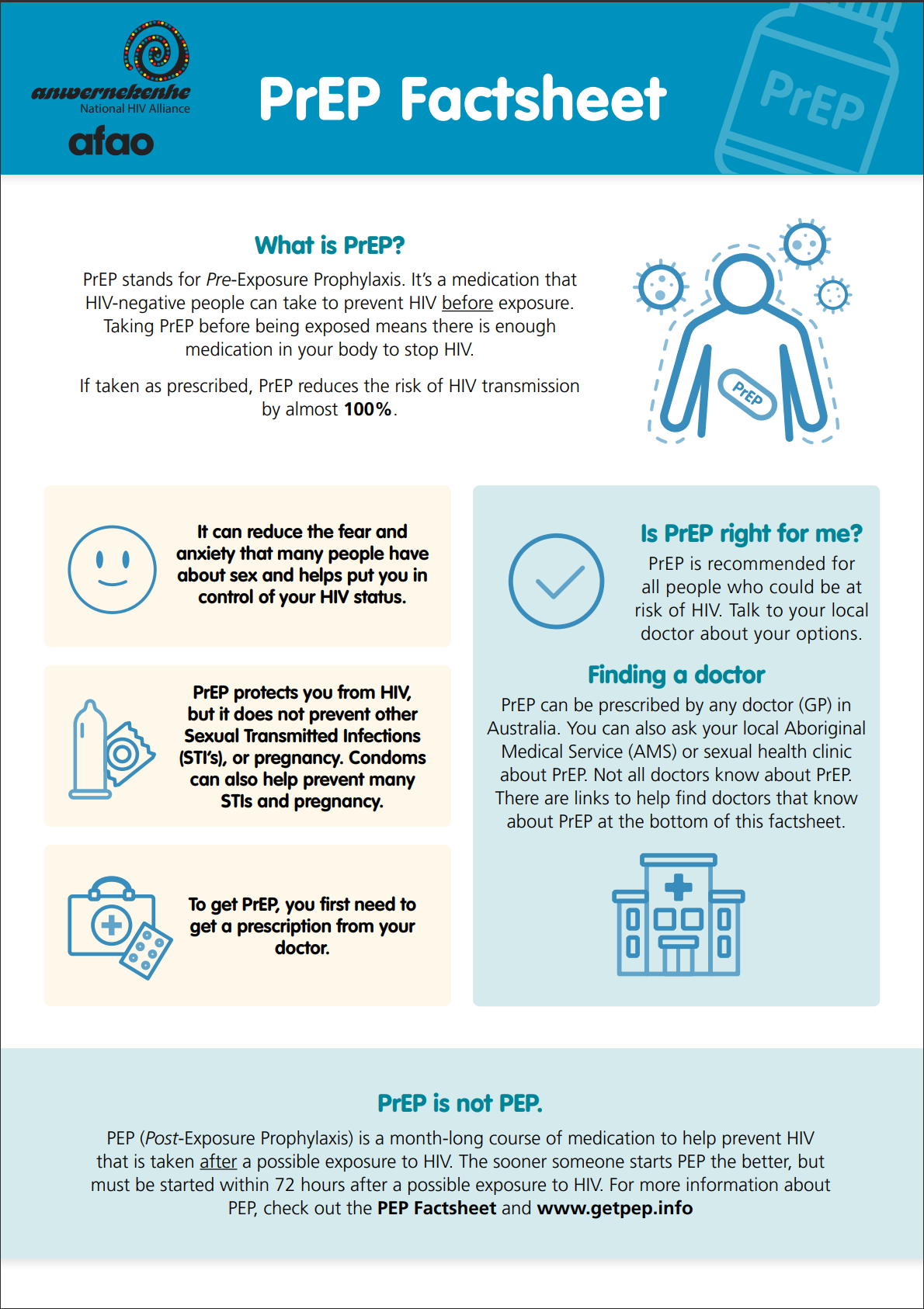


Website


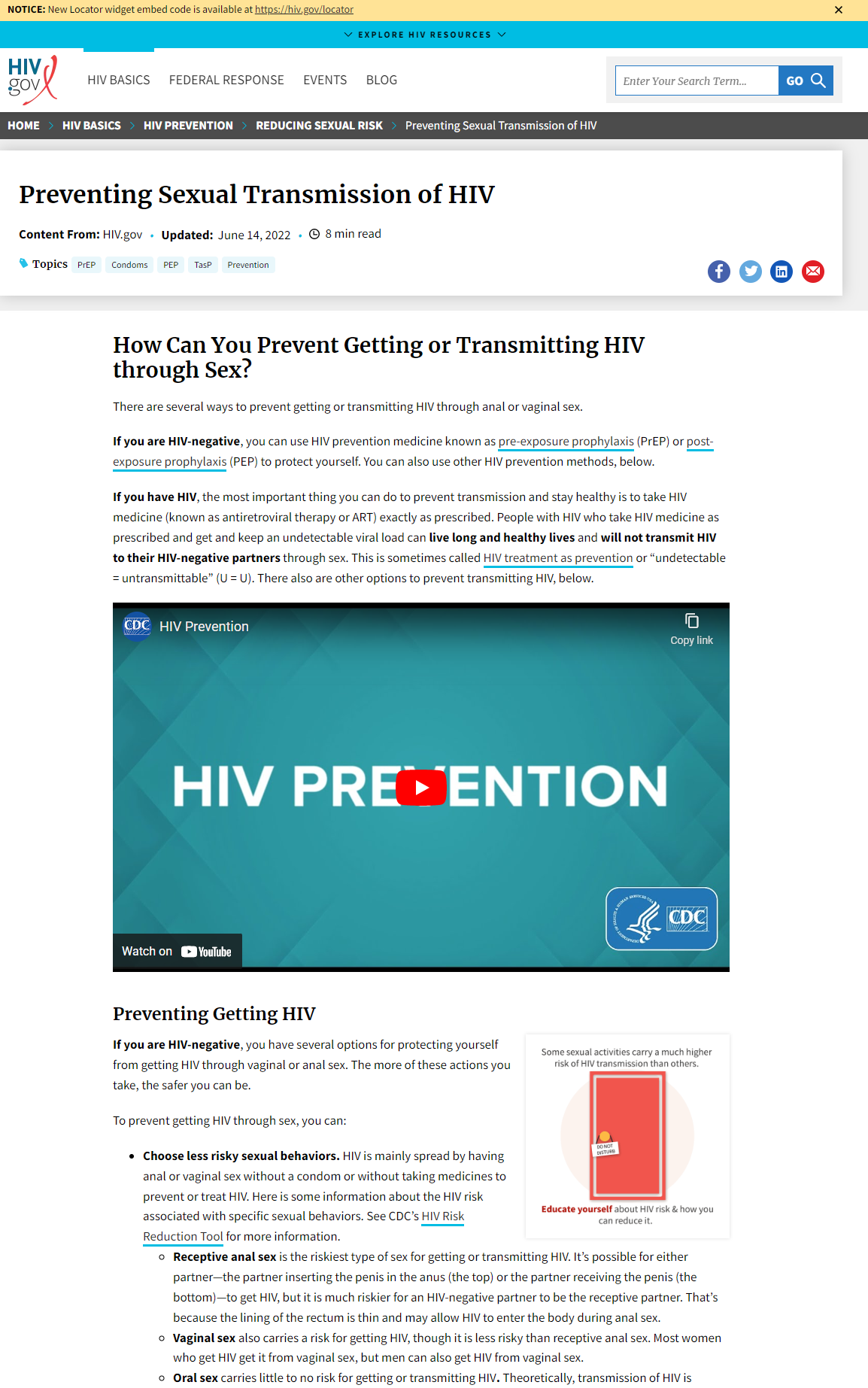


Website (in a blog format)


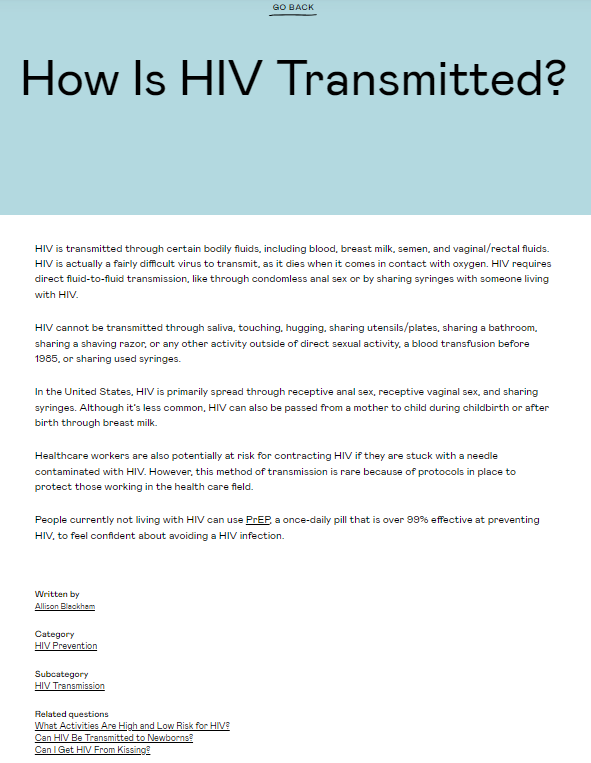

Supplement: Multimedia Appendix 2 [file publichealth_v9i1e48630_app2.docx]
